# Ton‐Scale Industrial Optical Polycarbonate Film: Combining Full‐Color Phosphorescence and Various Photochromic

**DOI:** 10.1002/advs.202517170

**Published:** 2025-09-30

**Authors:** Peng Chen, Jielei Jin, Yanke Zhu, Yunxiang Lei, Xinyang Ye, Wenbo Dai, Chuangjie Gu, Miaochang Liu, Xiaobo Huang, Dan Wang

**Affiliations:** ^1^ School of Chemistry and Materials Engineering Wenzhou University Wenzhou 325035 P. R. China; ^2^ Department of Pediatrics The First Affiliated Hospital of Wenzhou Medical University Wenzhou 325035 P. R. China; ^3^ Key Lab of Biohealth Materials and Chemistry of Wenzhou Wenzhou 325035 P. R. China

**Keywords:** large‐area organic film, photochromic property, Polycarbonate host matrix, room temperature phosphorescence

## Abstract

Polymer Polycarbonate **(PC)** is widely used in fields such as electronics, automobiles, building materials, and packaging is one of the most common plastics in human life. Herein, **PC** is used as the host to achieve doped films with both phosphorescence and photochromic properties. Importantly, the **PC** host has very excellent universality, can be paired with dozens of phosphorescence guests and a series of photochromic guests, achieving full‐color phosphorescence emission from blue to red, as well as rapid, sensitive, and reversible transitions from colorless to pink, red, or blue–purple. More surprisingly, compared to the thirteen commonly used host matrices, extensive data show that **PC**‐based doped materials have the best phosphorescence and photochromic properties, meaning that **PC** is the optimal host among them. Finally, large‐scale industrial preparation of doped film is achieved at the ton level, and the prepared doped film still has excellent optical properties, which can be directly used for product packaging. This work not only discovered that **PC** polymer is an excellent and overlooked host matrix, but also developed optical functional materials with direct commercial application value.

## Graphical abstract

PC is used as the host to achieve doped films with both phosphorescence and photochromic properties, PC can be paired with dozens of phosphorescence guests and photochromic guests, achieving full‐color phosphorescence emission from blue to red, as well as rapid and reversible transitions from colorless to pink, red, or blue–purple.

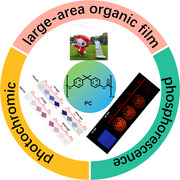

## 1 Introduction

Polymer‐based organic optical films have unique application advantages in organic flexible electronic devices and displays, advanced anti‐counterfeiting and information storage, due to their excellent flexibility, stretchability, and plasticity.^[^ ^1^, ^2^, ^3^, ^4^, ^5^, ^6^, ^7^, ^8^, ^9^, ^10^, ^11^, ^12^ ^]^ Among numerous polymer materials, polycarbonate (**PC**) has excellent mechanical properties, transparency, and heat resistance, is widely used in electronic product casings, optical components, automotive industry, packaging printing, and more.^[^ ^13^, ^14^ ^]^ The global usage of **PC** is approximately five million tons per year, making it one of the most commonly used polymers in human society. However, traditional **PC**‐based materials face bottlenecks such as low technological content, low‐end usage scenarios, and single functionality.^[^ ^15^, ^16^ ^]^ Therefore, endowing **PC**‐based materials with optical properties will greatly enhance their economic value, which has significant practical significance. Among optical phenomena, room temperature phosphorescence (RTP) and photochromism are two distinct but widely applicable optical properties. RTP performance are widely used in the field of anti‐counterfeiting, biological diagnosis and treatment, optoelectronic devices due to the persistent afterglow phenomenon caused by the intersystem‐crossing process of excitons.^[^ ^17^, ^18^, ^19^, ^20^, ^21^ ^]^ While photochromic property refers to the sensitive and significant changes in the appearance color of materials under light stimulation, which is also widely used in optical switches, advanced anti‐counterfeiting, optical data storage.^[^ ^22^, ^23^, ^24^, ^25^, ^26^ ^]^ Therefore, if **PC** polymers can be endowed with both photochromic and RTP properties, it will greatly enhance their technological value and expand their application scenarios. In addition, the dual optical properties also help to increase the difficulty of its imitation, ensuring the reliability and safety of the materials.

The host–guest doped strategy has become the main method of constructing optical materials due to its advantages such as low preparation cost, abundant sources of host‐guest, easy adjustment of performance and function.^[^ ^27^, ^28^, ^29^, ^30^, ^31^, ^32^, ^33^ ^]^ However, the tricky thing is that in the same doped system, photochromism and phosphorescence emission are in a competitive relationship.^[^ ^34^, ^35^, ^36^ ^]^ A rigid environment helps to suppress the molecular motion of the guests, thereby reducing the non‐radiative transitions of triplet excitons. On the contrary, photochromic molecules require a relaxed external environment during chemical reactions to avoid the hindrance of matrix effect on molecular conformational changes. Fortunately, recent work found that the host matrix with moderate rigidity can balance the external environment required for RTP and photochromism, thus combining the two originally mutually exclusive optical phenomena together.^[^ ^37^, ^38^ ^]^ **PC** polymer is not as rigid as polyvinyl alcohol, nor as flexible as polyethylene, have a moderate degree of rigidity.^[^ ^39^, ^40^, ^41^, ^42^ ^]^ Therefore, **PC**‐based doped materials possess both room temperature phosphorescence and photochromic properties. More excitingly, **PC** polymer as the host matrix has almost perfect universality. It can not only be used with dozens of phosphorescence guests to achieve phosphorescence emission from blue to red in the entire wavelength range, but also with various types of photochromic guests to achieve rapid reversible color changes from colorless to pink, red, and purple. What is even more shocking is that compared with the thirteen common polymers polyvinyl alcohol (**PVA**), polyvinyl pyrrolidone (**PVP**), polymethyl methacrylate (**PMMA**), polyvinyl butyral (**PVB**), etc., **PC** not only has the best universality, but also the **PC**‐based doped materials have the best phosphorescence or photochromic properties. Finally, we have successfully achieved large‐scale industrial preparation of doped film at the ton level, and the prepared doped film still has excellent optical properties, which can be directly used for product packaging. From an academic perspective, this work has discovered and demonstrated that **PC** is an excellent and overlooked host. From an industrial perspective, this work has developed optical functional materials with practical application value.

## 2 Results and Discussion

**Figure 1:**
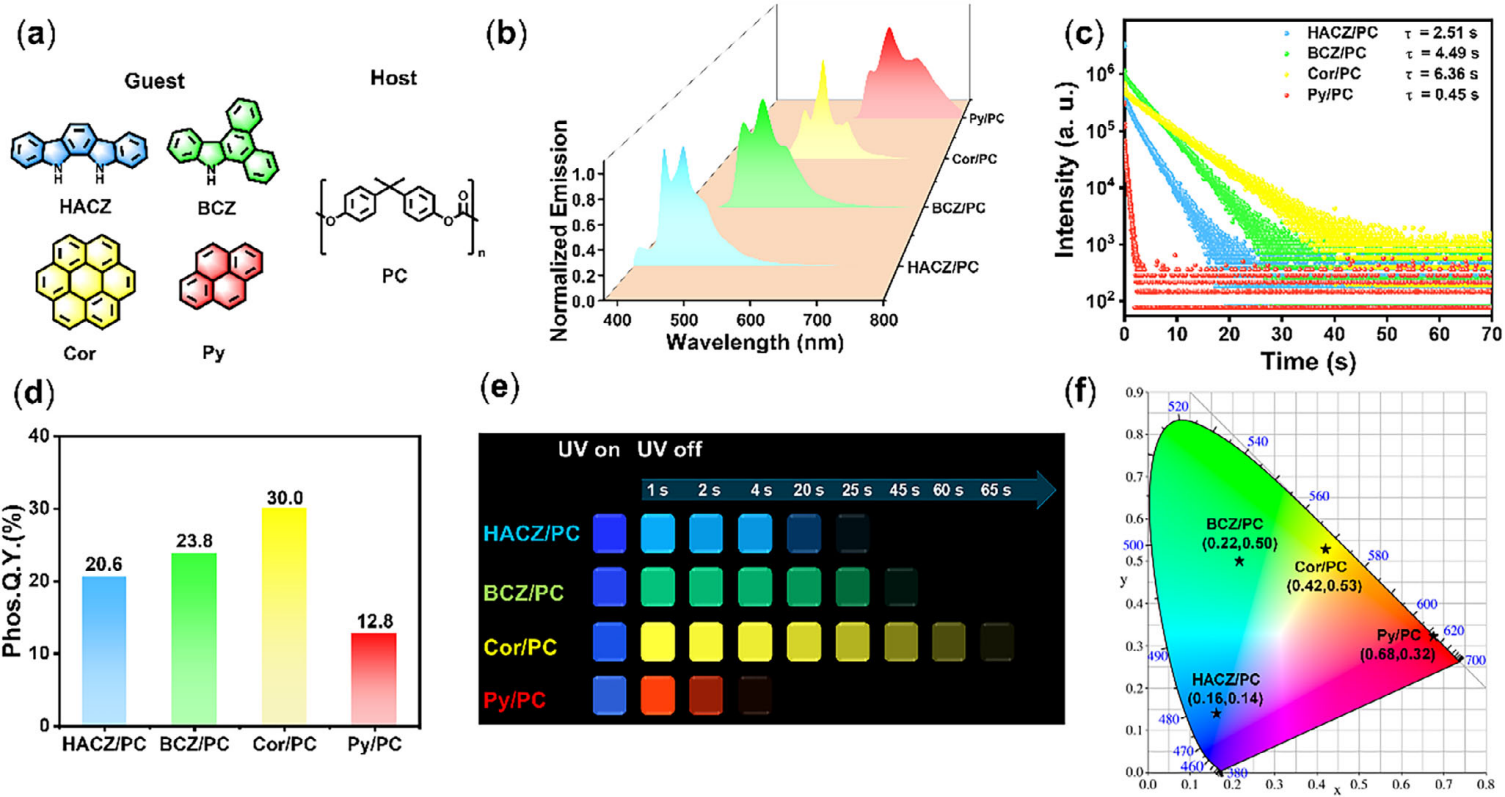
a) Molecular structures of the phosphorescence guest molecules and **PC** host matrix. b) Phosphorescence spectra of four doped materials **HACZ/PC** (E_x._ wavelength: 320 nm; delayed time: 0.1 ms), **BCZ/PC** (E_x._ wavelength: 380 nm; delayed time: 0.1 ms), **Cor/PC** (E_x._ wavelength: 360 nm; delayed time: 0.1 ms), and **Py/PC** (E_x._ wavelength: 360 nm; delayed time: 0.1 ms). c) Phosphorescence decay curves of four doped materials (E_x._ wavelength: 360 nm). d) Phosphorescence Q.Ys of four doped films. e) Luminescence photos of four doped materials. f) CIE coordinates of four doped materials.

In the doped system, the phosphorescence of the materials is essentially emitted by the guest molecules, so the luminescence performance of the guests are the key to determining the phosphorescence activity of the doped materials.^[^ ^43^, ^44^, ^45^, ^46^ ^]^ In order to construct a full‐color phosphorescence system, we selected four compounds 11,12‐dihydroindolo[2,3‐a]carbazole (**HACZ**), 9*H*‐dibenzo[a,c]carbazole (**BCZ**), coronene (**Cor**), and pyrene (**Py**) with blue, green, yellow, and red phosphorescence colors respectively as the guests (**Figure** **1**a). The molecular purities of four guests were confirmed using high‐performance liquid chromatography (Figure S1, Supporting Information). The phosphorescence wavelengths of the four guest molecules are 425/455 nm, 471/507 nm, 563 nm, and 609/635 nm, respectively (Figure S2, Supporting Information). It must be emphasized that we used the heat‐melting method to prepare doped materials, this method not only avoids the influence of residual organic solvent on the interaction between the host‐guest when using solvent method, but also conforms to the heat‐coating process of industrial production of **PC** polymer products. The TGA (thermogravimetric analysis) spectrum shown that the thermal decomposition temperature of PC polymer is about 354 °C (Figure S3, Supporting Information), and there is no phosphorescence activity at room temperature (Figure S4, Supporting Information). Given that the host‐guest doping ratio greatly influences the phosphorescence performance of doped system, the doped materials **BCZ/PC** with different guest‐to‐host molar ratios (1:50 to 1:10 000) were prepared, and the emission intensity reached the maximum at a guest‐to‐host molar ratio of 1:500 (Figure S5, Supporting Information). Therefore, the molar ratio of 1:500 was adopted for all doped materials. As expected, four doped films **HACZ/PC**, **BCZ/PC**, **Cor/PC**, and **Py/PC** exhibited bright and persistent blue, green, yellow, and red afterglow, respectively, with **Cor/PC** showing an afterglow time of up to 65 s (Figure 1e). The phosphorescence wavelength of the four doped materials are 449 nm, 481/514 nm, 566 nm, and 640 nm, respectively (Figure 1b), which are almost identical to that of the corresponding guests, indicating that the phosphorescence in this doped system is emitted by the guests. The delayed emission spectra of **BCZ/PC** at various temperatures from 77 to 293 K showed that the emission intensity gradually decreased as the temperature increased, demonstrating the afterglow was phosphorescence rather than thermally activated delayed fluorescence (Figure S6, Supporting Information). In addition, the CIE coordinates of four doped materials are (0.16, 0.14), (0.22, 0.50), (0.42, 0.53), and (0.68, 0.32), respectively (Figure 1f), which correspond to the afterglow colors of the doped materials. The phosphorescence lifetime of four doped materials is 2.51 s, 4.49 s, 6.36 s, and 0.45 s, respectively (Figure 1c). Moreover, the phosphorescence Q.Ys. of four doped films are 12.8%‐30.0% (Figure 1d), with **Cor/PC** having the highest phosphorescence quantum efficiency of 30%. The above results indicating that **PC** polymer is an excellent host matrix that can be used to construct phosphorescence materials with full emission wavelength, long lifetime, and high brightness. It should be pointed out that although the doped materials are prepared under air condition, the difference in phosphorescence performance between the two is very small compared to the doped materials prepared in a nitrogen atmosphere. Therefore, the influence of oxygen on the phosphorescence performance can be almost negligible (Figure S7, Supporting Information).

**Figure 2:**
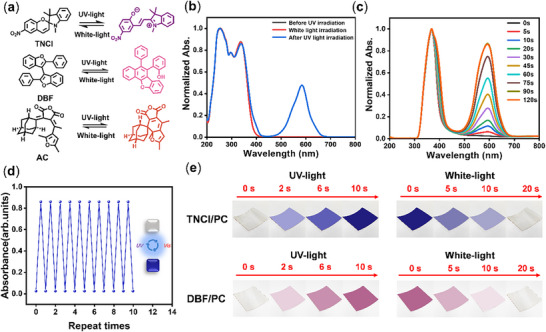
a) Molecular structures of the photochromic guest molecules. b) Photochromic property of guest **TNCI**. (Solvent: Tetrahydrofuran; Concentration: 1 × 10^−5^ mol L^−1^; UV‐light wavelength: 360 nm.) c) Time‐dependent absorption spectra change of original doped film **TNCI/PC** upon UV‐light irradiation. (UV‐light wavelength: 360 nm). d) Recycling of photochromic property of **TNCI/PC**. c) Appearance color change of doped films under different light irradiation.

In order to investigate the potential of **PC** polymer as the host matrix for constructing photochromic materials, three different types of photochromic molecules 1′,3′,3′‐Trimethyl‐6‐nitrospiro[chromene‐2,2′‐indoline] (**TNCI**), 2,2′‐diphenyl‐3,3′‐bibenzofuran (**DBF**), and aberchrome 670 (**AC**) were selected as the guests (**Figure** **2**a).^[^ ^47^, ^48^, ^49^ ^]^ After being exposed to UV‐light (360 nm, 10 W) for 3–5 s, three guests exhibited rapid and significant color changes, the colors changed from colorless to pink, deep red, and blue purple, respectively, and can all return to their original colorless state after being illuminated by white‐light (Figure 2b; Figure S8, Supporting Information). The UV‐absorption spectra shown that after being irradiated with UV‐light, a new absorption peak appeared at 500–550 nm for the guests, and this peak disappeared after being irradiated with white‐light (Figure 2b; Figure S8, Supporting Information). Correspondingly, three doped films **DBF/PC**, **AC/PC**, and **TNCI/PC** also exhibited excellent photochromic properties, the transparent and colorless films changed color to pink, deep red, and blue purple after being illuminated by a UV‐lamp, also showing a very rapid and obvious color changes (Figure 2e), and after being irradiated with white‐light for ≈ 20 s, all films also can fully recover to the initial state (Figure 2e). Similar to the guests themselves, the absorption spectra shown that after being irradiated with UV‐light for 5–60 s, the doped films exhibited a new and obvious absorption band with a peak of about 500–550 nm (Figure 2c; Figure S9, Supporting Information). Moreover, even after 10 consecutive change‐recovery cycles, no significant fatigue was observed in the doped material (Figure 2d), meaning that the photochromic effect of the **PC**‐based doped films shown excellent reversibility and stability. The above results fully demonstrate that **PC** polymer is also an excellent host matrix for constructing doped materials with photochromic properties.

**Figure 3:**
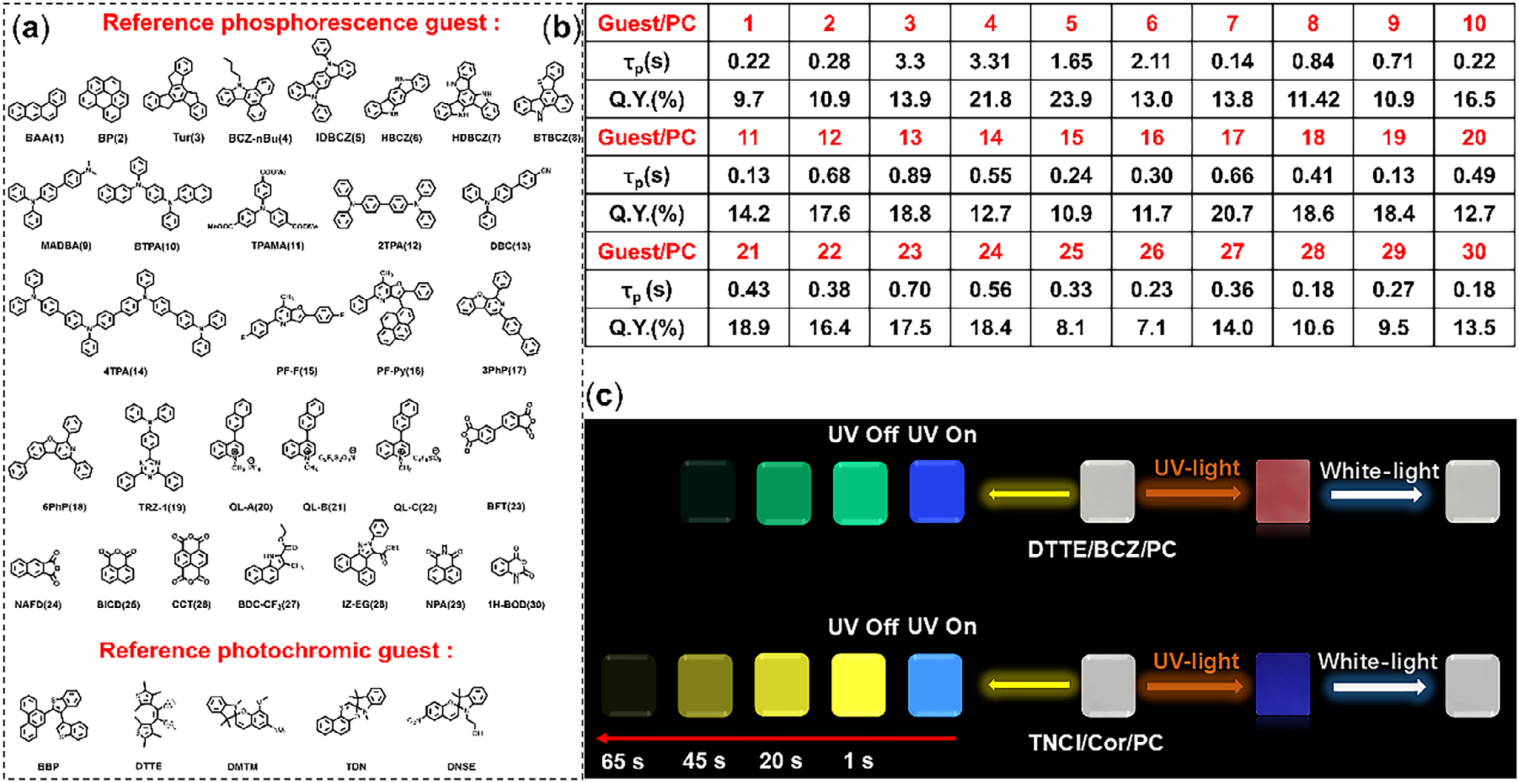
a) Molecular structures of the reference phosphorescence guest molecules and reference photochromic guest molecules. b) Phosphorescence lifetime and phosphorescence quantum efficiency of the reference doped materials. c) Luminescence photos and appearance color change photos of the reference doped materials.

An important characteristic of an excellent host matrix is its good universality. To verify whether the **PC** host possesses this feature, we first selected twenty‐eight luminescent molecules as reference phosphorescent guests to construct the doped materials (**Figure** **3**a). The molecular structure types of the twenty‐eight guest molecules are different, and their phosphorescence wavelengths (77 K) range from 450 to 650 nm (Figures S10 and S11, Supporting Information). Therefore, the selected guests have strong representativeness. The results are very exciting, as all doped materials exhibited RTP activity, these materials emitted colorful afterglows such as blue, cyan, green, yellow, orange, and red (Figure 3c; Figure S12, Supporting Information), and the phosphorescence wavelength ranges from 450 to 650 nm (Figures S13 and S14, Supporting Information). The phosphorescence lifetime of all materials can exceed 100 ms, with a maximum of 3.31s (Figure 3b; Figures S15 and S16, Supporting Information), the phosphorescence quantum efficiencies are 7.1% – 23.0% (Figure 3b). The above results clearly demonstrated that **PC** polymer is an excellent host matrix for constructing host‐guest doped phosphorescence materials, with very excellent universality. Next, a series of photochromic molecules were selected as reference photochromic guests to construct doped materials (Figure 3a). The results are equally exciting. After being light‐illuminated for a few seconds, the doped materials can quickly change color from colorless to pink or red or purple, and can also return to their initial state after being illuminated by white‐light (Figure 3c; Figure S17, Supporting Information). The absorption spectra also shown that after being UV‐light illuminated, the doped films exhibited new absorption peaks in the wavelength region of 450–500 nm (Figure S18, Supporting Information). These results also meaning that **PC** polymer as the host matrix for constructing photochromic materials has excellent universality. The combination of phosphorescence activity and photochromic properties is of great significance for enhancing the economic value of polymer films. to investigate the compatibility of these two properties in **PC** polymer, five three‐component doped materials were prepared (**DTTE/BCZ/PC**, **TNCI/HACZ/PC**, **TNCI/BCZ/PC**, **TNCI/Cor/PC**, **TNCI/Py/PC**). The results displayed that all doped materials possessed both RTP activity and photochromic properties. The films still have green or yellow afterglow, phosphorescence lifetime of 0.42 s–6.30 s (Figure S19, Supporting Information), phosphorescence efficiency of 12.5% – 29% (Figure S20, Supporting Information), after UV‐light irradiation, the appearance colors can also quickly and significantly change from colorless to red or purple (Figure S21, Supporting Information), accompanied by the appearance of new absorption peaks (Figure S22, Supporting Information). Compared with the phosphorescence activity or photochromism of the corresponding two‐component doped materials, that of in the three‐component materials have almost no changed, indicating that the two properties can not only coexist perfectly in **PC** polymer, but also have almost no influence on each other. In addition, the duration of light radiation has almost no effect on the phosphorescence intensity of the three‐components doped materials, and the results shown that continuous irradiation of the doped materials for 120 s results in almost no change in the phosphorescence emission (Figure S23, Supporting Information).

**Figure 4:**
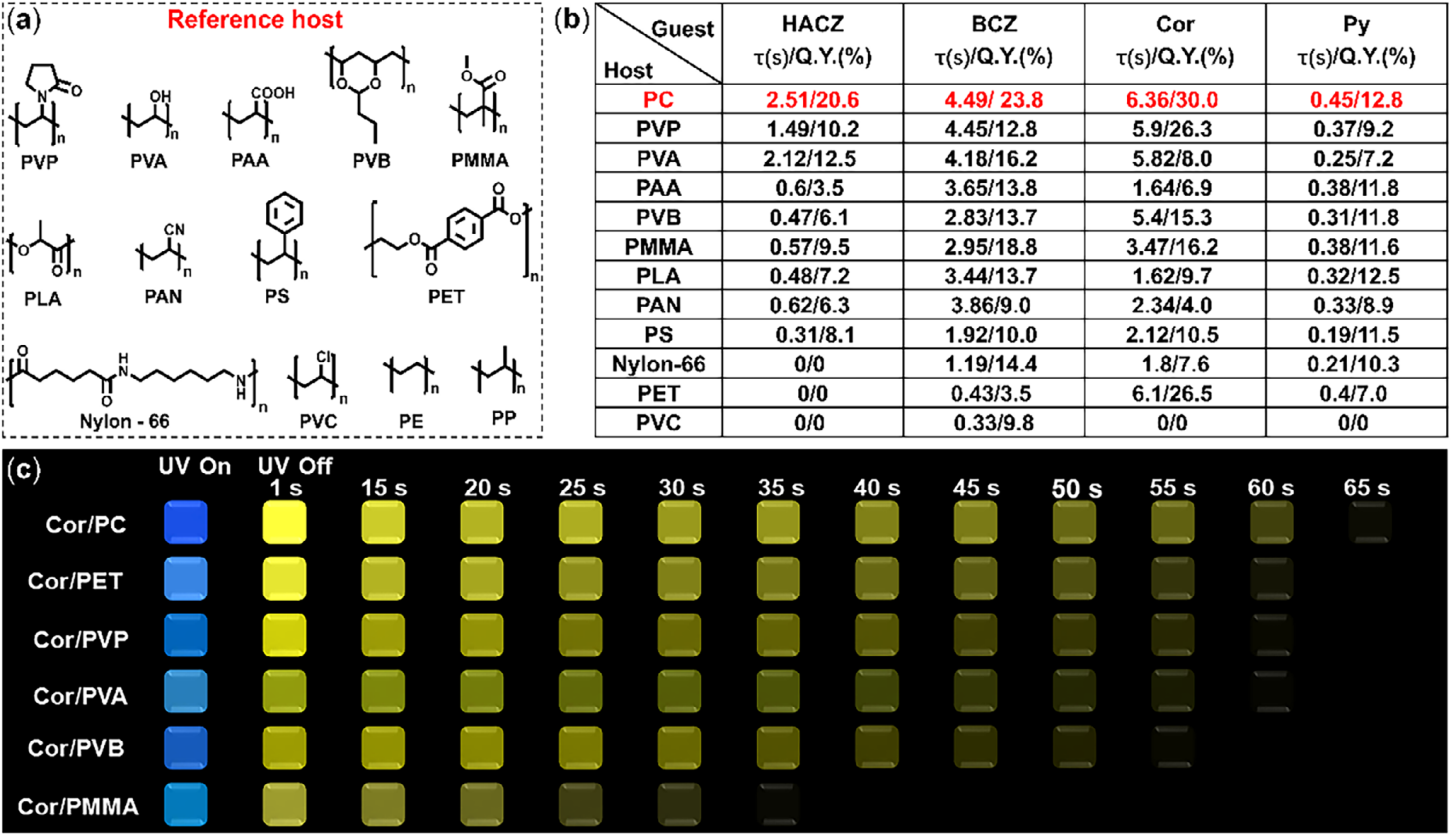
a) Molecular structures of the reference host polymers. b) Phosphorescence lifetime and phosphorescence Q.Ys. of doped materials. c) Luminescence photos of the doped films.

To further validate the superiority of **PC** polymer as the host matrix, we selected thirteen commonly used polymers reported in the literatures, including polyethylene (**PE**), polypropylene (**PP**), polyvinyl chloride (**PVC**), polystyrene (**PS**), polyvinyl pyrrolidone (**PVP**), polyacrylic acid (**PAA**), polymethyl methacrylate (**PMMA**), polyvinyl alcohol (**PVA**), polyacrylonitrile (**PAN**), polyvinyl butyral (**PVB**), polylactic acid (**PLA**), polyethylene terephthalate (**PET**), and polyhexamethylene adipamide (**Nylon‐66**) as the reference hosts (**Figure** **4**a). The absorption wavelength of the reference polymers is about 250–350 nm (Figure S24, Supporting Information), and none of them have RTP activity (Figure S25, Supporting Information). Continue to select the four compounds **HACZ**, **BCZ**, **Cor**, and **Py** as the guests to construct a large number of doped materials for comparison. The results displayed that the doped materials based on polymers **PE**, **PP**, and **PVC** have almost no RTP activity (Figure S26, Supporting Information). In the **PET** and **Nylon‐66** based doped system, the materials with **BCZ**, **Cor**, and **Py** as the guest have RTP activity (Figure S27, Supporting Information), the phosphorescence Q.Ys. are 3.5%‐26.5% (Figure 4b), and the lifetime is 0.21–6.1 s (Figure 4b; Figure S28, Supporting Information). For the doped materials based on the other eight polymers as the host, although they all have RTP activity (Figure S29, Supporting Information), their phosphorescence performance is still weaker than that of the doped materials **PC**‐based doped materials. Specifically, the phosphorescence performance of the **PLA**, **PAN**, and **PS**‐based doped materials is relatively the weakest, with phosphorescence Q.Ys. of 4.0%–13.7% and phosphorescence lifetime of 0.19 s–3.86 s (Figure 4b; Figure S30, Supporting Information). For the commonly polymer hosts **PVP**, **PVA**, **PMMA**, **PVB**, and **PAA**. Although the doped materials based on them also have good phosphorescence properties (Figure S31, Supporting Information), with phosphorescence lifetimes ranging from 0.25 s to 5.9 s (Figure 4b; Figure S32, Supporting Information), and phosphorescence Q.Ys. of 3.5% – 26.3% (Figure 4b), but the phosphorescence performance is still weaker than the corresponding **PC**‐based doped materials. Considering that **PVA** and **PVP** are currently the two most commonly used polymer hosts in literature reports,^[^ ^50^, ^51^ ^]^ to further compare the superiority of **PVA, PVP** and **PC** polymers as the host, we further prepared doped materials by combining all thirty control guests with **PVA** and **PVP** polymers. For the **PVP**‐based doped materials, only three materials have better phosphorescence performance than **PC**‐based materials, while the phosphorescence Q.Y. and phosphorescence lifetime of the remaining twenty‐seven doped materials are weaker than those of **PC**‐based materials, and four of them have almost no phosphorescence activity (Figures S33–S35; Table S1, Supporting Information). For **PVA**‐based doped materials, five doped materials with N‐H groups as guests have phosphorescence properties that are better than those of **PC**‐based doped materials, while the phosphorescence activity of the remaining twenty‐five materials can be ignored or very weaker than that of **PC**‐based materials (Figures S36–S38; Table S2, Supporting Information). This was because **PVA** polymer containing hydroxyl groups form hydrogen bonds with the guests containing N─H groups, resulting in excellent phosphorescence properties of the doped materials. The above massive data fully confirmed that **PC** polymer is the optimal host matrix for constructing doped materials among the currently known polymer hosts, especially when paired with guests that do not contain polar groups. We further investigated the potential of thirteen reference polymers as the host for constructing photochromic materials, continue to choose **DBF**, **AC**, and **TNCI** as photochromic guest molecules. As expected, the doped materials constructed with four flexible polymers **PE**, **PP**, **PVC**, and **PET** as the host exhibited excellent photochromic phenomena (Figures S39 and S40, Supporting Information). However, for the doped materials with eight other polymers as the host matrix, only the materials with **TNCI** as the guest have color change when exposed to UV‐light (Figures S41–S43, Supporting Information), which due to the highly sensitive photochemical activity of **TNCI** compound. In summary, **PC** polymer is excellent candidates for constructing phosphorescence and photochromic materials, especially with significant advantages in the construction of phosphorescence materials.

**Figure 5:**
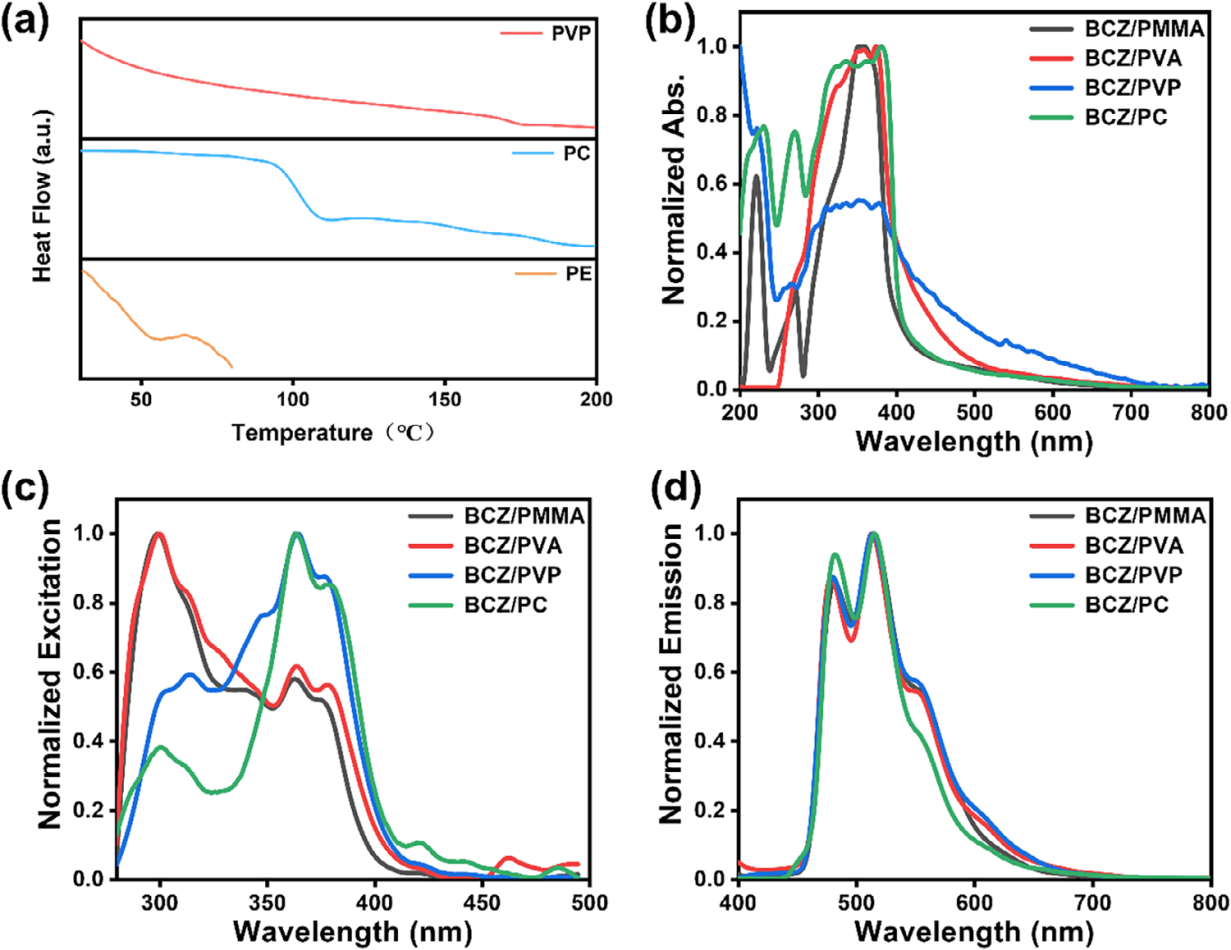
a) DSC spectra of the hosts **PVP**, **PC**, and **PE**. b) Absorption spectra of the doped materials. c) Excitation spectra of phosphorescence emission from doped materials. d) Phosphorescence emission spectra of the doped materials (E_x._ wavelength: 380 nm; Delayed time: 0.1 ms).

Our previous work confirmed that if doped materials want to have both excellent room temperature phosphorescence and photochromic properties, the host needs to have a moderate degree of rigidity.^[^ ^37^ ^]^ While suppressing non‐radiative transitions of excitons, it is also necessary to have a certain degree of spatial elasticity to accommodate changes in molecular configuration. Differential scanning calorimetry (DSC) spectra shown that **PC** has a moderate glass transition temperature (T*_g_*), lower than rigid polymers such as **PVP** and **PMMA**, and higher than flexible hosts such as **PP** and **PE** (**Figure** **5**a; Figure S44, Supporting Information), indicating that **PC** polymer has a moderate degree of rigidity. To reveal as much as possible the reason why **PC** polymer as the host is superior to other polymers, the absorption spectra and excitation spectra of four doped materials (**BCZ/PC**, **BCZ/PVA**, **BCZ/PVP**, **BCZ/PMMA**) were further tested. The results shown that the six doped materials had highly consistent absorption, emission, and excitation wavelengths (Figure 5b–d), which meaning that the energy transfer between the host and guest is not the reason why **PC** host is superior to other polymer hosts. In the doped system, the host matrix first needs to have a certain degree of deformability to encapsulate and accommodate guest molecules, and then there should be strong intermolecular interactions between the guest and host to suppress the molecular motion of the guest. For **PC** polymer, the structural units consist of two benzene rings connected by isopropyl group. The presence of isopropyl groups gives the polymer a certain degree of flexibility, allowing it to undergo certain deformations to wrap the guest molecules, while the two benzene rings can form π – π interactions with the guest molecules to suppress their molecular motion.^[^ ^52^, ^53^ ^]^ In addition, the flexible side of polymer facilitates the conformational changes required for guest molecules to undergo photo reactions, thus endowing doped materials with excellent photochromic properties.

**Figure 6:**
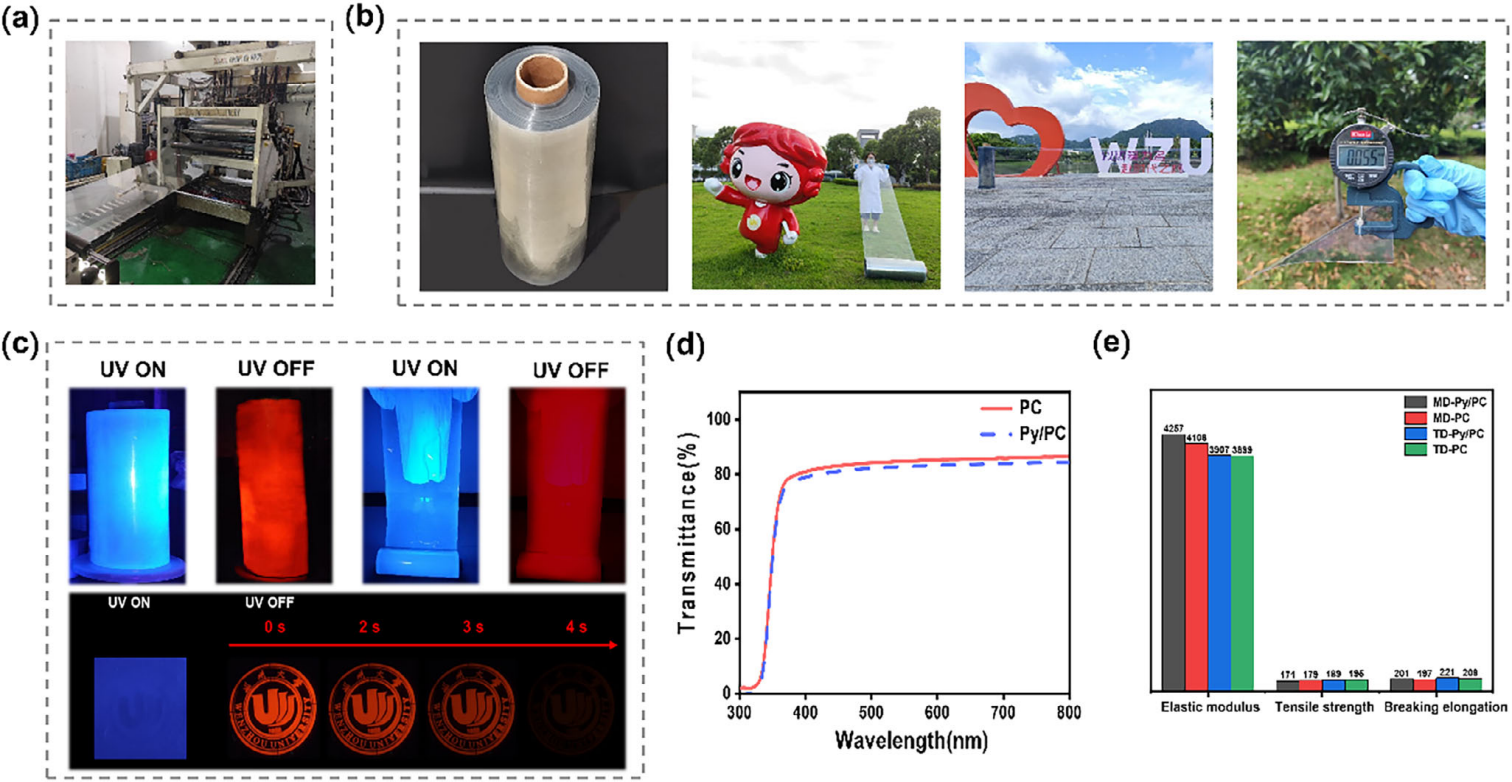
a) Preparation process of the film **Py/PC**. b) Size and shape of the film **Py/PC**. c) Photophysical luminescence phenomenon of the film **Py/PC**. d) Transmittance of pure **PC** film and **Py/PC** film. e) Mechanical properties of pure **PC** film and **Py/PC** film. Elastic modulus as Young's modulus, is a fundamental mechanical property that quantifies a material's stiffness or resistance to elastic deformation under applied stress, units of elastic modulus is the MPa. Tensile strength referred to as ultimate tensile strength, is a critical mechanical property that quantifies the maximum stress a material can withstand while being stretched or pulled before necking or fracture occurs, units of tensile strength is the MPa. Breaking elongation is a critical mechanical property that quantifies the maximum tensile deformation a material can undergo before rupture, expressed as the percentage increase in length relative to its original gauge length, units of breaking elongation is the percentage (%).

**PC** film was widely used in various packaging applications, therefore, **PC** film endowed with optical properties had extremely high industrial value in areas such as anti‐counterfeiting of items. However, the film required industrial grade large‐scale preparation to meet the large‐area and low‐thickness required for actual production. Here, mixed 1000 kg of PC polymer slices and 1000 g of the guest Py evenly, then direct prepared the film in the cast‐coating extrusion machine with a set temperature of 220 °C at ambient environment. (**Figure** **6**a). The prepared films have a width of 40 cm and a thickness of ≈50–60 µm, with each roll length exceeding 500 m (Figure 6b), and the doped films exhibited good uniformity and transparency (Figure 6b). In addition, the UV–vis transmittance and the mechanical properties of the doped films were comparable to those of the pure **PC** film (Figure 6d,e). These results meaning that the addition of the guest did not alter the original physical properties of the polymer. Most importantly, after being irradiated with UV‐light, the doped films can clearly display a bright red afterglow of ≈ 3–5 s (Figure 6c), with a phosphorescence lifetime of 0.43 s and a phosphorescence Q.Y. of 11.8% (Figure S45, Supporting Information), which was almost consistent with the performance of the doped film prepared at small doses in the laboratory, fully demonstrating the stability of amplification experiment. The prepared doped films can be directly used for commercial packaging of various items. By utilizing the optical properties of the films, it is can to easily and effectively verify products, demonstrating excellent anti‐counterfeiting effects and ultimately greatly improving the economic value of **PC** film.

## 3 Conclusion

In this work, we used **PC**, one of the most widely used polymers in human life as the host matrix, thirty‐four luminescent molecules as phosphorescence guests, and eight photochromic compounds as photochromic guests to construct high‐performance doped materials. **PC**‐based doped materials possessed both room temperature phosphorescence activity and photochromic properties. Phosphorescence can achieve full band emission from blue to green to yellow to red, with the phosphorescence lifetime of 0.45–6.36 s and the phosphorescence Q.Y. of 12.8% ‐30%. Doped materials can achieve rapid, sensitive, and reversible transitions from colorless to pink, red, or blue purple under light‐illumination. What is even more surprising was that **PC** polymer as the host matrix not only has good universality, but also has significant advantages in phosphorescence performance compared to doped materials based on more than thirteen common host polymers such as **PVA** and **PMMA**. This work had discovered and proven that **PC** polymer was a guest that has long been overlooked by researchers, but has strong universality and excellent performance. Finally, we achieved large‐scale industrial preparation of doped film at the ton level, and the prepared doped film still has excellent optical properties, which can be directly used for product packaging.

## Conflict of Interest

The authors declare no conflict of interest.

## Supporting information



Supporting Information

## Data Availability

The data that support the findings of this study are available from the corresponding author upon reasonable request.
