# Supplementary material for: Ton‐Scale Industrial Optical Polycarbonate Film: Combining Full‐Color Phosphorescence and Various Photochromic
Source: Adv Sci (Weinh). 2025 Sep 30;12(48):e17170. doi: 10.1002/advs.202517170 (PMC12752636; doi:10.1002/advs.202517170)
Supplement: Supplementary file 1 — Supporting Information [file ADVS-12-e17170-s001.pdf]

Ton-scale Industrial Optical Polycarbonate Film: Combining Full-color Phosphorescence and Various Photochromic

Peng Chen[1], Jielei Jin[1], Yanke Zhu[2], Yunxiang Lei[1,3]*, Xinyang Ye[1], Wenbo Dai[1,3]*, Chuangjie Gu[2], Miaochang Liu[1], Xiaobo Huang[1]*, Dan Wang[2]*

[1] *School of Chemistry and Materials Engineering, Wenzhou University, Wenzhou 325035, P. R. China*

[2] *Department of Pediatrics, The First Affiliated Hospital of Wenzhou Medical University, Wenzhou 325035, China*

[3] *Key Lab of Biohealth Materials and Chemistry of Wenzhou, Wenzhou 325035, P. R. China*

1. Experimental

1.1 Measurements and materials

^{1}H and ^{13}C NMR spectra were carried out by a Bruker ARX500 spectrometer with $CDCl_3$ as the solvent. UV-vis absorption spectra were measured by a Persee TU-1901 spectroscopy. Fluorescence spectra were measured by a Hitachi F-7000 spectrophotometer. Phosphorescence spectra were measured by a FLS920 lifetime and steady state spectrometer. X-Ray crystal structure analyses were conducted on a Bruker-AXS SMART APEX2 CCD diffractometer. Solid-state emission quantum yields were collected on a FluoroMax-4 (Horiba Jobin Yvon) fluorimeter equipped with integrated sphere. The Tg values of host films were measured by differential scanning calorimetry (DSC) using Mettler DSC 3+ with a heating rate of 10 K min^{-1} under nitrogen. All host polymers were all commercially purchased without further processing. For the guest compounds, twenty guest compounds were commercially purchased and purified, while fourteen guest compounds were synthesized using methods in reported literatures. All polymers were all commercially purchased without further processing, the molecular weight of PC is 20500 g/mol. The molecular weights of the thirteen reference polymers (polyethylene, polypropylene, polyvinyl chloride, polystyrene, polyvinyl pyrrolidone, polyacrylic acid, polymethyl methacrylate, polyvinyl alcohol, polyacrylonitrile, polyvinyl butyral, polylactic acid, polyethylene terephthalate, and polyhexamethylene adipamide) are 40000 g/mol, 80000 g/mol, 50000 g/mol, 65000 g/mol, 58000 g/mol, 450000 g/mol, 450000 g/mol, 20000 g/mol, 25000 g/mol and 20000 g/mol, respectively.

The solid-state emission quantum yields were collected by using FluoroMax-4 (Horiba Jobin Yvon) fluorimeter equipped with integrated sphere, the test result is the absolute luminescence quantum efficiency, which is the ratio of the collected emission spectrum data to the basic absorption spectrum data. When measuring the phosphorescence quantum yield, the emission spectra of the materials were collected with a delay time of 0.1 ms to filter the fluorescence emission.

1.2 Synthetic routes of guests

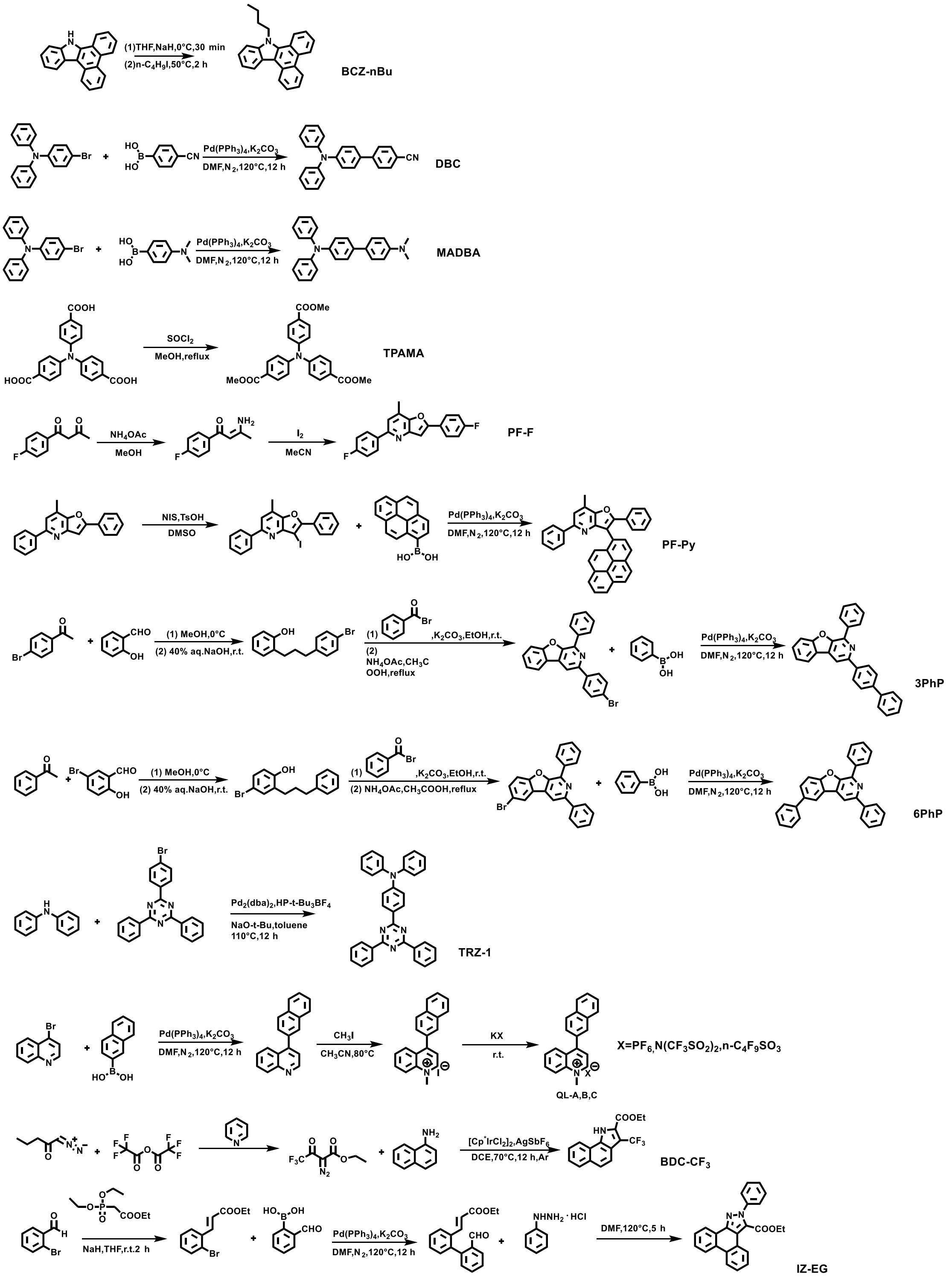

Scheme S1. Synthetic procedures of guest molecules

1.3 Preparation of polymer doped materials

After the guest compound and the host polymer are physically mixed, the vacuum is dried for eight hours, and then poured into a flat vulcanizing machine or a film blowing machine to make a film. The doped materials with high guest-host mass ratio (1:50, 1:100, 1:200, 1:500) are using direct weighing method, while for low guest-host molar ratio (1:1000, 1:5000, 1:10000) doped materials, we use the indirect dilution method.

Preparation of **PC**-based doped materials: The PC and guest molecule powders were thoroughly mixed and placed on a glass slide. The mixture was heated to 220°C on a hot plate until it reached a molten state. The molten mixture was then stirred for 2 minutes. Another glass slide was placed on top to press the mixture into a 1 mm-thick film. Finally, the sample was cooled to room temperature.

Preparation of **PVP**-based doped materials: Dissolve 5 g of **PVP** powder in 100 mL of dichloromethane (DCM) to prepare a 50 mg/mL host solution, then dissolve 10 mg of the guest molecule in 10 mL of tetrahydrofuran (THF) to obtain a 1 mg/mL guest solution. Mix uniformly at the specified ratio, then dry in a vacuum oven at 40°C.

Preparation of **PVA**-based doped materials: Dissolve 5 g of **PVA** powder in 100 mL of H_2O to prepare a 50 mg/mL host solution, then dissolve 10 mg of the guest molecule in 10 mL of tetrahydrofuran (THF) to obtain a 1 mg/mL guest solution. Mix uniformly at the specified ratio, then dry in a vacuum oven at 100°C.

Preparation of **PAA**-based doped materials: Dissolve 5 g of **PAA** powder in 100 mL of C_2H_5OH to prepare a 50 mg/mL host solution, then dissolve 10 mg of the guest molecule in 10 mL of tetrahydrofuran (THF) to obtain a 1 mg/mL guest solution. Mix uniformly at the specified ratio, then dry in a vacuum oven at 80°C.

Preparation of **PVB**-based doped materials: Dissolve 5 g of **PVB** powder in 100 mL of dichloromethane (DCM) to prepare a 50 mg/mL host solution, then dissolve 10 mg of the guest molecule in 10 mL of tetrahydrofuran (THF) to obtain a 1 mg/mL guest solution. Mix uniformly at the specified ratio, then dry in a vacuum oven at 40°C.

Preparation of **PMMA**-based doped materials: Dissolve 5 g of **PMMA** granules in 100 mL of dichloromethane (DCM) to prepare a 50 mg/mL host solution, then dissolve 10 mg of the guest molecule in 10 mL of tetrahydrofuran (THF) to obtain a 1 mg/mL guest solution. Mix uniformly at the specified ratio, then dry in a vacuum oven at 40°C.

Preparation of **PLA**-based doped materials: Dissolve 5 g of **PLA** granules in 100 mL of dichloromethane (DCM) to prepare a 50 mg/mL host solution, then dissolve 10 mg of the guest molecule in 10 mL of tetrahydrofuran (THF) to obtain a 1 mg/mL guest solution. Mix uniformly at the specified ratio, then dry in a vacuum oven at 40°C.

Preparation of **PAN**-based doped materials: Dissolve 5 g of **PAN** powder in 100 mL of N,N-Dimethylformamide (DMF) to prepare a 50 mg/mL host solution, then dissolve 10 mg of the guest molecule in 10 mL of tetrahydrofuran (THF) to obtain a 1 mg/mL guest

solution. Mix uniformly at the specified ratio, then dry in a vacuum oven at 150°C.

Preparation of **PS**-based doped materials: Dissolve 5 g of **PS** granules in 100 mL of dichloromethane (DCM) to prepare a 50 mg/mL host solution, then dissolve 10 mg of the guest molecule in 10 mL of tetrahydrofuran (THF) to obtain a 1 mg/mL guest solution. Mix uniformly at the specified ratio, then dry in a vacuum oven at 40°C.

Preparation of **PVC**-based doped materials: Dissolve 5 g of **PVC** powder in 100 mL of tetrahydrofuran (THF) to prepare a 50 mg/mL host solution, then dissolve 10 mg of the guest molecule in 10 mL of tetrahydrofuran (THF) to obtain a 1 mg/mL guest solution. Mix uniformly at the specified ratio, then dry in a vacuum oven at 60°C.

Preparation of **Nylon-66**-based doped materials: Dissolve 5 g of **Nylon-66** granules in 100 mL of Methanoic acid (HCOOH) to prepare a 50 mg/mL host solution, then dissolve 10 mg of the guest molecule in 10 mL of tetrahydrofuran (THF) to obtain a 1 mg/mL guest solution. Mix uniformly at the specified ratio, then dry in a vacuum oven at 100°C.

Preparation of **PET**-based doped materials: The **PET** and guest molecule powders were thoroughly mixed and placed on a glass slide. The mixture was heated to 200°C on a hot plate until it reached a molten state. The molten mixture was then stirred for 2 minutes. Another glass slide was placed on top to press the mixture into a 1 mm-thick film. Finally, the sample was cooled to room temperature.

Preparation of **PE**-based doped materials: The **PE** and guest molecule powders were thoroughly mixed and placed on a glass slide. The mixture was heated to 150°C on a hot plate until it reached a molten state. The molten mixture was then stirred for 2 minutes. Another glass slide was placed on top to press the mixture into a 1 mm-thick film. Finally, the sample was cooled to room temperature.

Preparation of **PP**-based doped materials: The **PP** and guest molecule powders were thoroughly mixed and placed on a glass slide. The mixture was heated to 150°C on a hot plate until it reached a molten state. The molten mixture was then stirred for 2 minutes. Another glass slide was placed on top to press the mixture into a 1 mm-thick film. Finally, the sample was cooled to room temperature.

2. Figures and tables

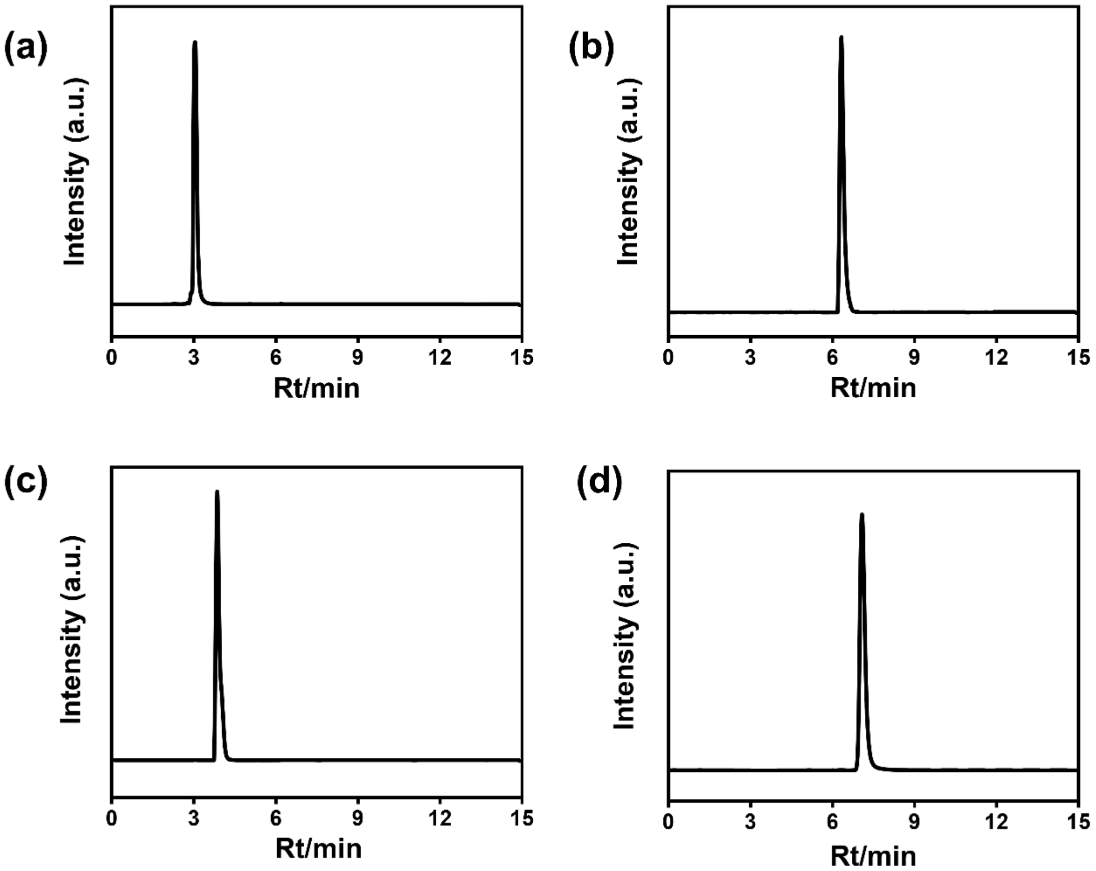

Figure S1. High performance liquid chromatography of guests **HACZ** (a), **BCZ** (b), **Cor**(c), and **Py** (d). Conditions: EtOH/H_2O = 70%: 30%.

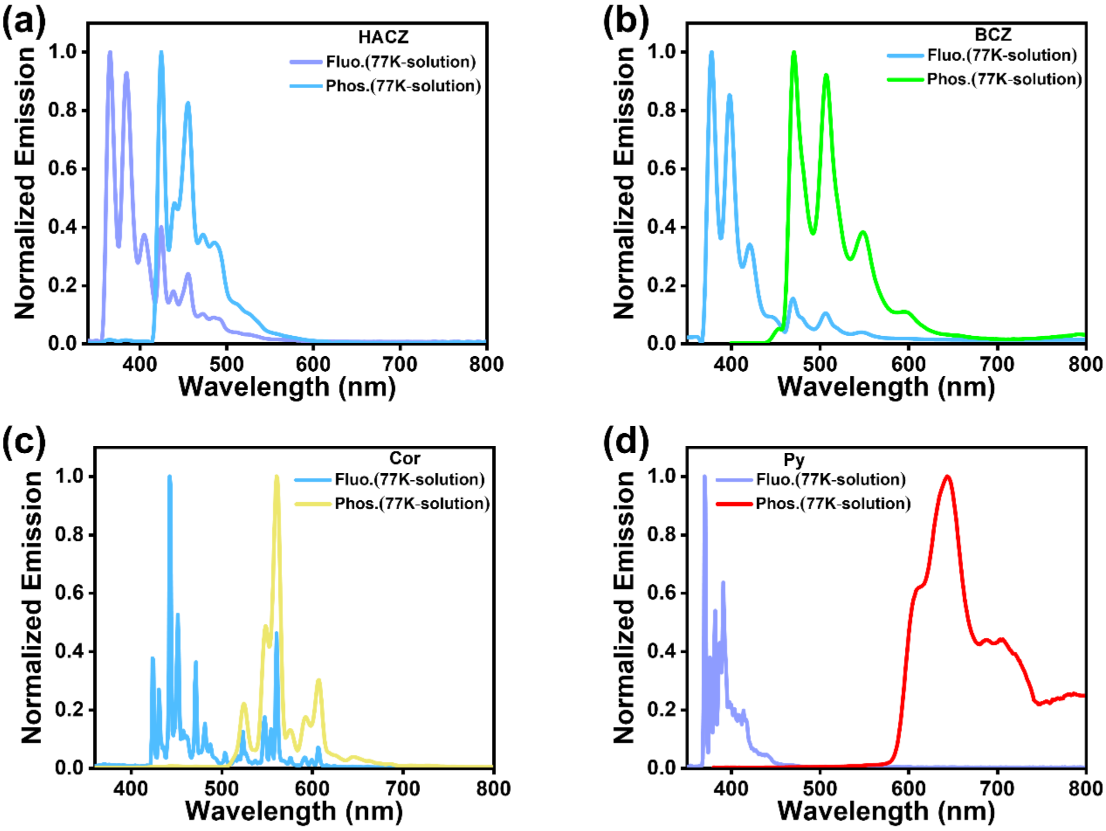

Figure S2. (a) Emission spectra of **HACZ** at 77 K (Ex. of fluo.: 320 nm; Ex. of phos.: 320 nm, delayed time: 0.1 ms). (b) Emission spectra of **BCZ** at 77 K (Ex. of fluo.: 330 nm; Ex. of phos.: 360 nm, delayed time: 0.1 ms). (c) Emission spectra of **Cor** at 77 K (Ex. of fluo.: 340 nm; Ex. of phos.: 360 nm, delayed time: 0.1 ms). (d) Emission spectra of **Py** at 77 K (Ex. of fluo.: 340 nm; Ex. of phos.: 360 nm, delayed time: 0.1 ms).

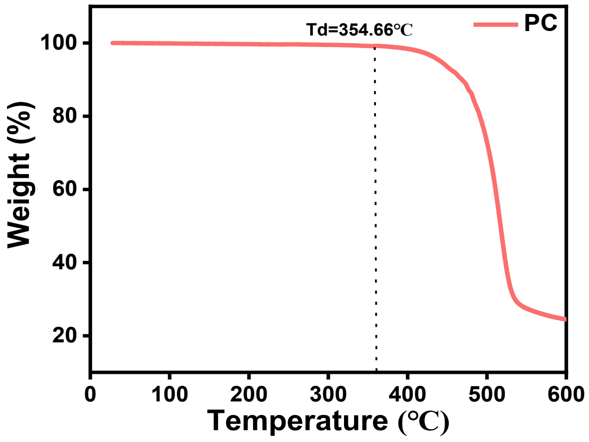

Figure S3. TGA spectrum of **PC** polymer.

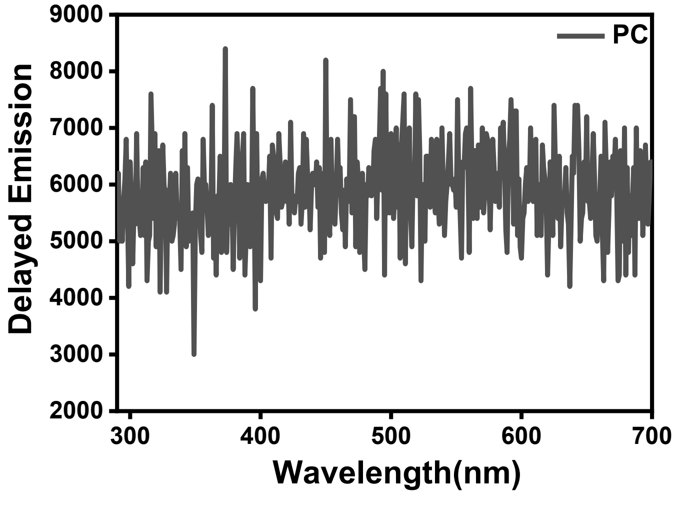

Figure S4. Phosphorescence spectrum of **PC** polymer (Ex. 290 nm; delayed time: 0.1 ms).

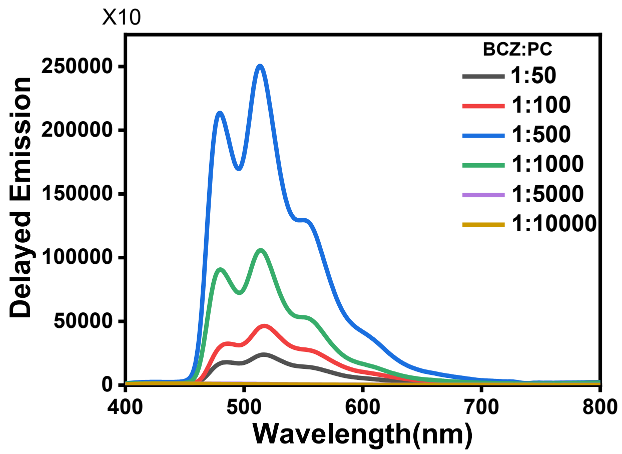

Figure S5. Delayed emission spectra of the **BCZ/PC** doped materials with different amounts of **BCZ** (Ex.: 380 nm, delayed time: 0.1 ms).

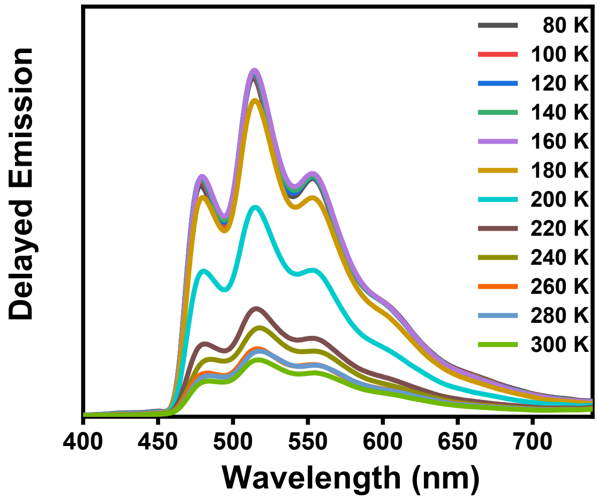

Figure S6. Delayed emission spectra of **BCZ/PC** under different temperatures (Ex.: 380 nm; Delayed time: 0.1 ms).

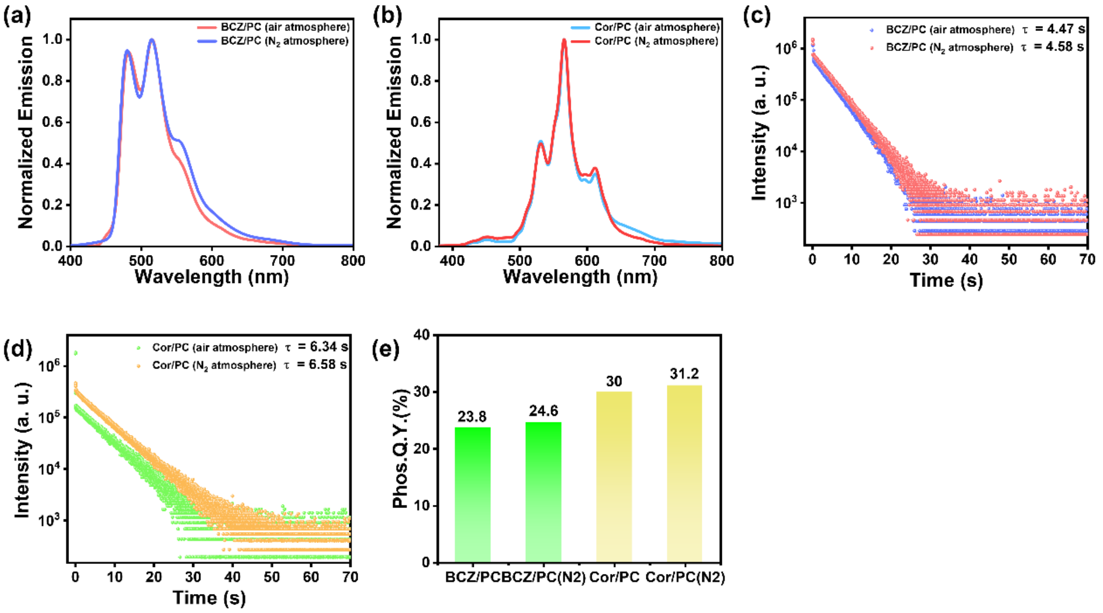

Figure S7. (a) Phosphorescence spectra of two doped materials **BCZ/PC** (Ex. 380 nm; delayed time: 0.1 ms). (b) Phosphorescence spectra of two doped materials **Cor/PC** (Ex. 360 nm; delayed time: 0.1 ms). (c) Phosphorescence decay curves of two doped materials **BCZ/PC** (Ex. 380 nm). (d) Phosphorescence decay curves of two doped materials **Cor/PC** (Ex. 360 nm). (e) Phosphorescence Q.Ys. of four doped materials.

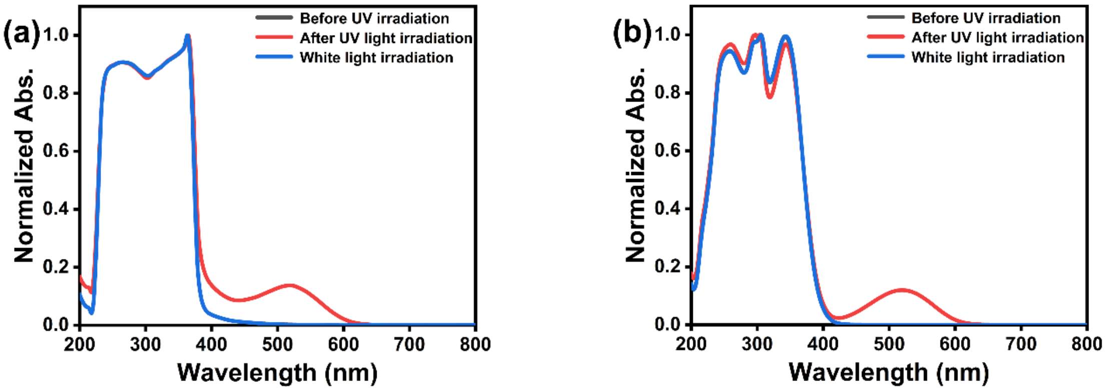

Figure S8 (a) Photochromic property of guest **DBF**. (Solvent: Tetrahydrofuran; Concentration: 1 × 10^{-5} mol/L; UV light wavelength: 360 nm.) (b) Photochromic property of guest **AC**. (Solvent: Tetrahydrofuran; Concentration: 1 × 10^{-5} mol/L; UV light wavelength: 360 nm.)

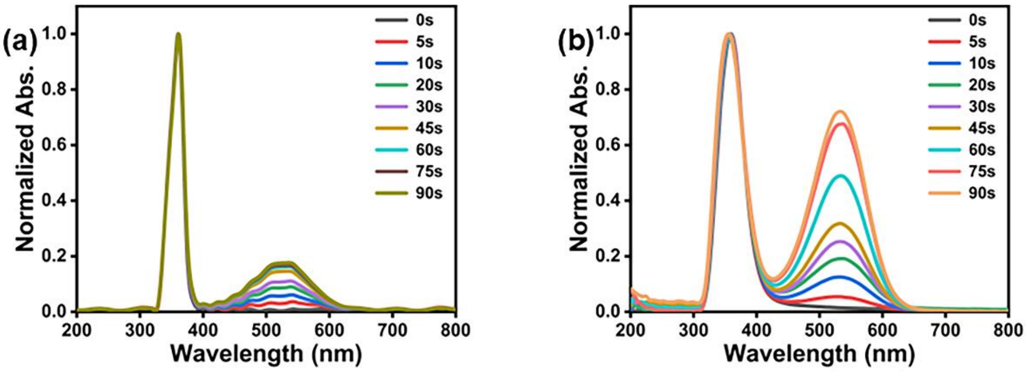

Figure S9. Time-dependent absorption spectra changes of **DBF/PC** (a) and **AC/PC** (b) original-film upon UV-light irradiation with increasing time. (UV light wavelength: 360 nm.)

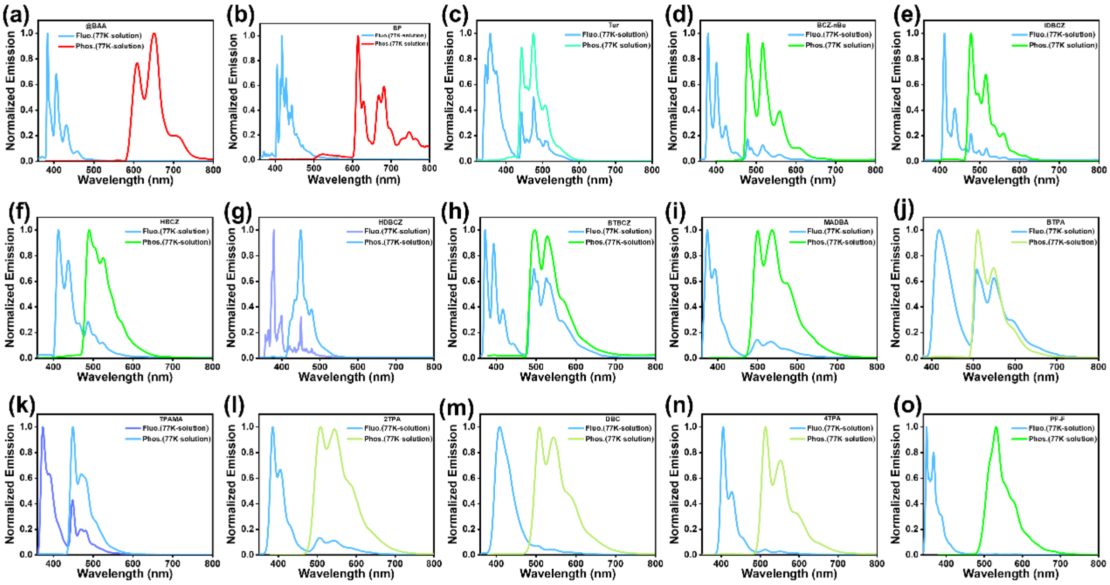

Figure S10. Emission spectra of guests at 77 K (Ex. of fluo.: 340 nm; Ex. of phos.: 360 nm, delayed time: 0.1 ms).

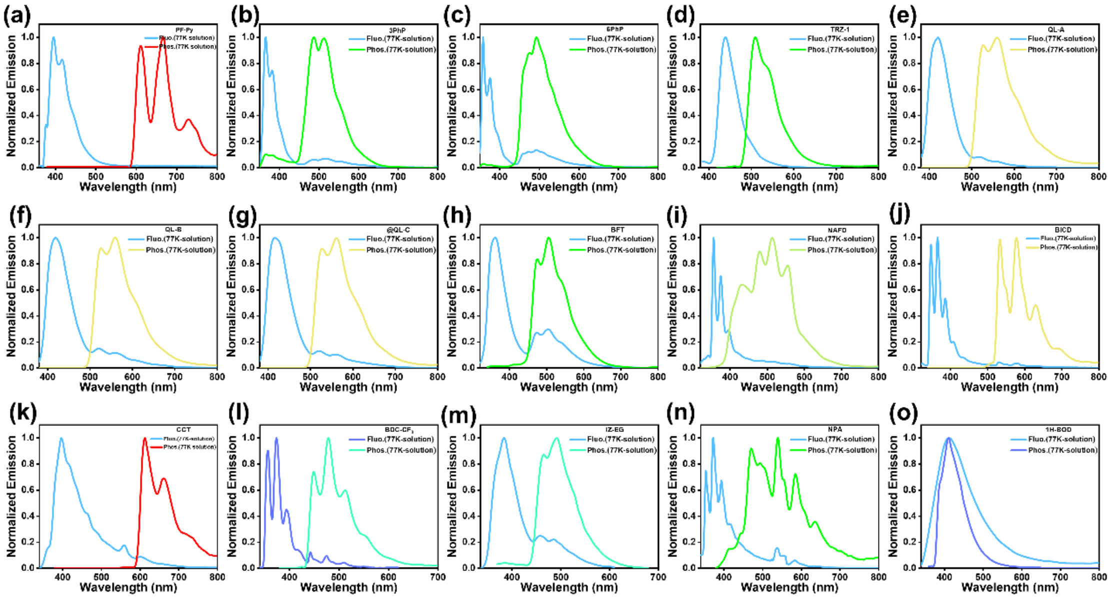

Figure S11. Emission spectra of guests at 77 K (Ex. of fluo.: 340 nm; Ex. of phos.: 360 nm, delayed time: 0.1 ms).

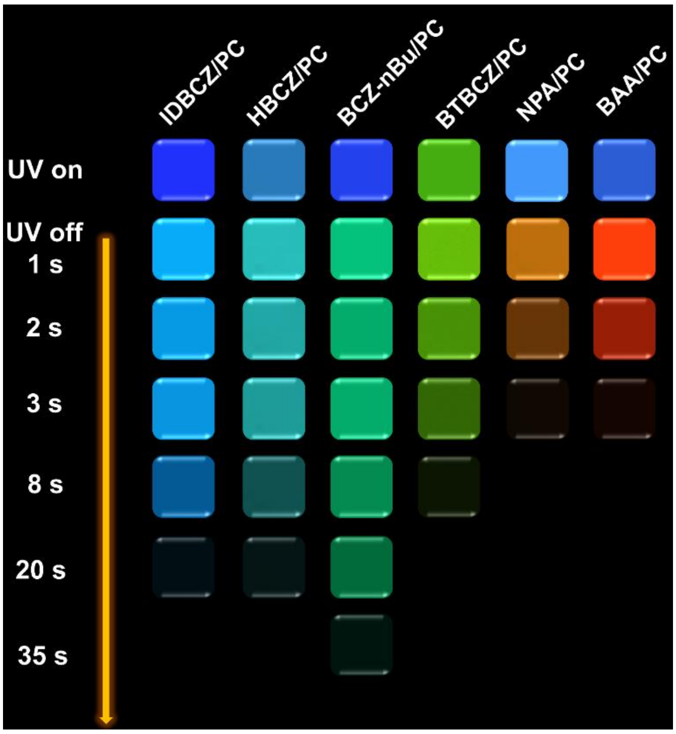

Figure S12. Luminescence photos of the reference doped materials.

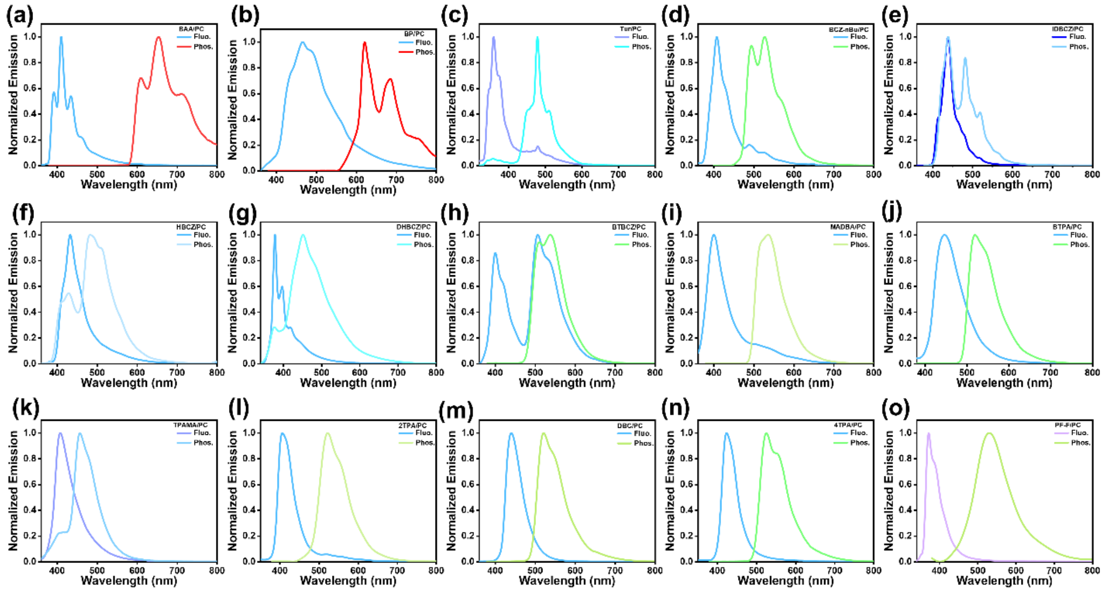

Figure S13. (a) Prompt and delayed emission spectra of doped materials (Ex. of prompt emission: 340 nm; Ex. of delayed emission: 360 nm, delayed time: 0.1 ms).

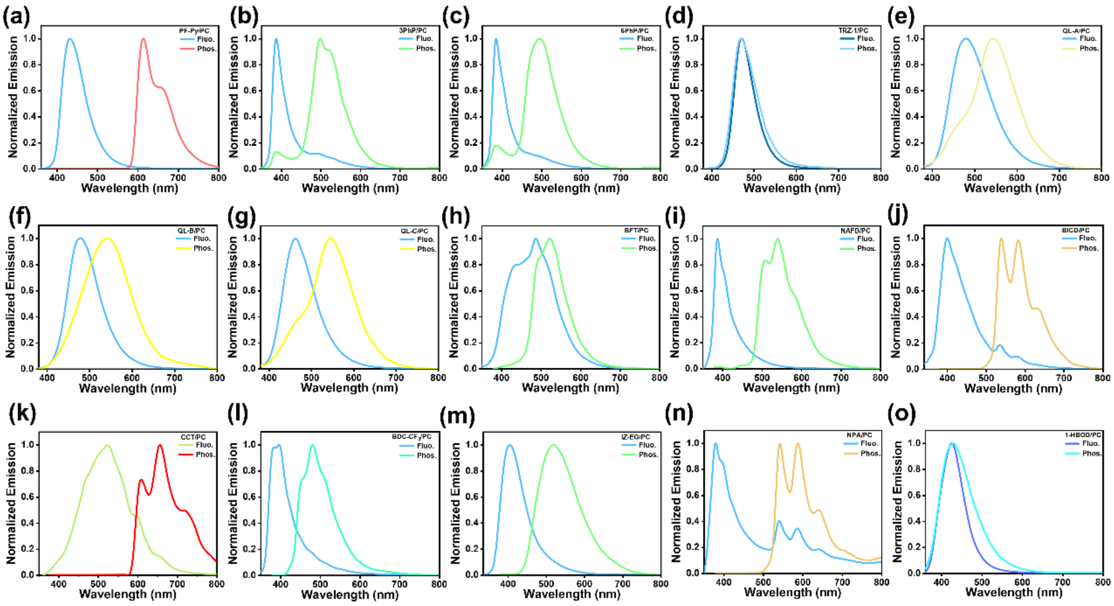

Figure S14 (a) Prompt and delayed emission spectra of doped materials (Ex. of prompt emission: 340 nm; Ex. of delayed emission: 360 nm, delayed time: 0.1 ms).

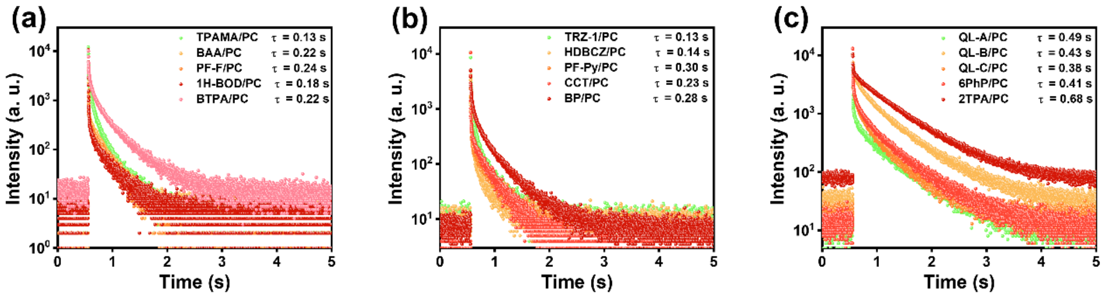

Figure S15. (a-c) Phosphorescence decay curves of doped materials.

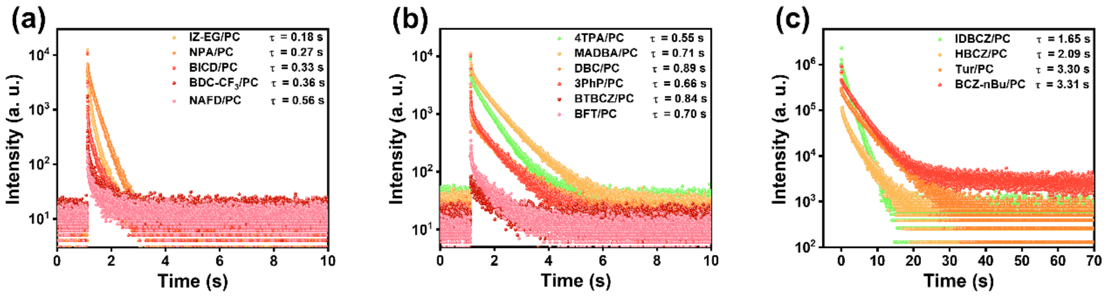

Figure S16. (a-c) Phosphorescence decay curves of doped materials.

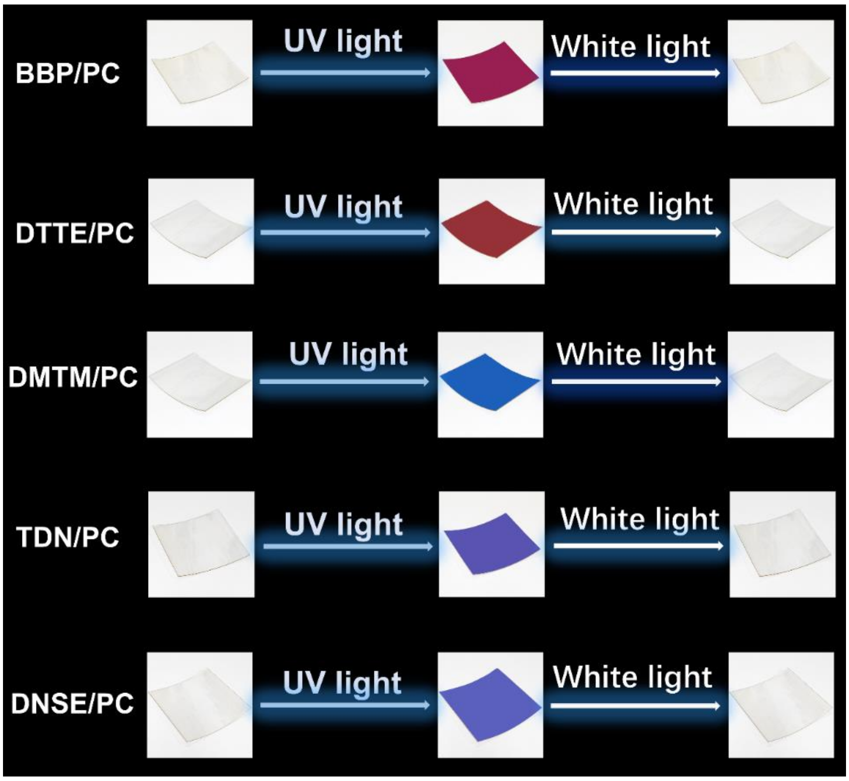

Figure S17. Appearance color change of doped films under different light irradiation.

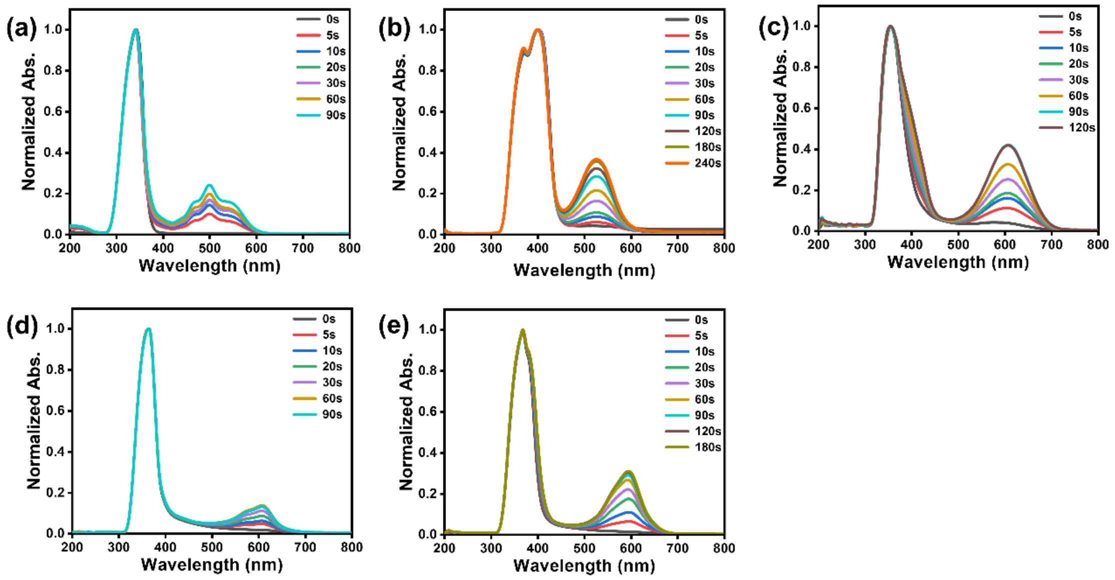

Figure S18. Time-dependent absorption spectra change of original doped film **BBP/PC** (a), **DTTE/PC** (b), **DMTM/PC** (c), **TDN/PC** (d), and **DNSE/PC** (e) upon UV-light irradiation. (UV light wavelength:365nm.)

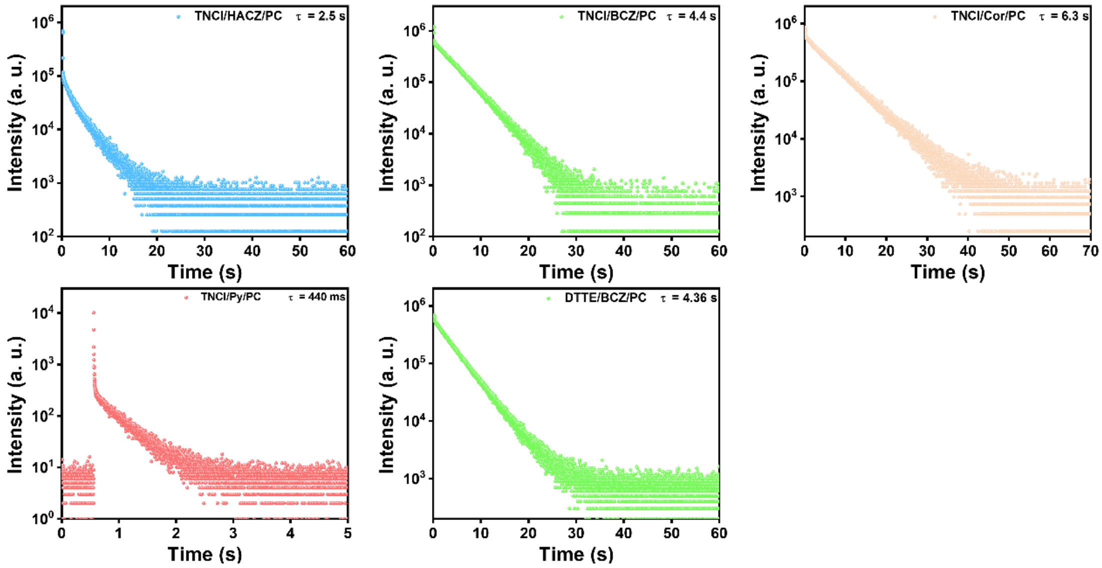

Figure S19. Phosphorescence decay curves of three-component doped materials (**DTTE/BCZ/PC**, **TNCI/HACZ/PC**, **TNCI/BCZ/PC**, **TNCI/Cor/PC**, **Py/Cor/PC**).

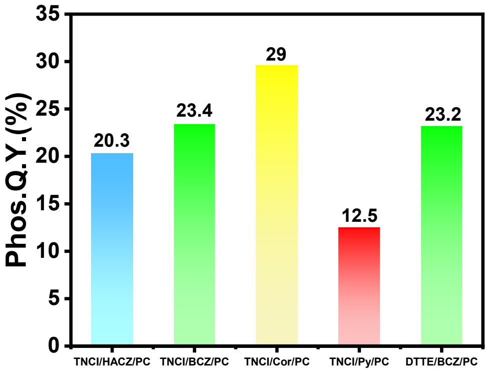

Figure S20. Phosphorescence Q.Y. of three-component doped materials (**DTTE/BCZ/PC**, **TNCI/HACZ/PC**, **TNCI/BCZ/PC**, **TNCI/Cor/PC**, **Py/Cor/PC**).

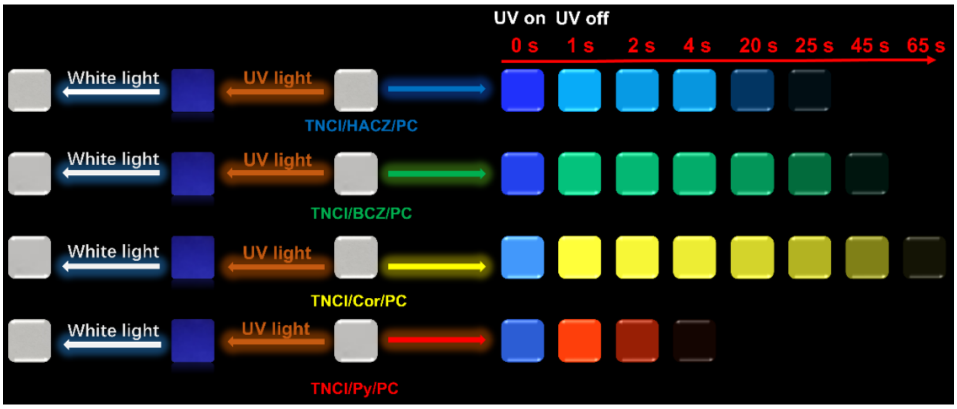

Figure S21. Luminescence photos and appearance color change photos of three-component doped materials.

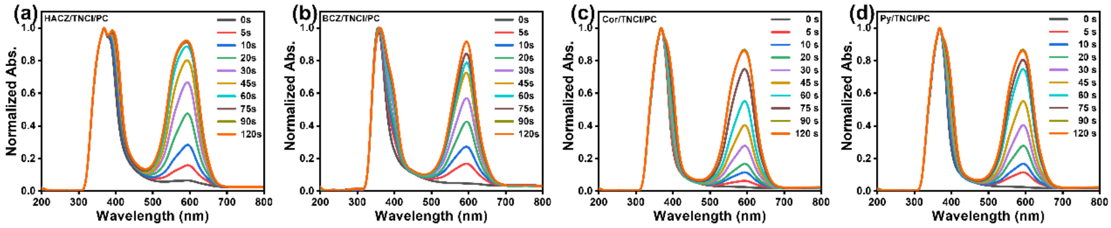

Figure S22. Photochromic property of three-component doped materials **TNCI/HACZ/PC**, **TNCI/BCZ/PC**, **TNCI/Cor/PC**, **Py/Cor/PC**. (UV light wavelength:360 nm.)

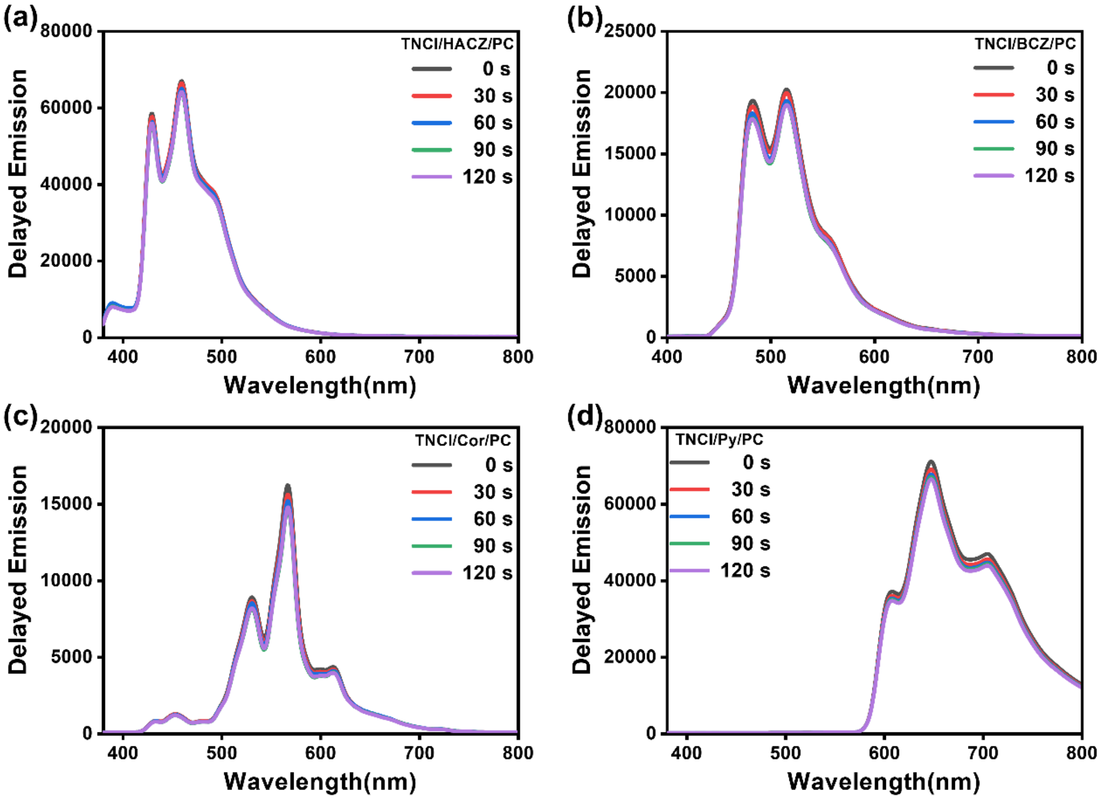

Figure S23. (a) Phosphorescence emission of **TNCI/HACZ/PC** under different UV irradiation durations (Ex. wavelength: 320 nm; delayed time: 0.1 ms). (b)

Phosphorescence emission of **TNCI/BCZ/PC** under different UV irradiation durations (Ex. wavelength: 380 nm; delayed time: 0.1 ms). (c) Phosphorescence emission of **TNCI/Cor/PC** under different UV irradiation durations (Ex. wavelength: 360 nm; delayed time: 0.1 ms). (d) Phosphorescence emission of **TNCI/Py/PC** under different UV irradiation durations (Ex. wavelength: 360 nm; delayed time: 0.1 ms).

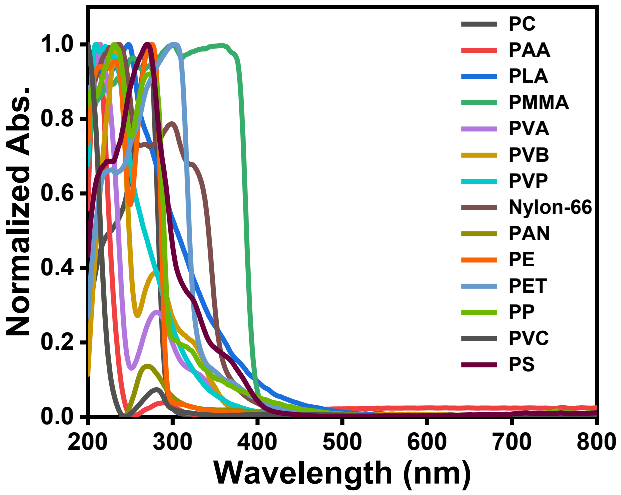

Figure S24. Absorption spectra of all polymers.

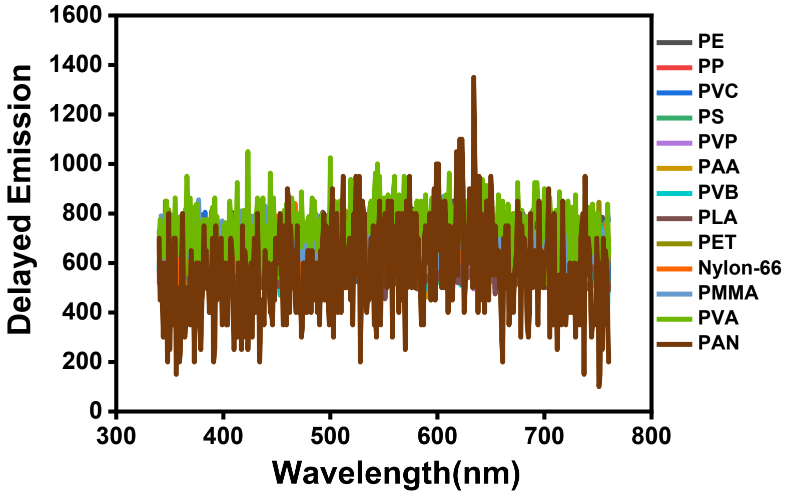

Figure S25. Phosphorescence spectra of all reference polymer hosts (Ex. wavelength: 320 nm; Delayed time: 0.1 ms).

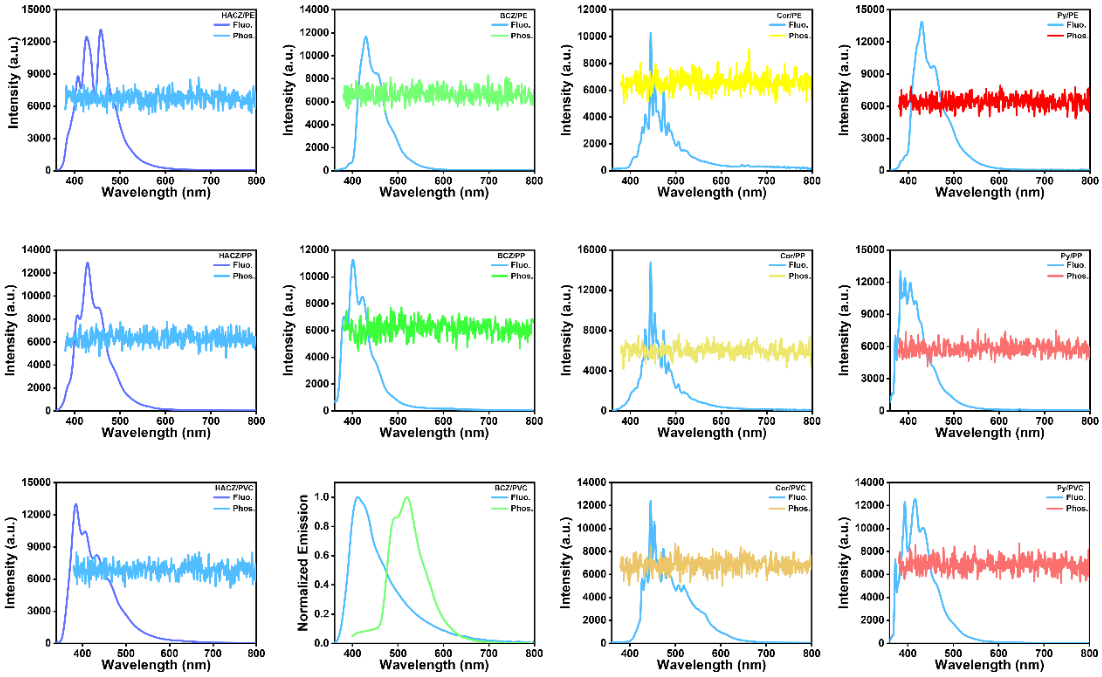

Figure S26. Emission spectra of the doped materials (Ex. of fluo.: 340 nm; Ex. of phos.: 360 nm, delayed time: 0.1 ms).

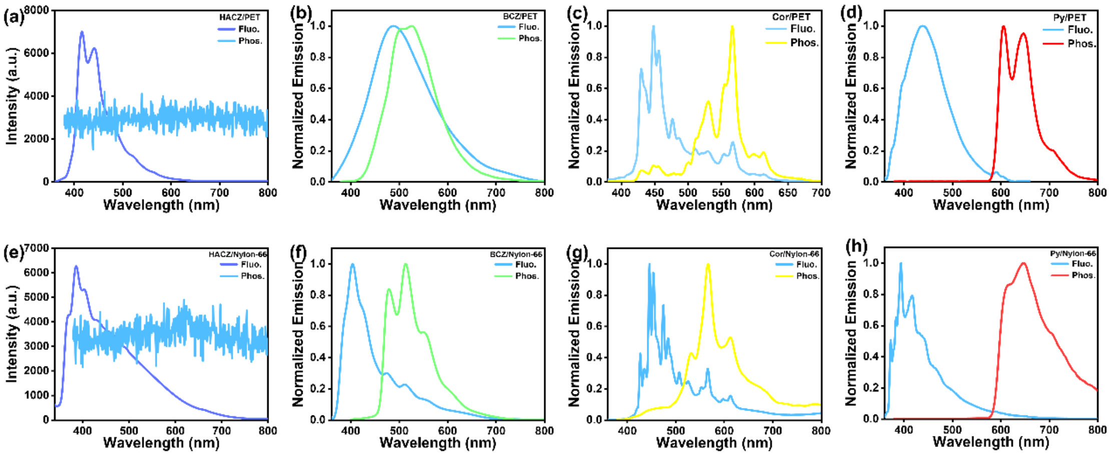

Figure S27. Emission spectra of the doped materials (Ex. of fluo.: 340 nm; Ex. of phos.: 360 nm, delayed time: 0.1 ms).

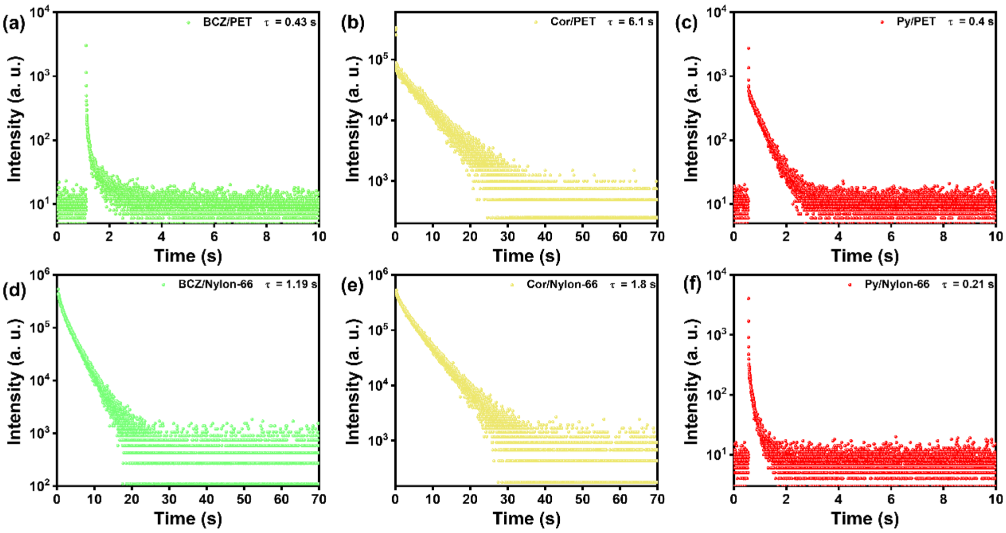

Figure S28. Phosphorescence intensity curves of the doped materials.

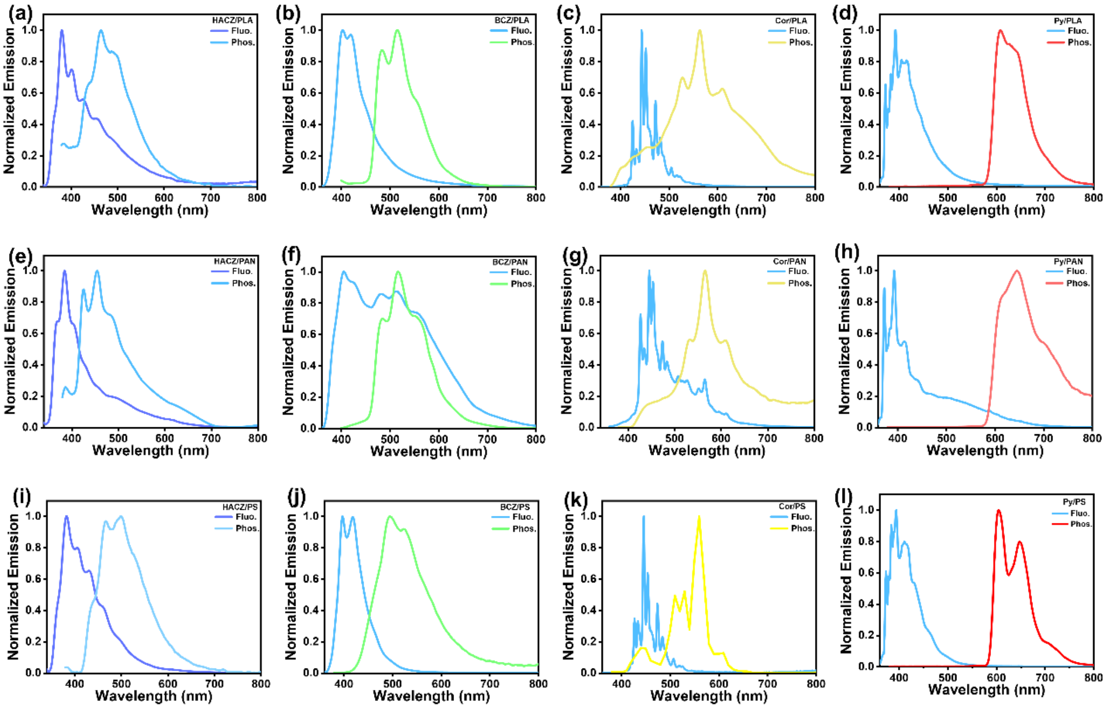

Figure S29. Emission spectra of the doped materials (Ex. of fluo.: 340 nm; Ex. of phos.: 360 nm, delayed time: 0.1 ms).

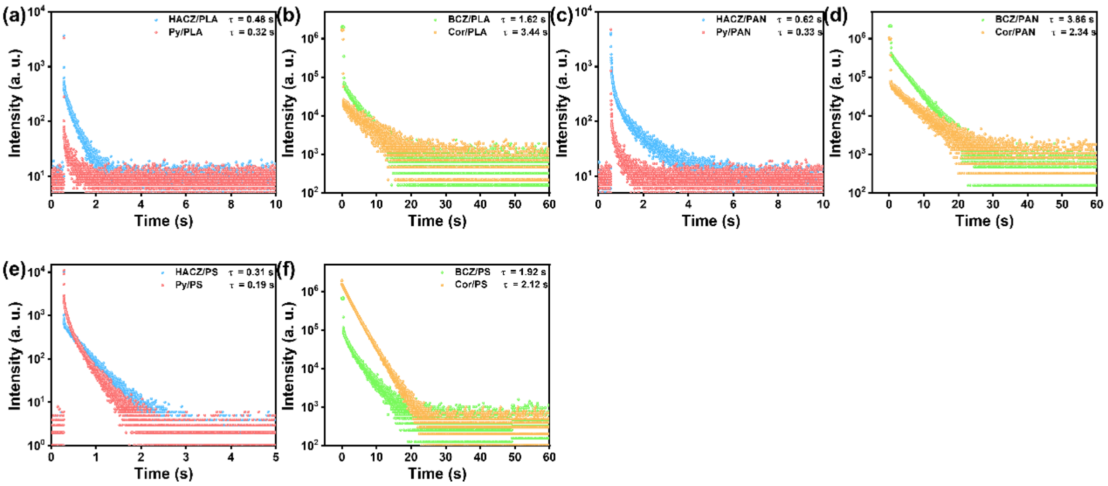

Figure S30. Phosphorescence intensity curves of doped materials.

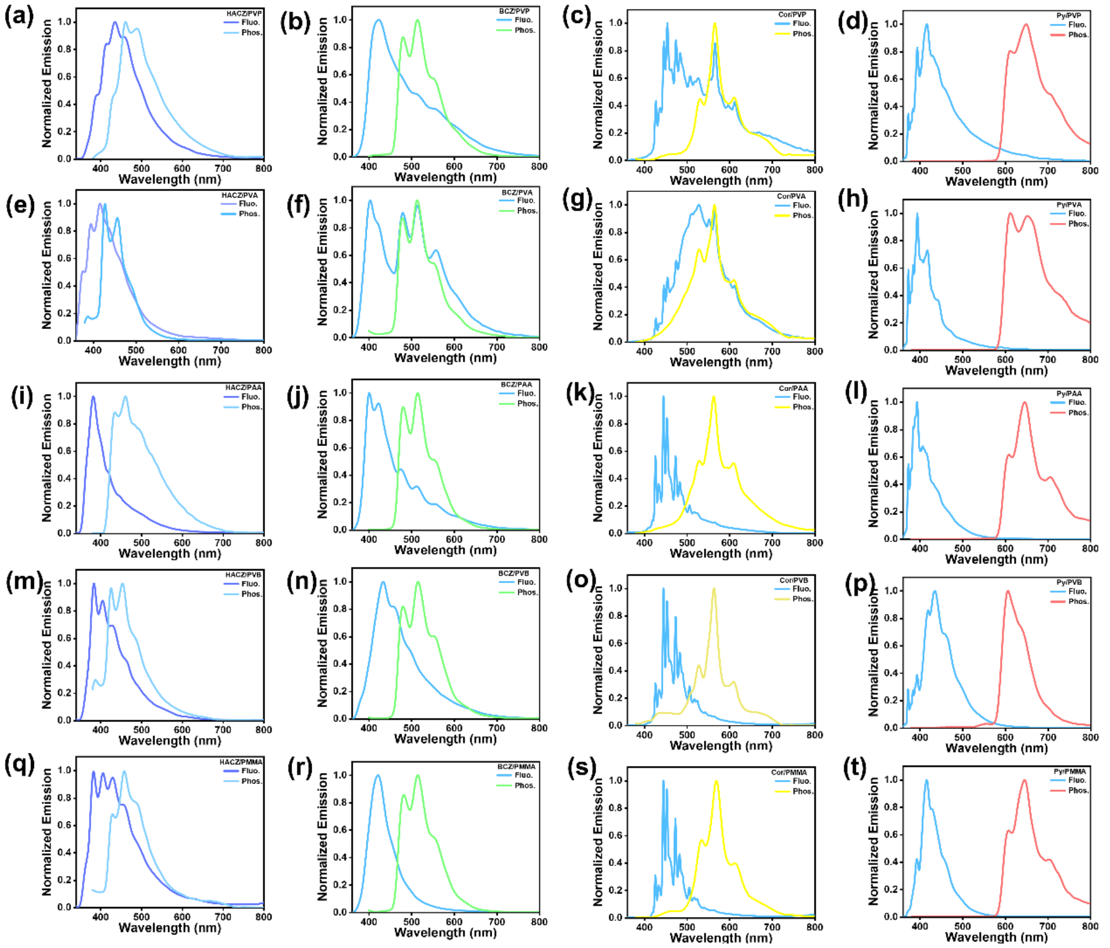

Figure S31. Emission spectra of the doped materials (Ex. of fluo.: 340 nm; Ex. of phos.: 360 nm, delayed time: 0.1 ms).

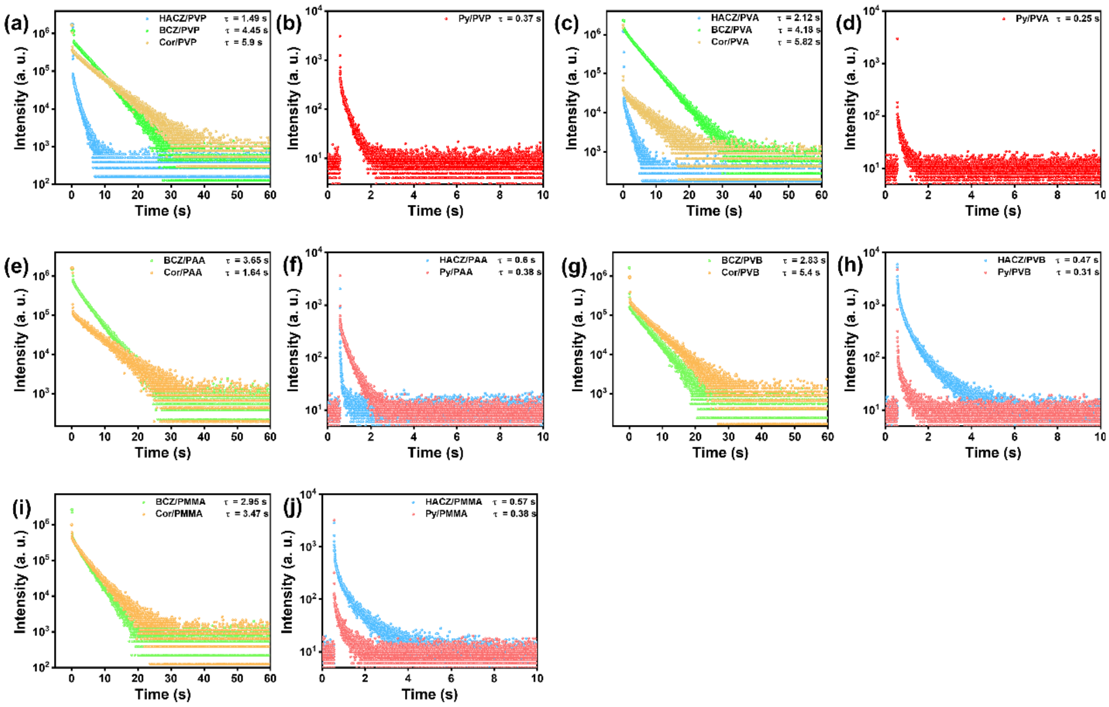

Figure S32. Phosphorescence intensity curves of doped materials.

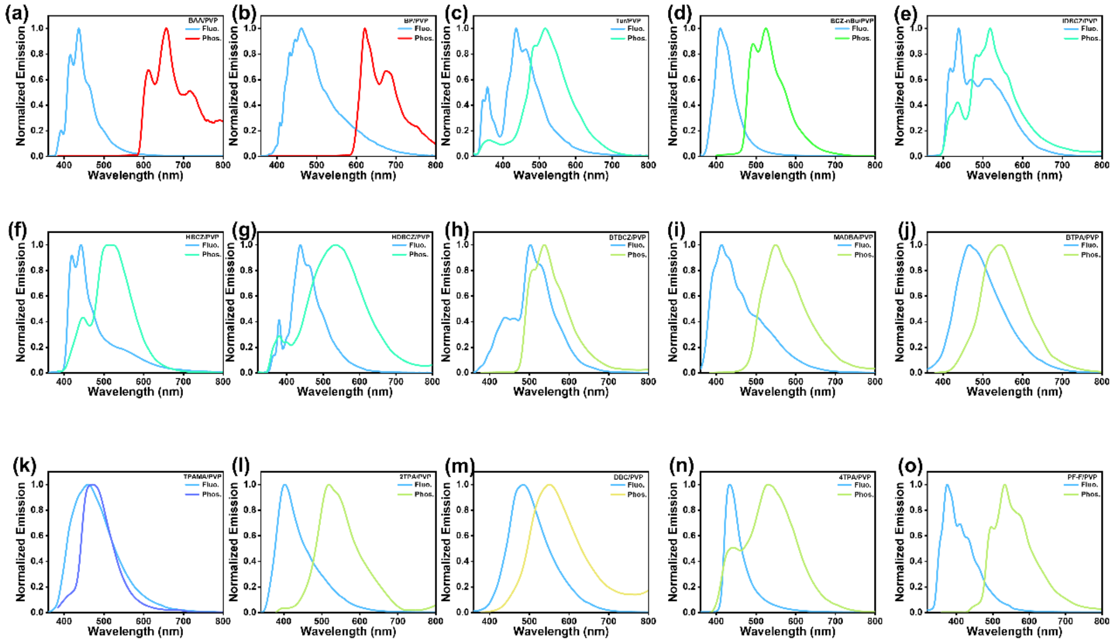

Figure S33. Emission spectra of **PVP**-based doped materials (Ex. of fluo.: 340 nm; Ex. of phos.: 360 nm, delayed time: 0.1 ms).

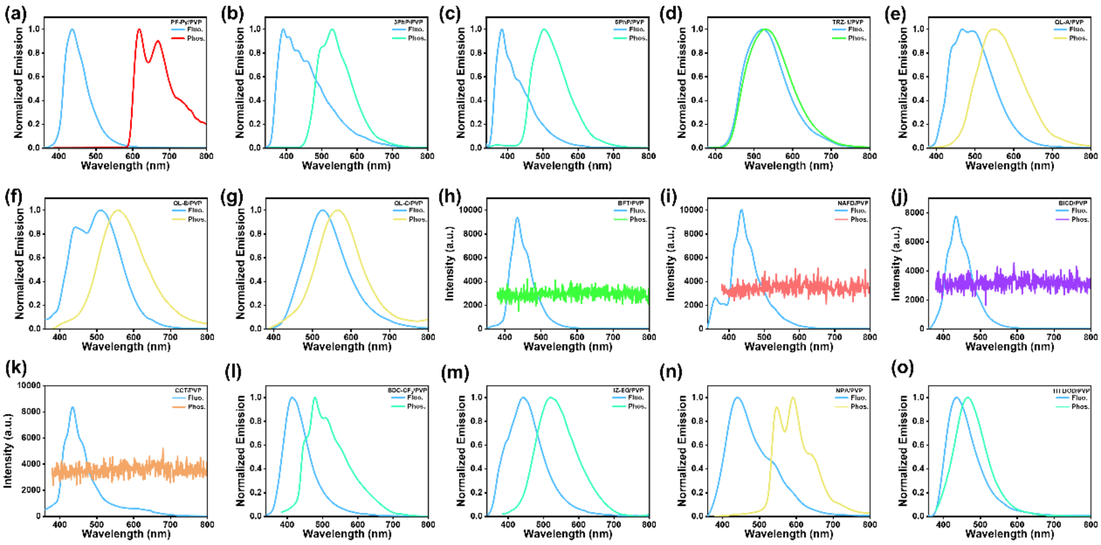

Figure S34. Emission spectra of the **PVP**-based doped materials (Ex. of fluo.: 340 nm; Ex. of phos.: 360 nm, delayed time: 0.1 ms).

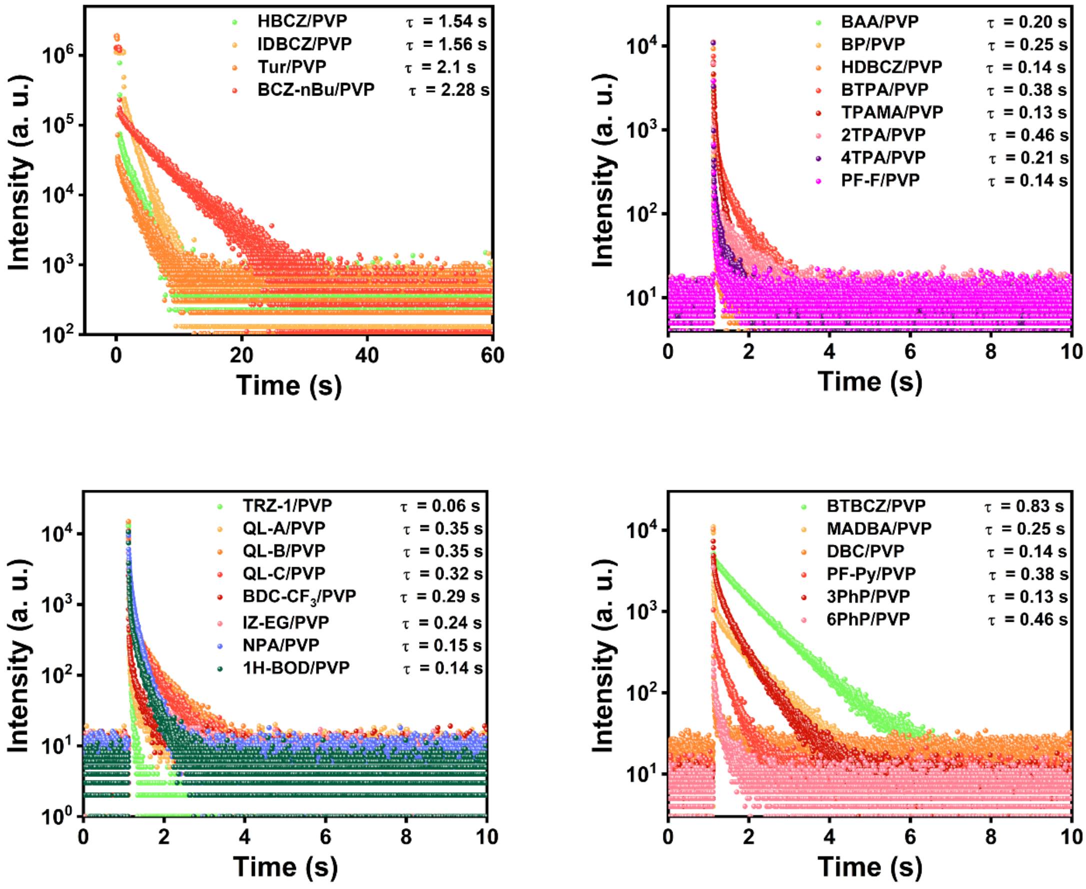

Figure S35. Phosphorescence intensity curves of **PVP**-based doped materials.

Table S1. Phosphorescence lifetime and phosphorescence Q.Y. of **PVP**-based doped materials.

Guest/PVP	**1**	**2**	**3**	**4**	**5**	**6**	**7**	**8**	**9**	**10**
τ_p(s)	0.20	0.25	2.1	2.28	1.56	1.54	0.14	0.83	0.66	0.38
Q.Y.(%)	7.7	7.3	13.0	18.4	14.7	6.9	18.0	9.4	13.0	10.1
Guest/PVP	**11**	**12**	**13**	**14**	**15**	**16**	**17**	**18**	**19**	**20**
τ_p(s)	0.13	0.46	0.56	0.21	0.14	0.32	0.47	0.35	0.06	0.35
Q.Y.(%)	12.2	8.2	16.0	10.7	8.0	9.9	13.8	12.6	12.2	10.8
Guest/PVP	**21**	**22**	**23**	**24**	**25**	**26**	**27**	**28**	**29**	**30**
τ_p (s)	0.35	0.32	0	0	0	0	0.29	0.24	0.15	0.14
Q.Y.(%)	12.1	11.4	--	--	--	--	10.4	9.6	8.7	7.6

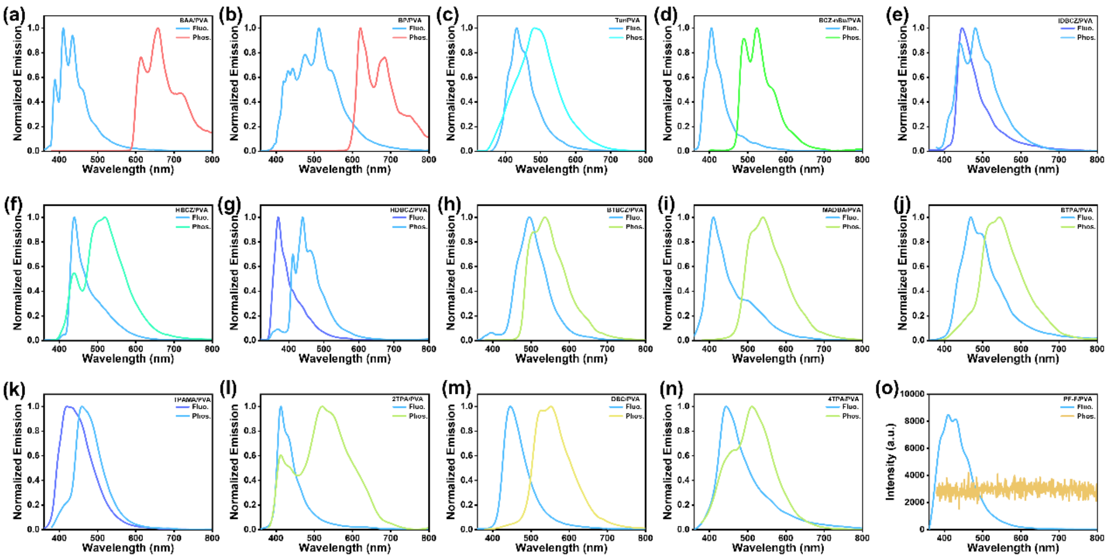

Figure S36. Emission spectra of the **PVA**-based doped materials (Ex. of fluo.: 340 nm; Ex. of phos.: 360 nm, delayed time: 0.1 ms).

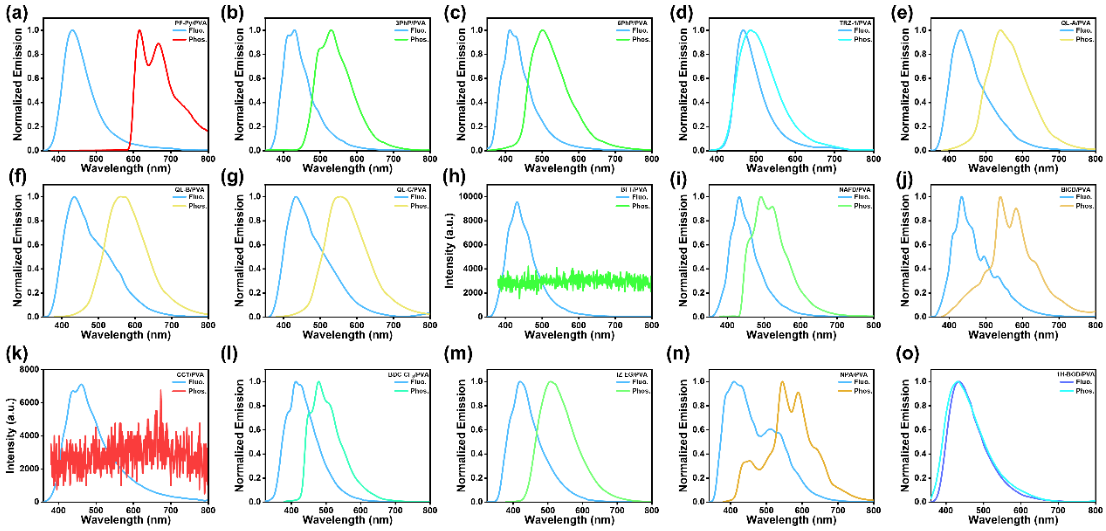

Figure S37. Emission spectra of the **PVA**-based doped materials (Ex. of fluo.: 340 nm; Ex. of phos.: 360 nm, delayed time: 0.1 ms).

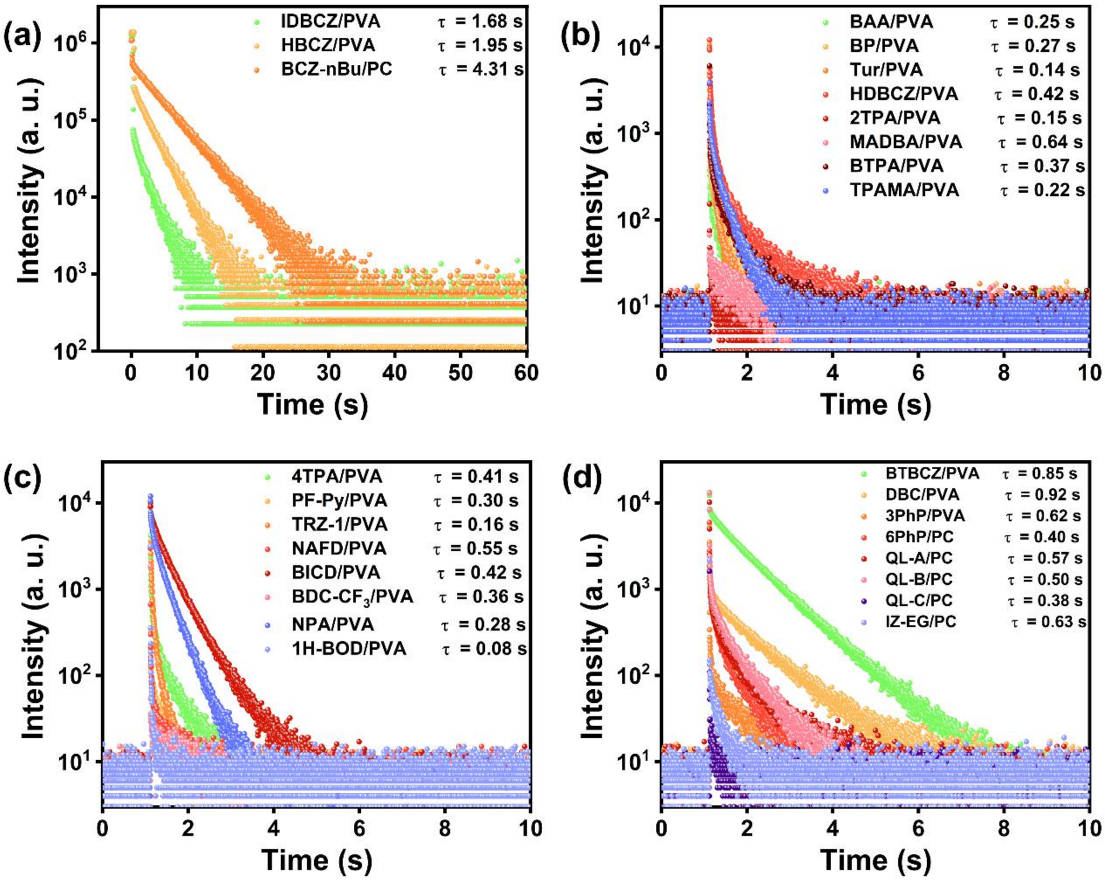

Figure S38. Phosphorescence intensity curves of **PVA**-based doped materials.

Table S2 Phosphorescence lifetime and phosphorescence Q.Y. of **PVA**-based doped materials.

Guest/PVA	**1**	**2**	**3**	**4**	**5**	**6**	**7**	**8**	**9**	**10**
τ_p(s)	0.25	0.27	0.14	4.31	1.68	1.95	0.42	0.85	0.64	0.37
Q.Y.(%)	8.6	9.1	2.5	16.0	10.9	14.1	19.1	15.1	7.8	9.5
Guest/PVA	**11**	**12**	**13**	**14**	**15**	**16**	**17**	**18**	**19**	**20**
τ_p(s)	0.22	0.15	0.92	0.41	0	0.30	0.62	0.40	0.16	0.57
Q.Y.(%)	11.4	9.3	7.4	8.4	--	6.8	15.7	8.5	3.6	11.6
Guest/PVA	**21**	**22**	**23**	**24**	**25**	**26**	**27**	**28**	**29**	**30**
τ_p (s)	0.50	0.38	0	0.55	0.42	0	0.36	0.63	0.28	0.08
Q.Y.(%)	10.1	12.1	--	6.6	5.4	--	8.9	14.7	7.2	16.0

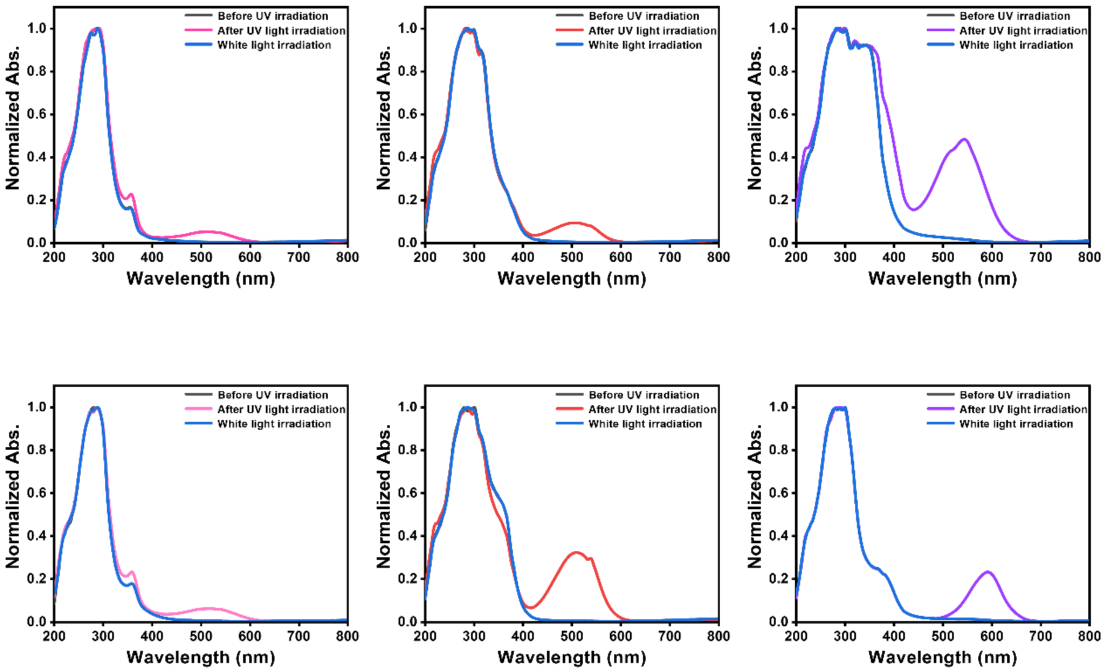

Figure S39. Photochromic property of the doped materials.

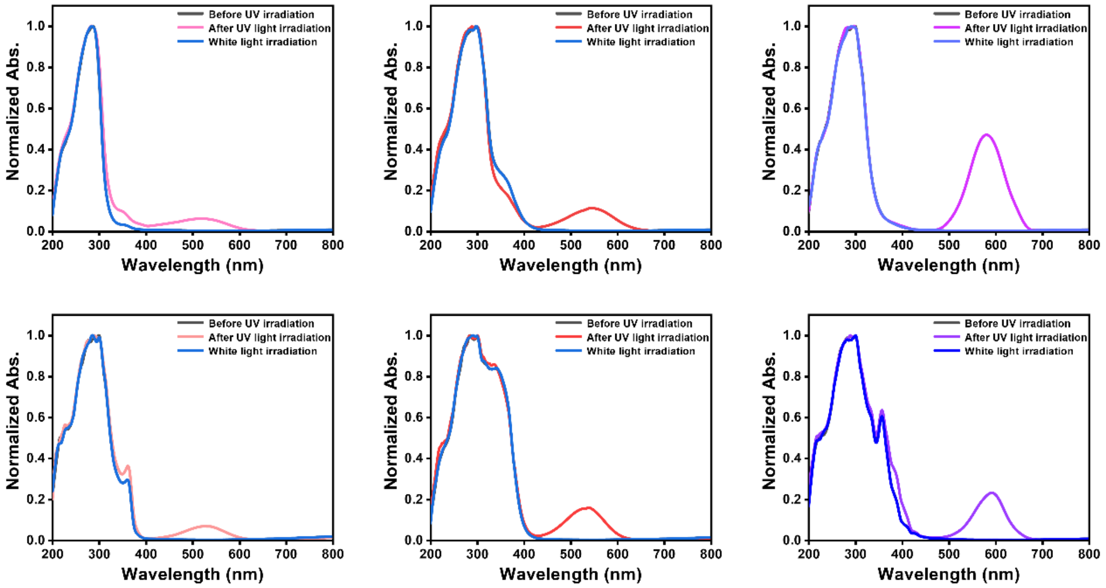

Figure S40. Photochromic property of the doped materials.

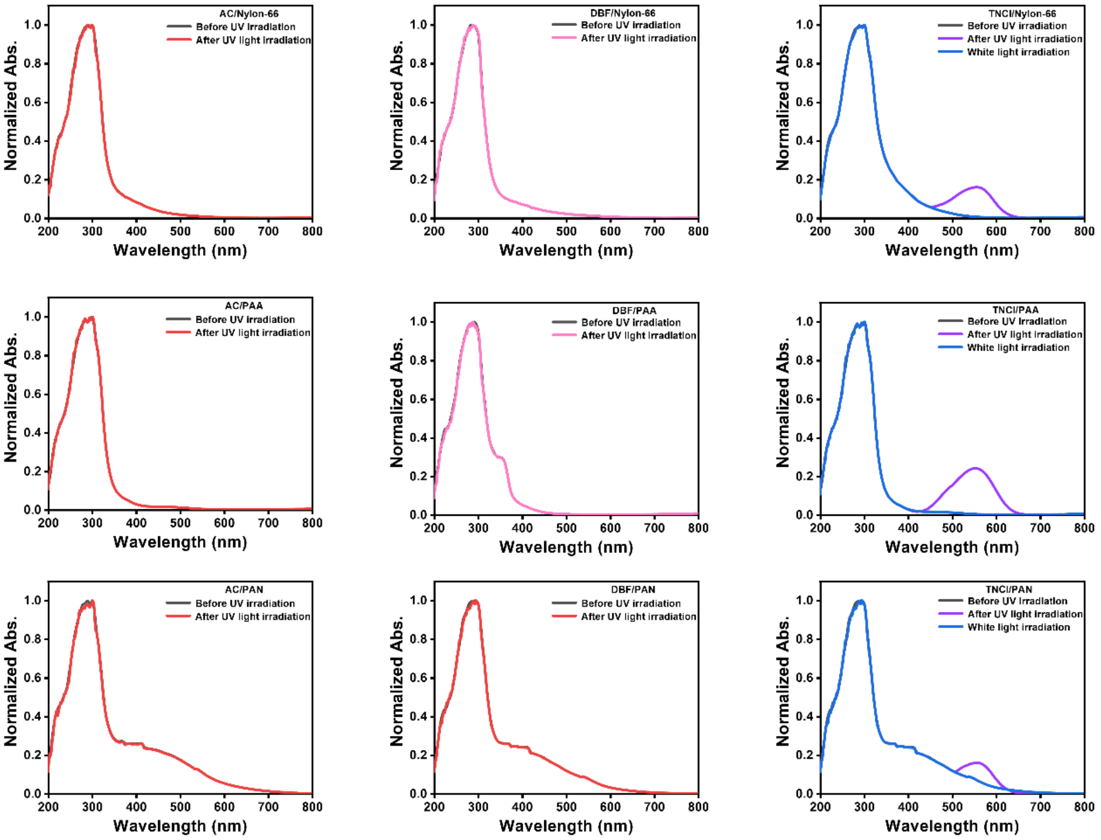

Figure S41. Photochromic property of the doped materials.

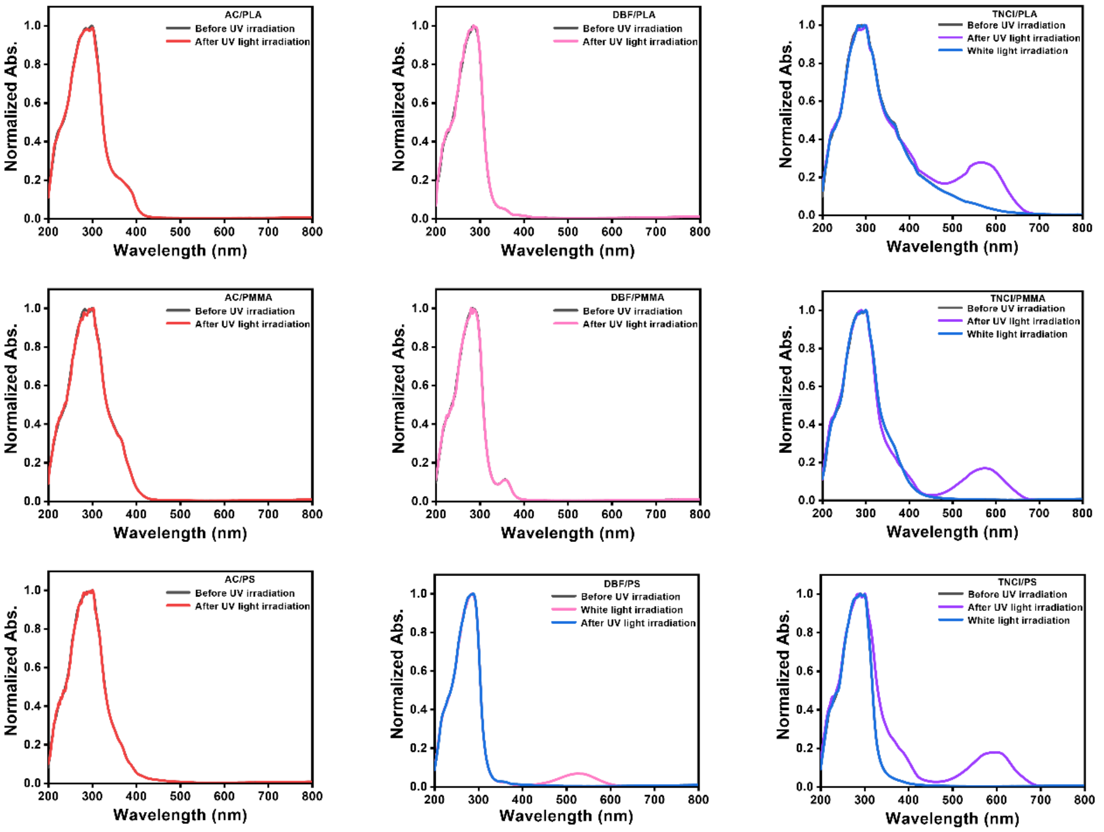

Figure S42. Photochromic property of the doped materials.

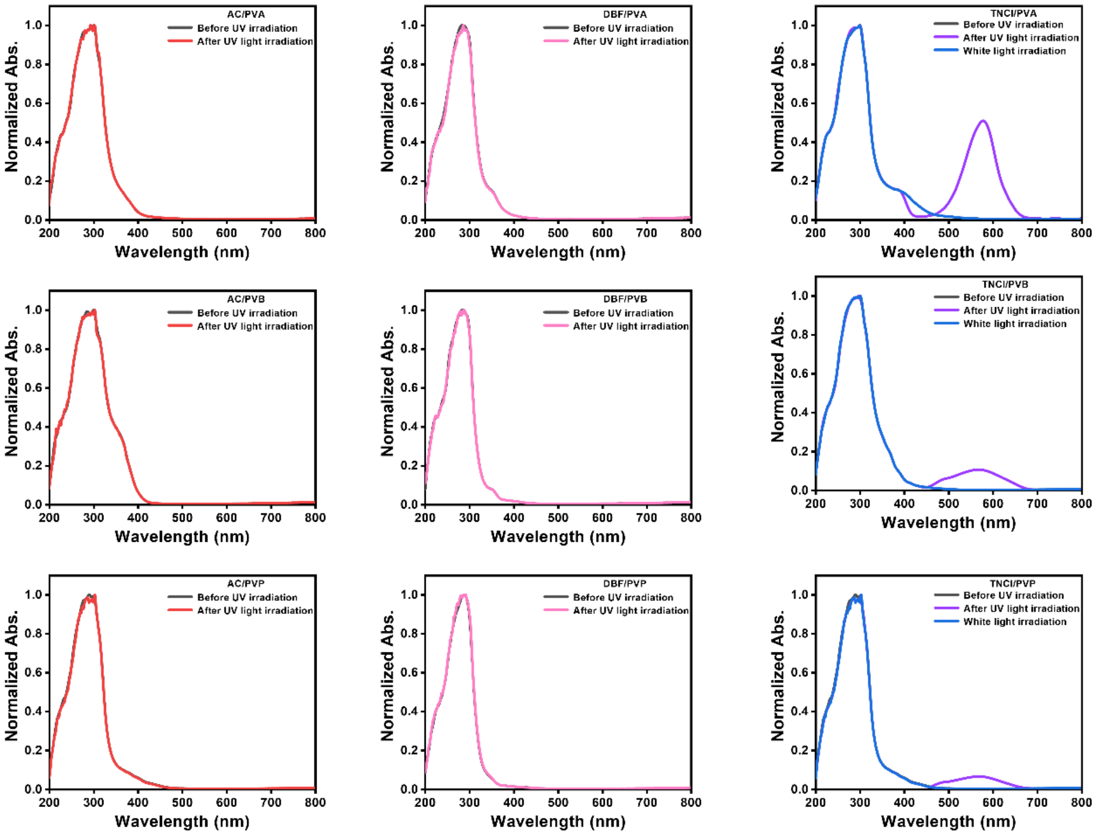

Figure S43. Photochromic property of the doped materials.

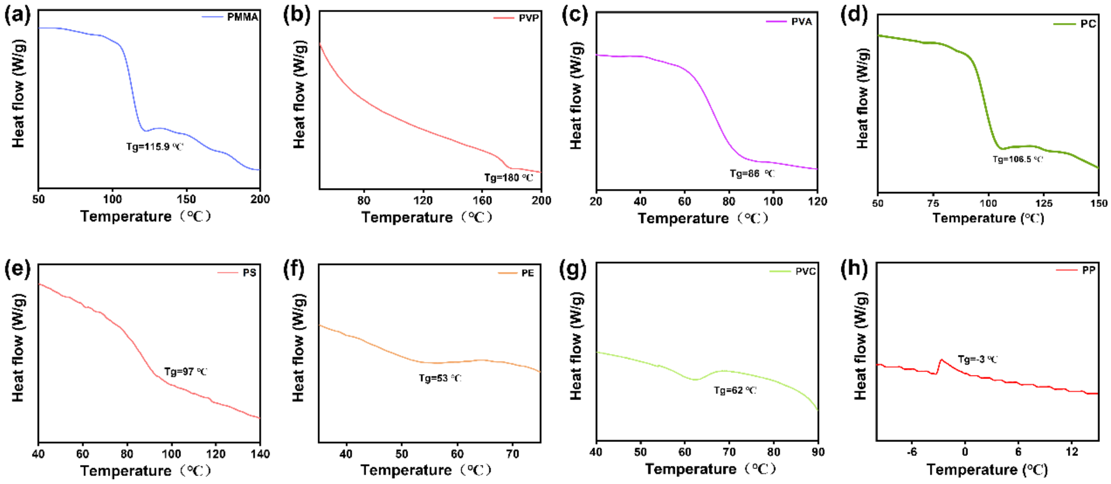

Figure S44. DSC spectra of host **PMMA** (a), of host **PVP** (b), of host **PVA** (c), of host **PC** (d), of host **PS** (e), of host **PE** (f), of host **PVC** (g),of host **PP** (h).

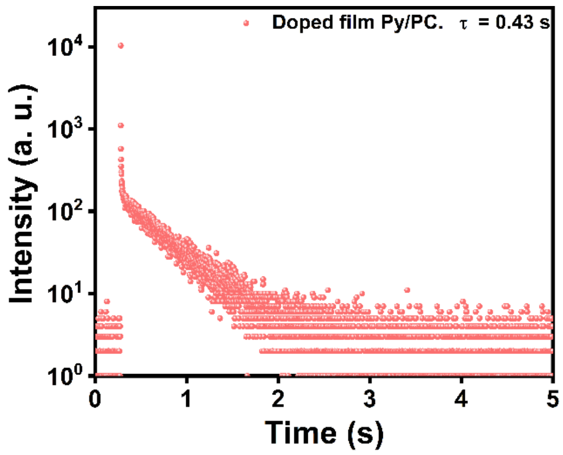

Figure S45. Phosphorescence intensity curve of doped film **Py/PC**.

3. Spectra of NMR

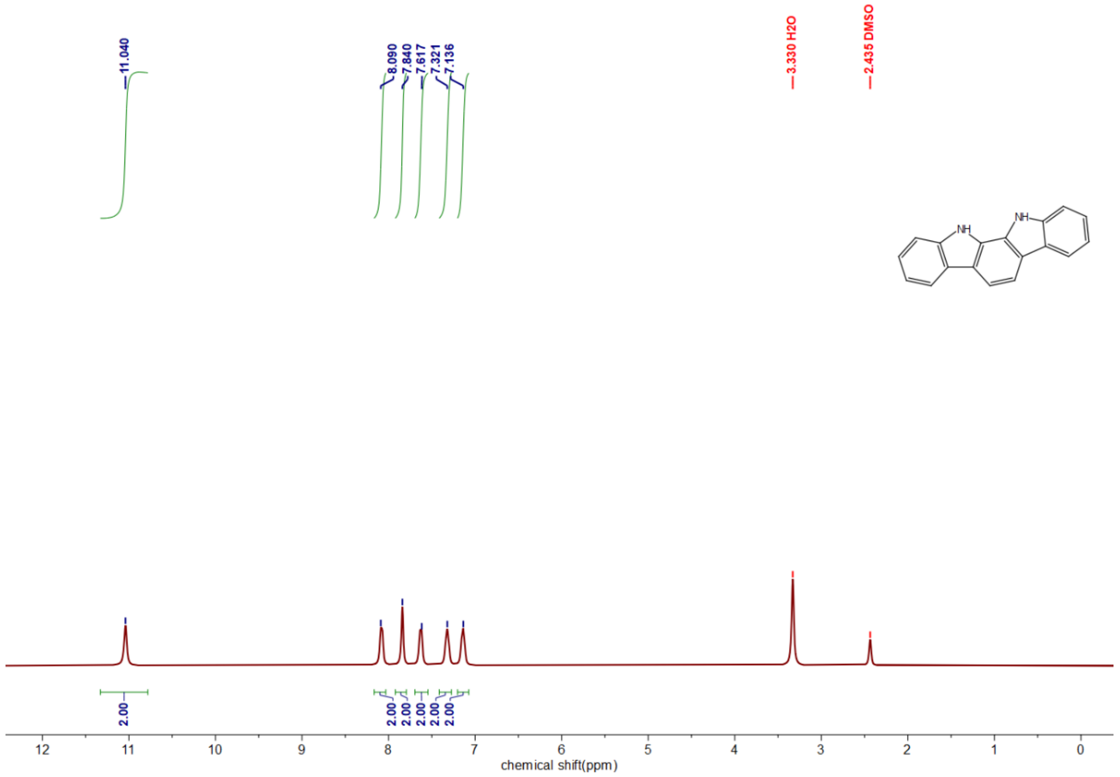

Figure S46. ^{1}H NMR of **HACZ** (DMSO-*d6*, 400 MHz).

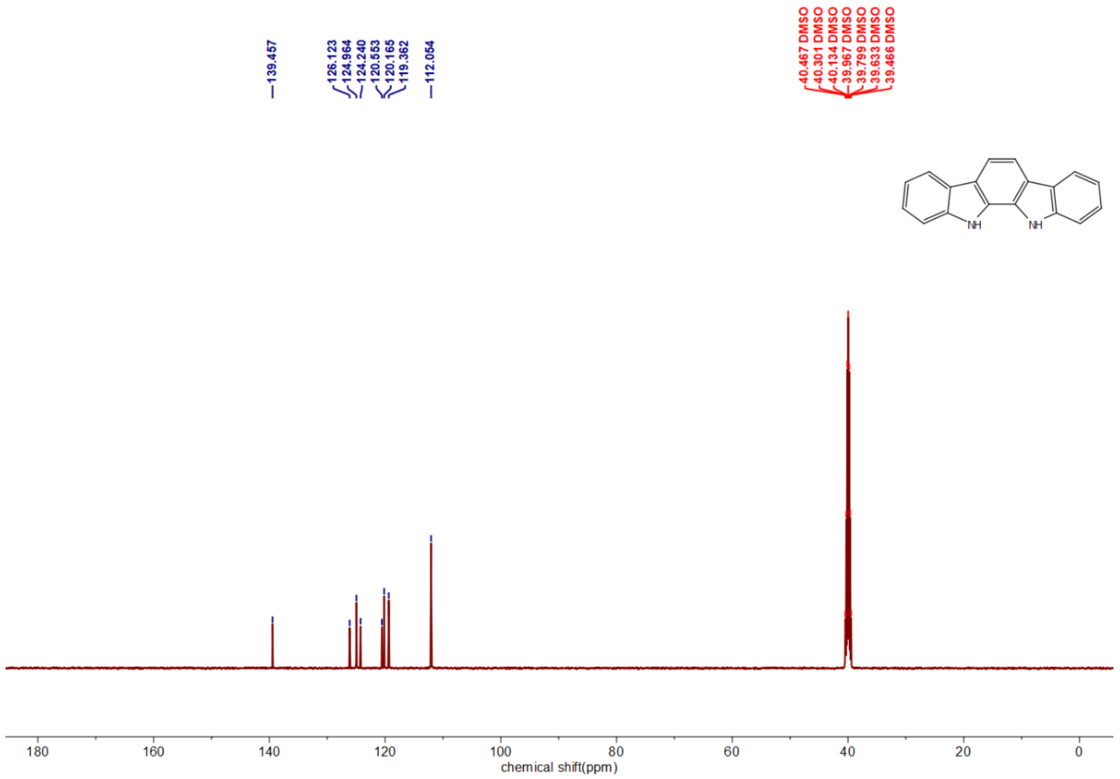

Figure S47. ^{13}C NMR of **HACZ** (DMSO-*d6*, 126 MHz).

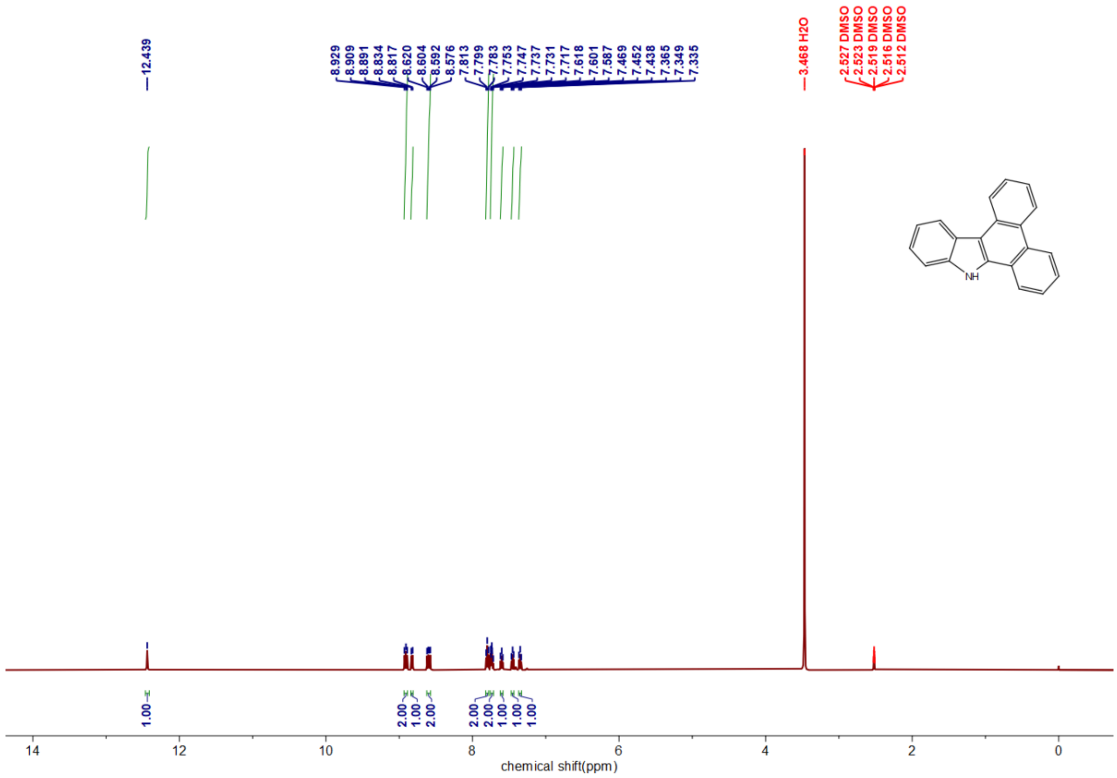

Figure S48. ^{1}H NMR of **BCZ** (DMSO-*d6*, 500 MHz).

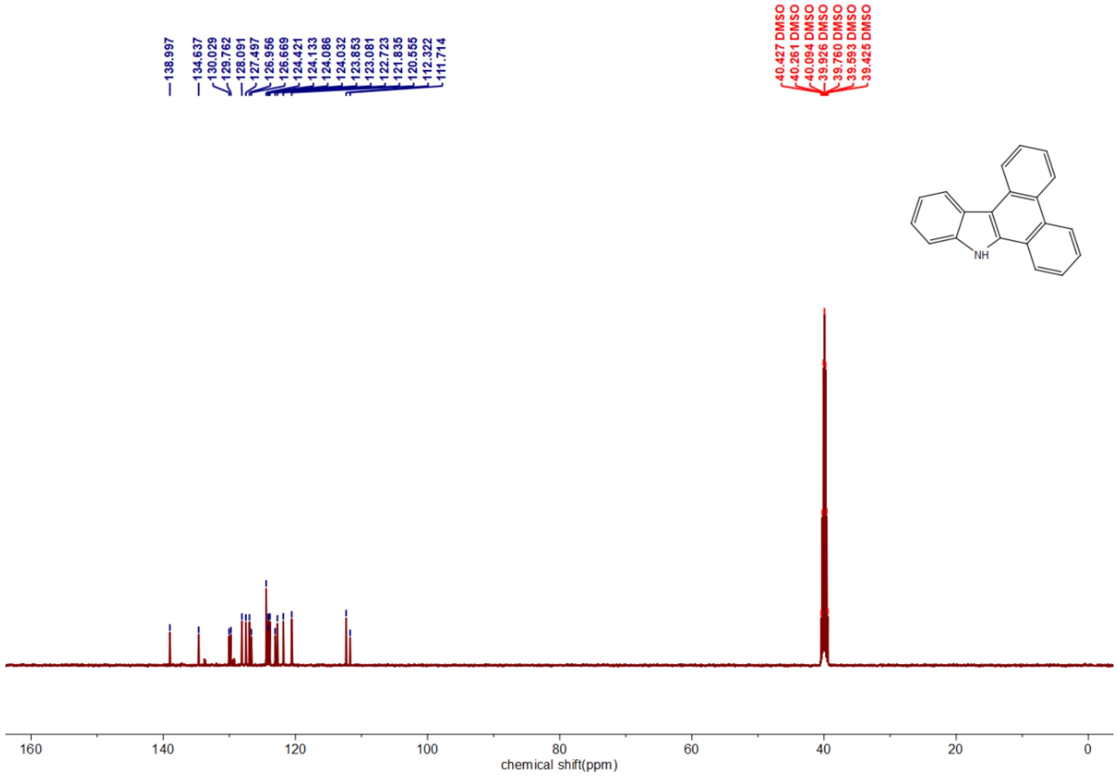

Figure S49. ^{13}C NMR of **BCZ** (DMSO-*d6*, 126 MHz).

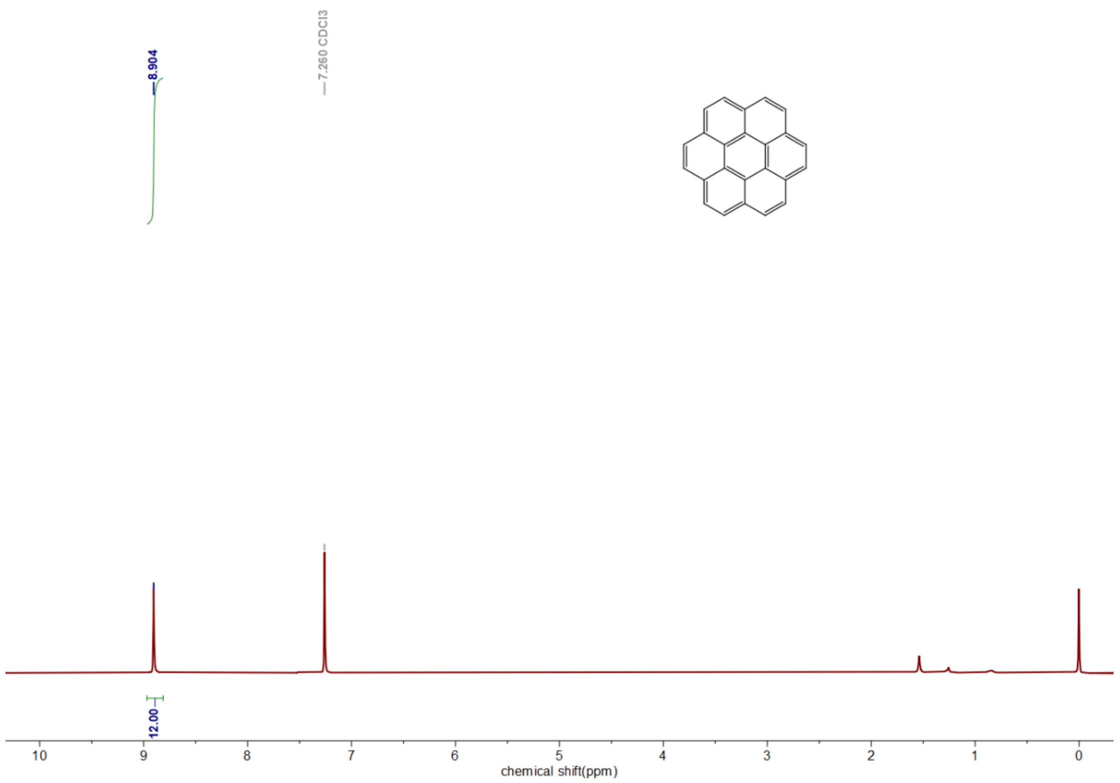

Figure S50. ^{1}H NMR of **Cor** ($CDCl_3$, 400 MHz).

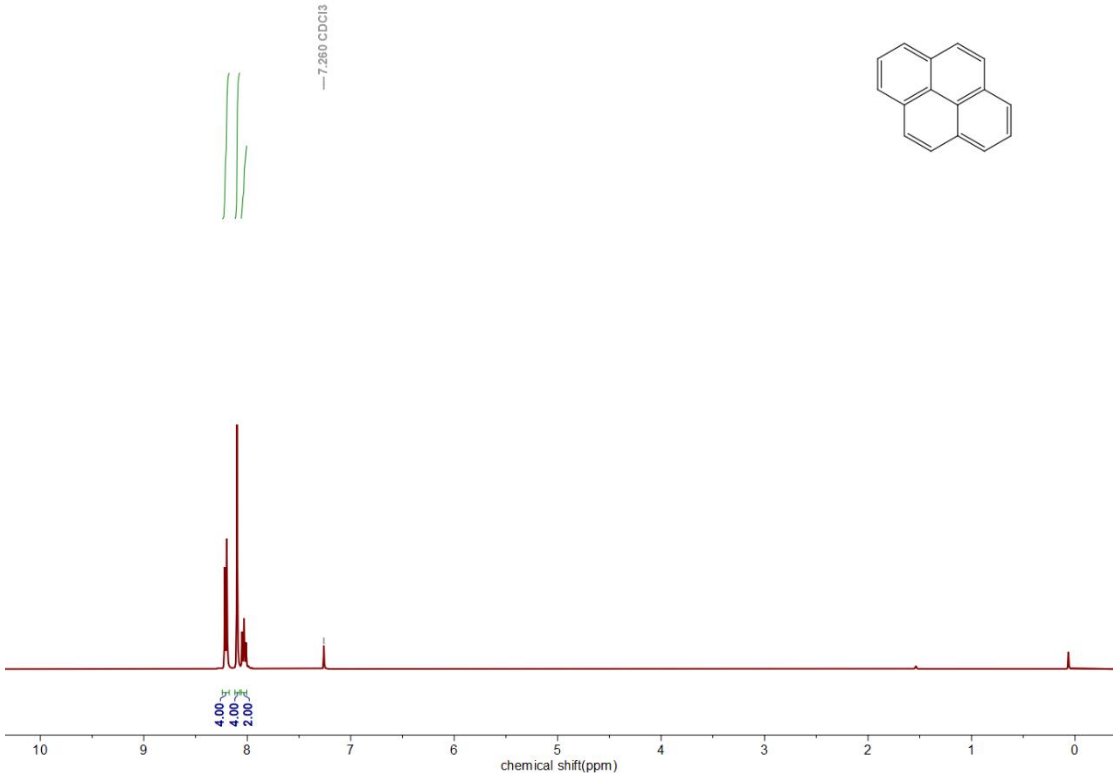

Figure S51. ^{1}H NMR of **Py** ($CDCl_3$, 400 MHz).

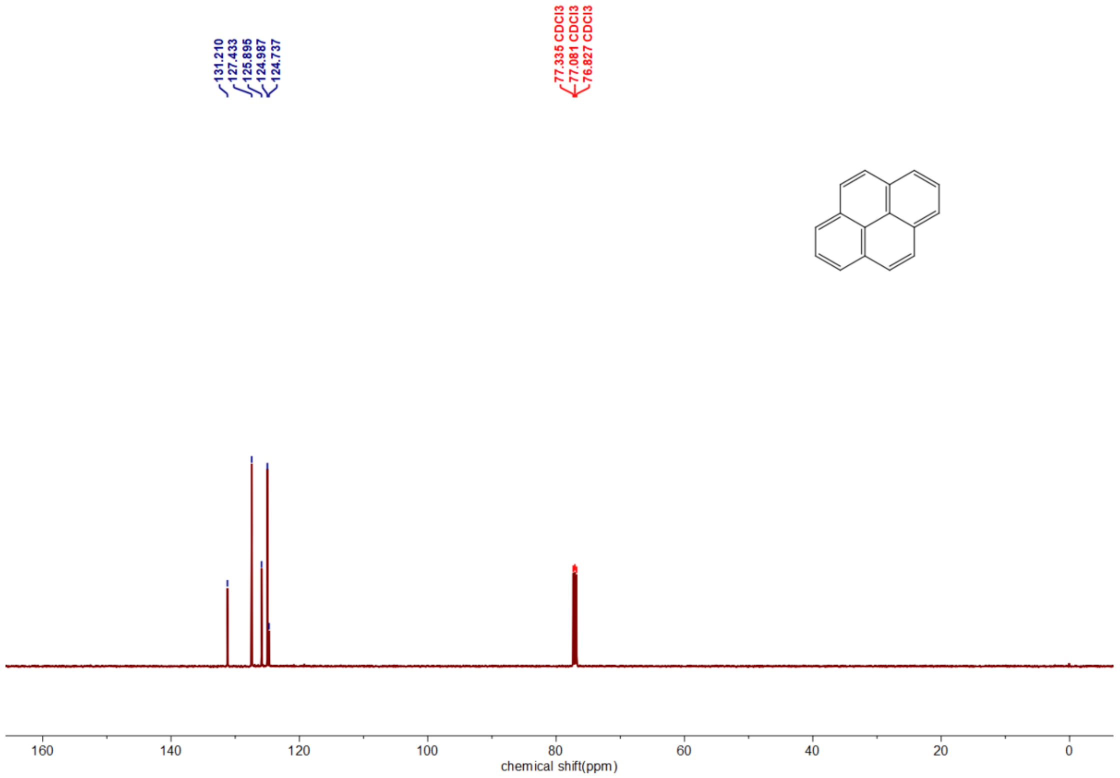

Figure S52. ^{13}C NMR of **Py** ($CDCl_3$, 126 MHz).

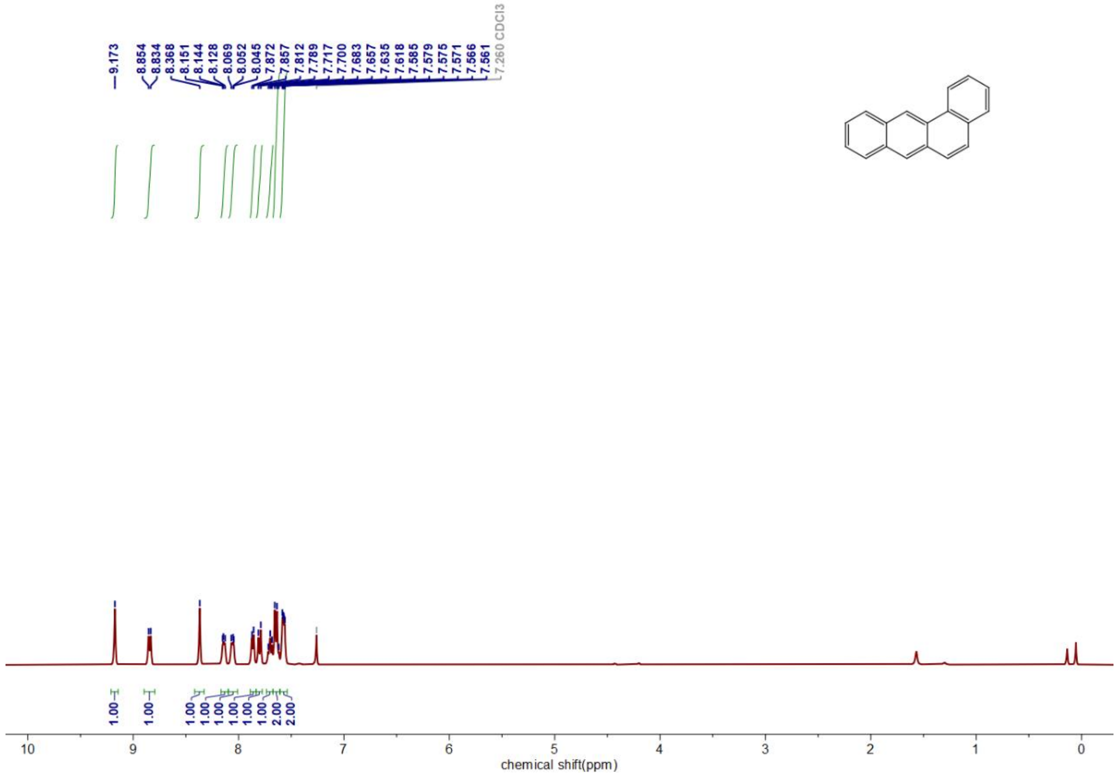

Figure S53. ^{1}H NMR of **BAA** ($CDCl_3$, 400 MHz).

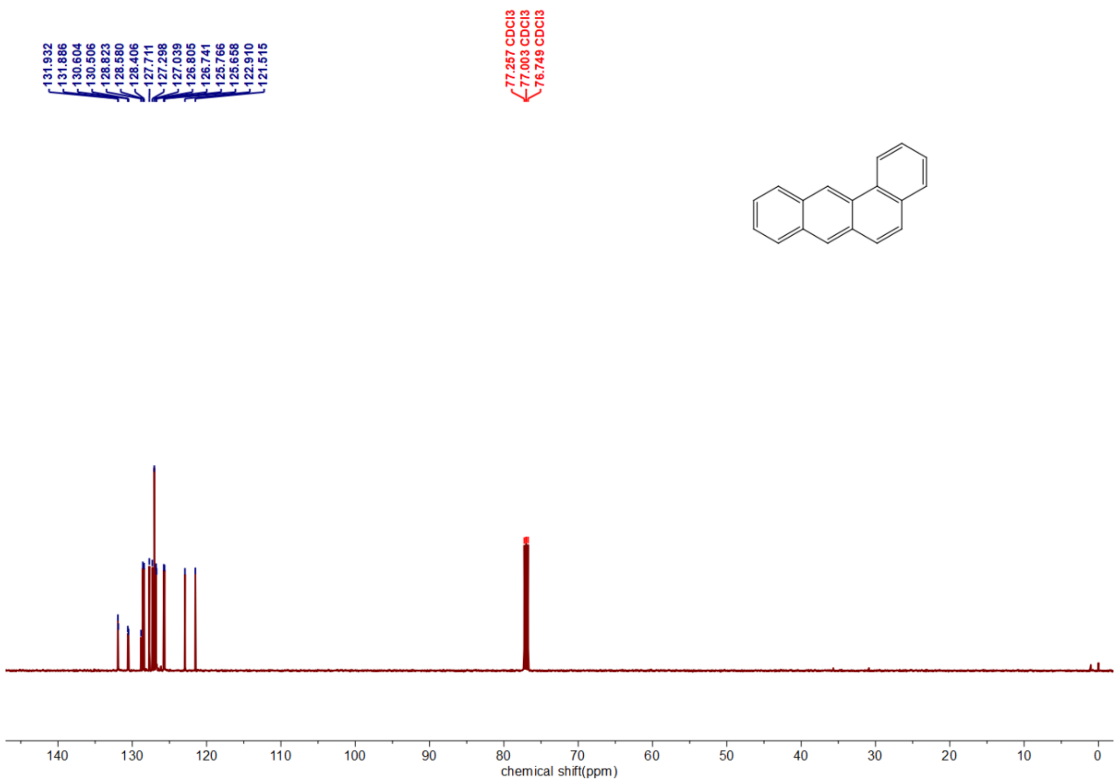

Figure S54. ^{13}C NMR of **BAA** ($CDCl_3$, 126 MHz).

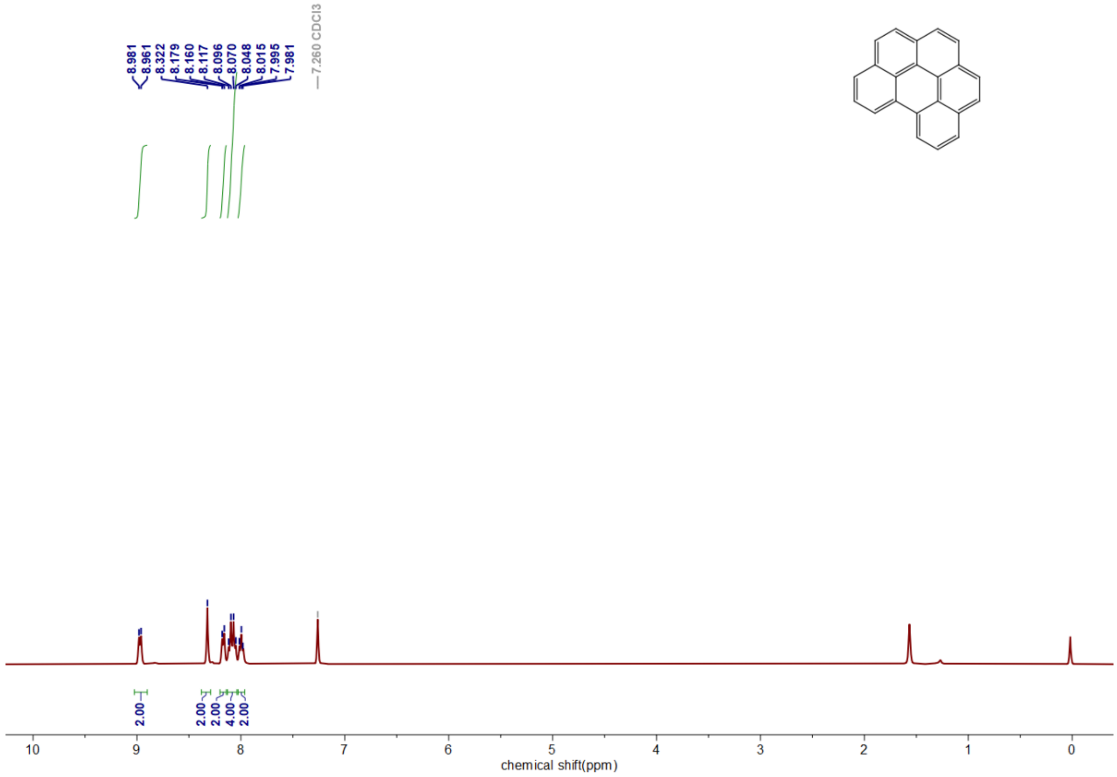

Figure S55. ^{1}H NMR of **BP** ($CDCl_3$, 400 MHz).

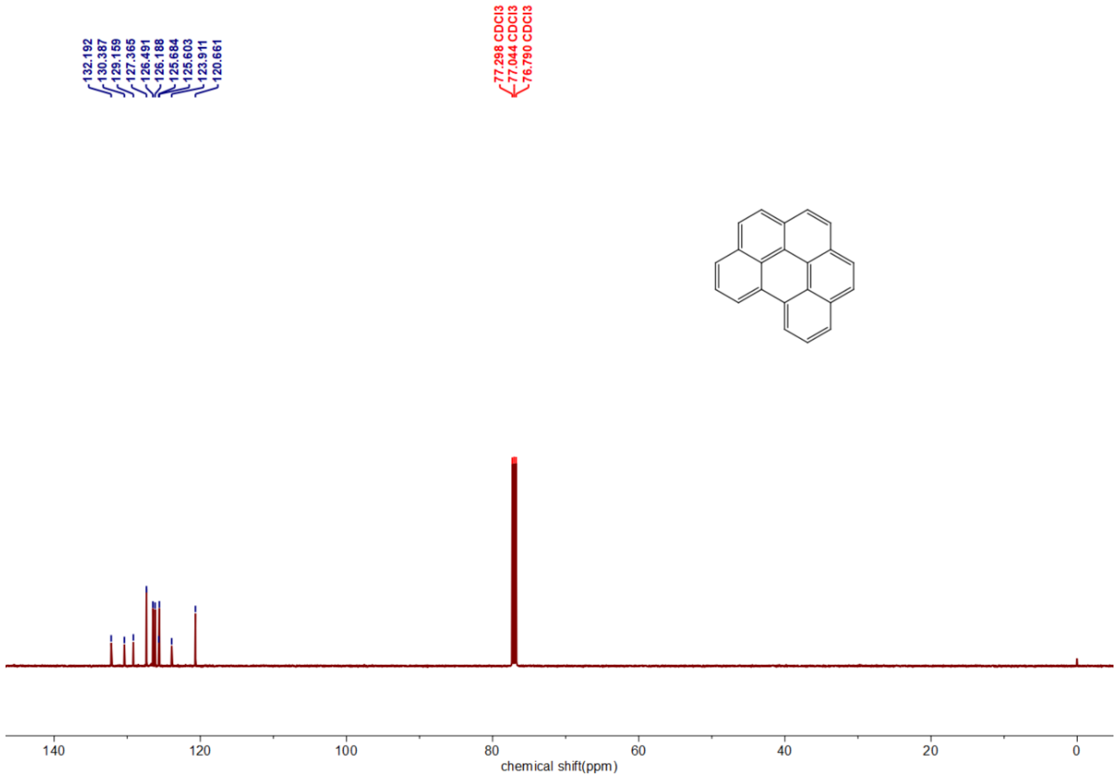

Figure S56. ^{13}C NMR of **BP** ($CDCl_3$, 126 MHz).

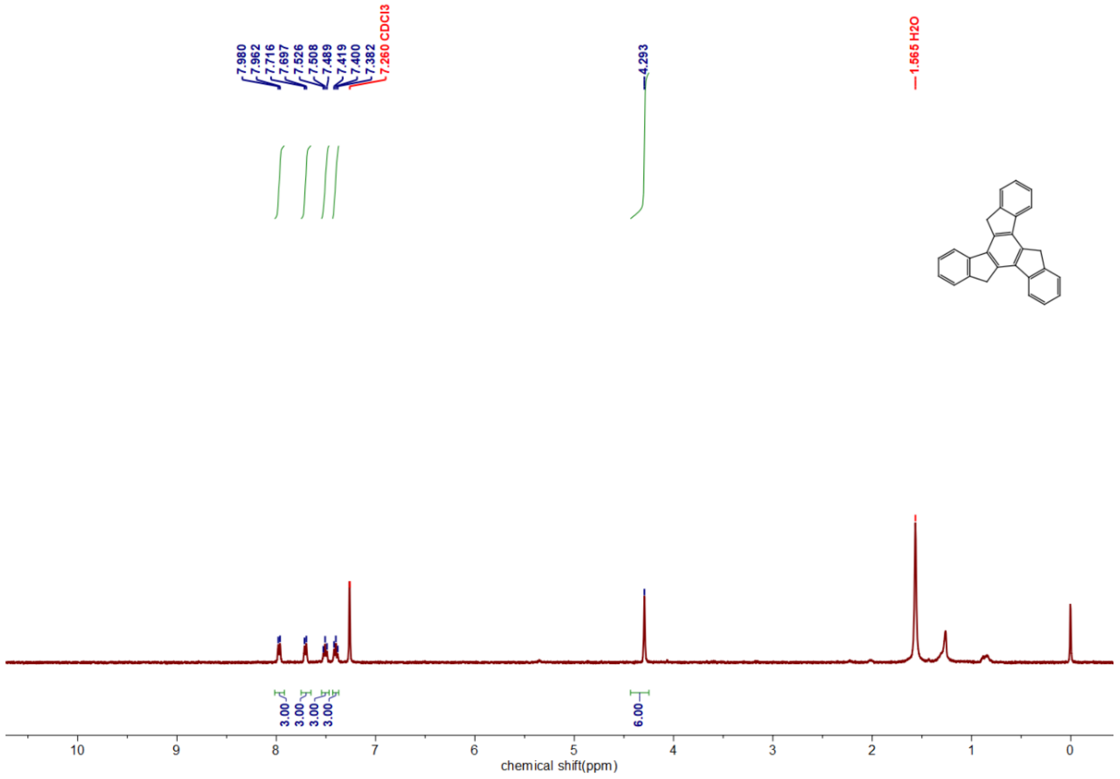

Figure S57. ^{1}H NMR of **Tur** ($CDCl_3$, 400 MHz).

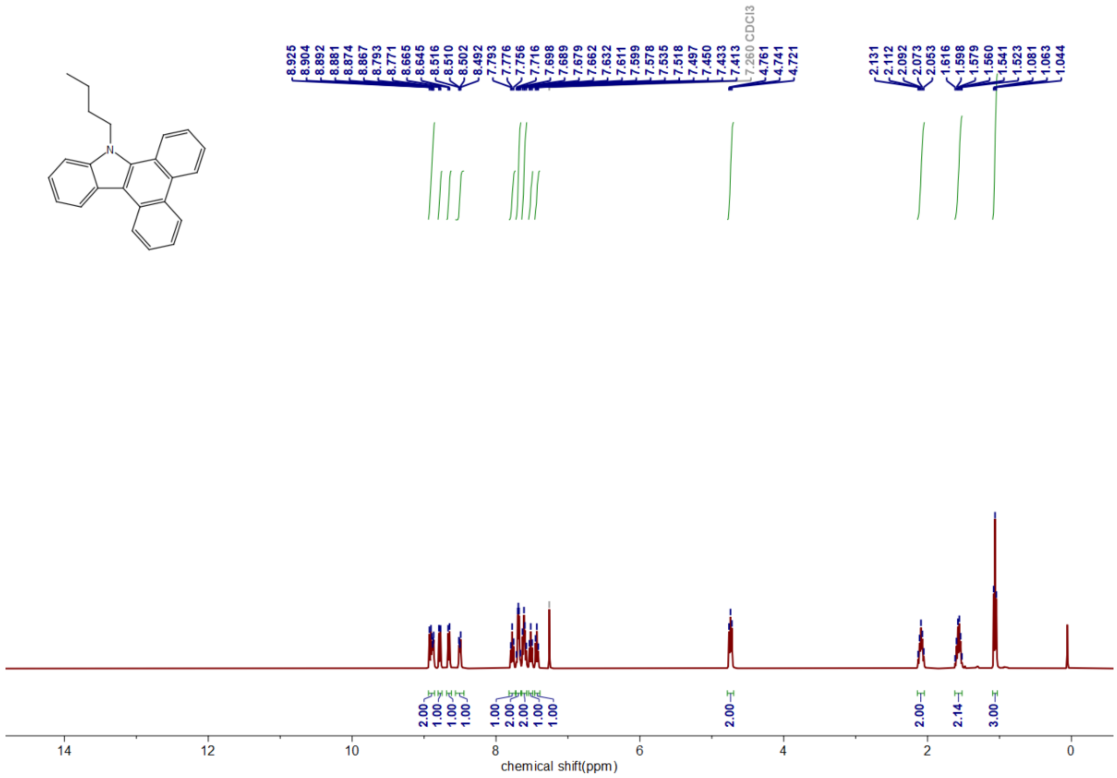

Figure S58. ^{1}H NMR of **BCZ-nBu** ($CDCl_3$, 400 MHz).

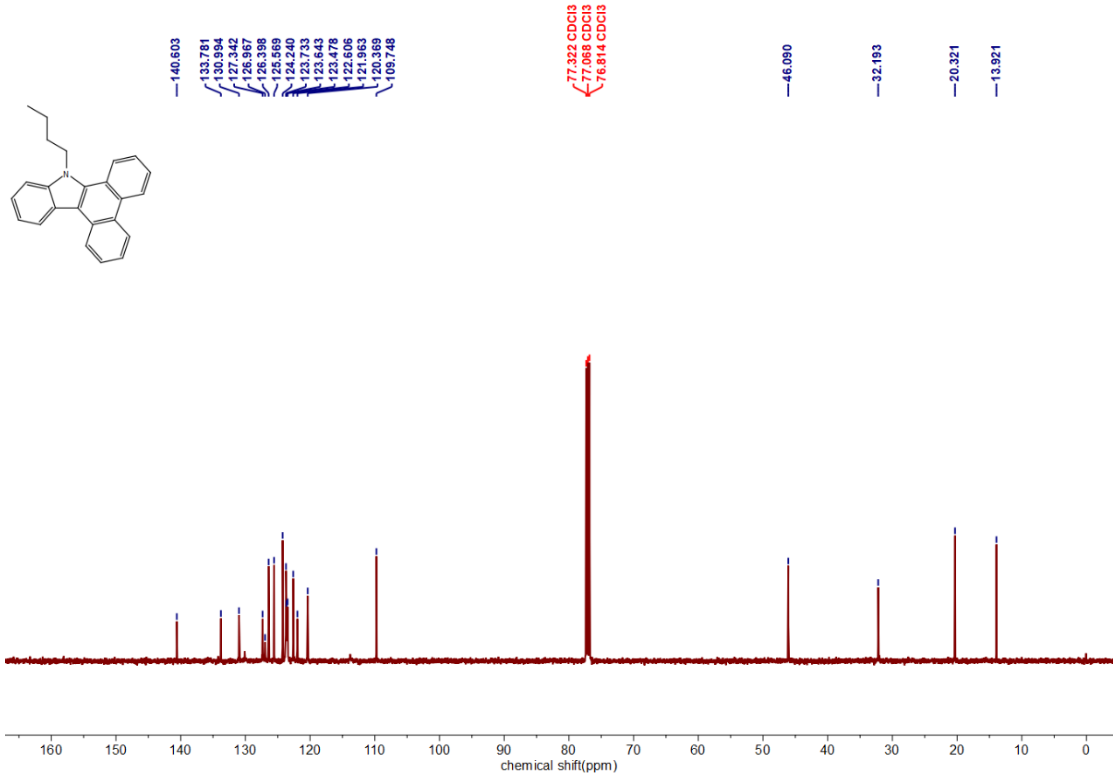

Figure S59. ^{13}C NMR of **BCZ-nBu** ($CDCl_3$, 126 MHz).

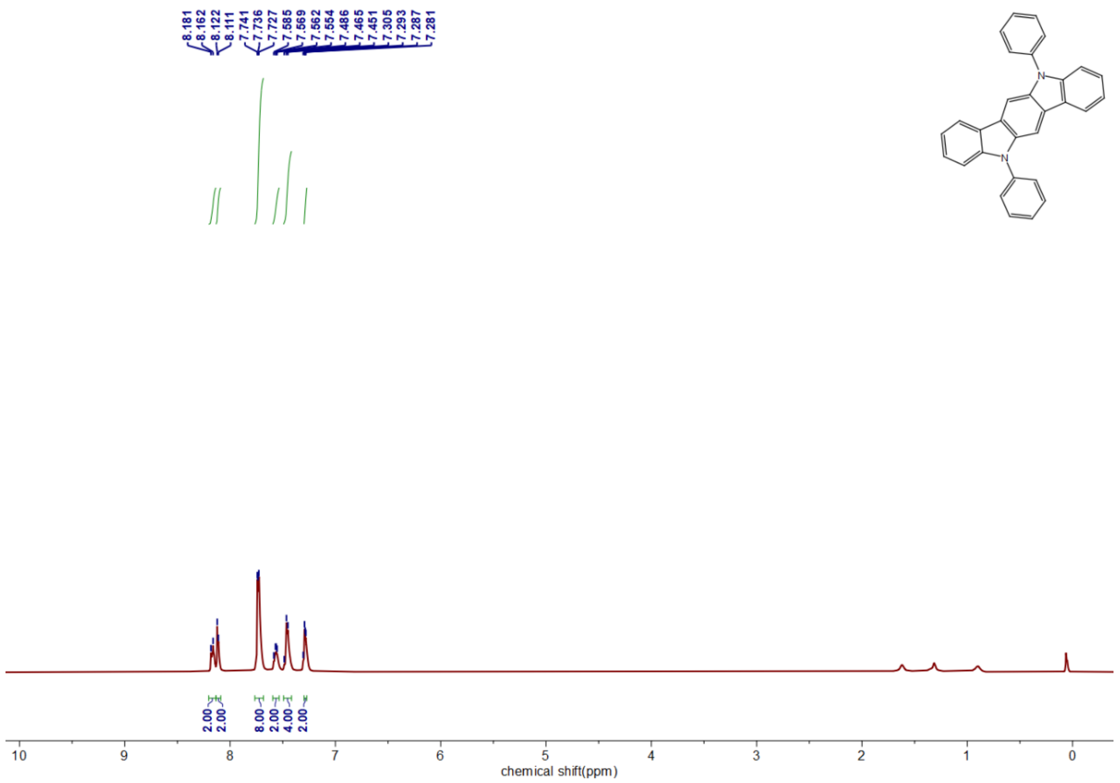

Figure S60. ^{1}H NMR of **IDBCZ** ($CDCl_3$, 400 MHz).

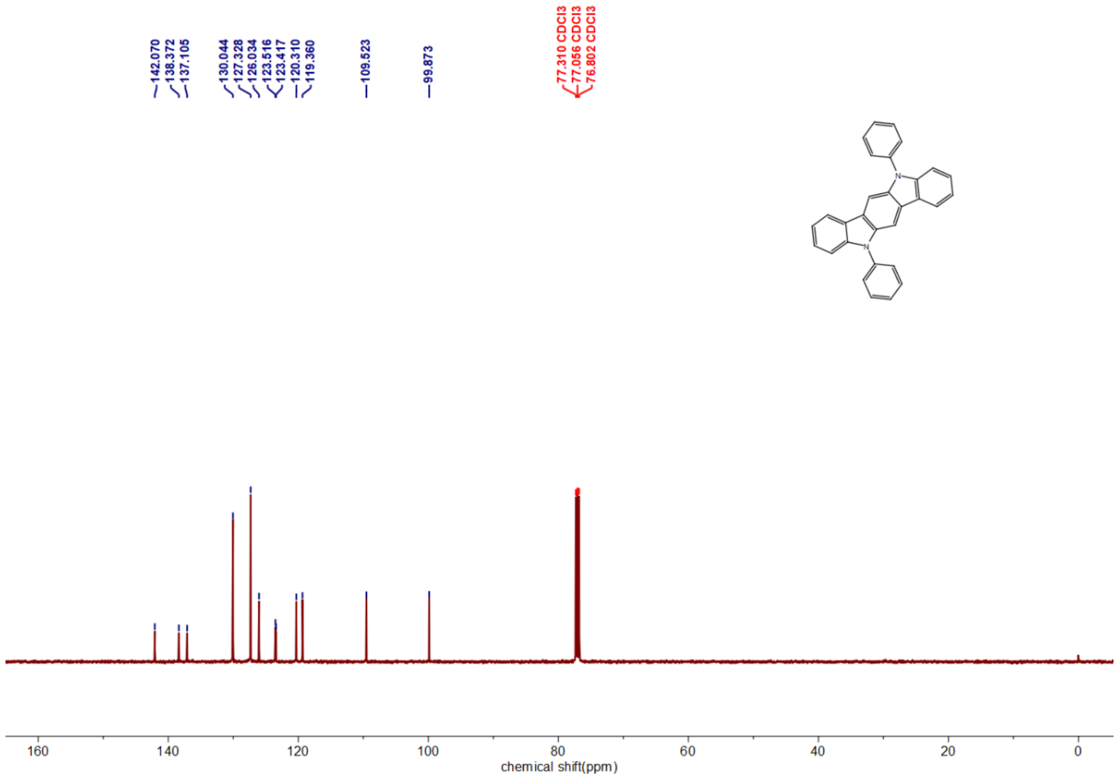

Figure S61. ^{13}C NMR of **IDBCZ** ($CDCl_3$, 126 MHz).

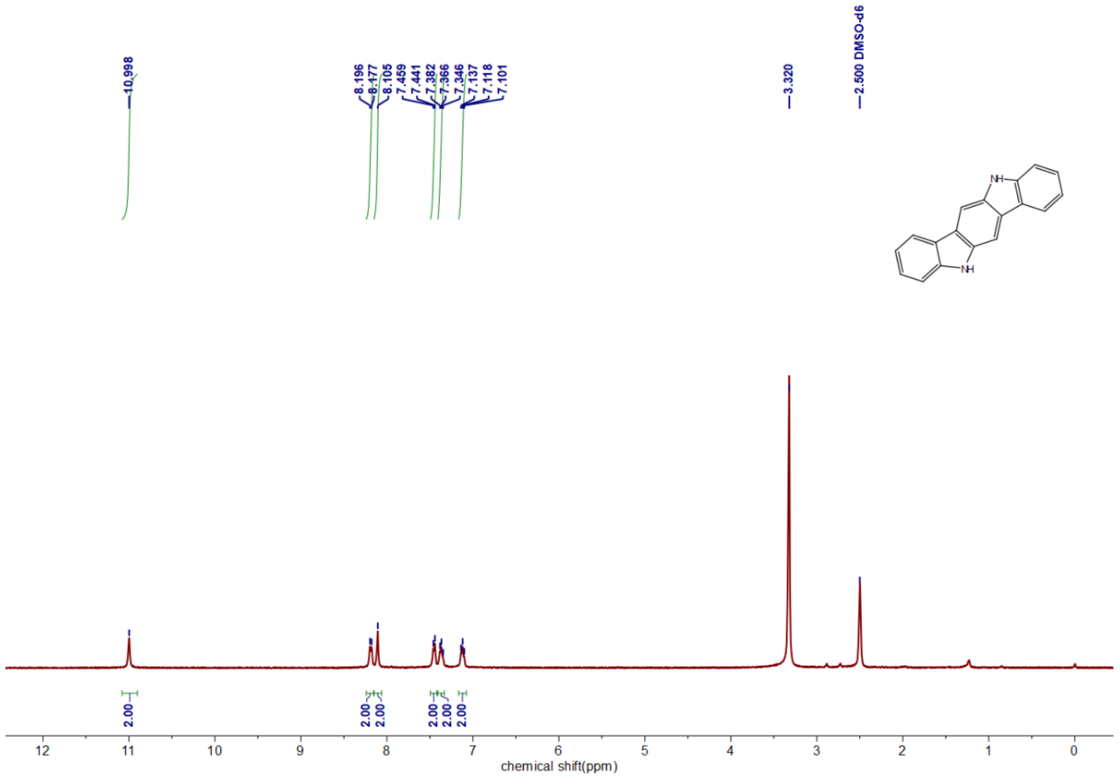

Figure S62. ^{1}H NMR of **HBCZ** (DMSO-*d6*, 400 MHz).

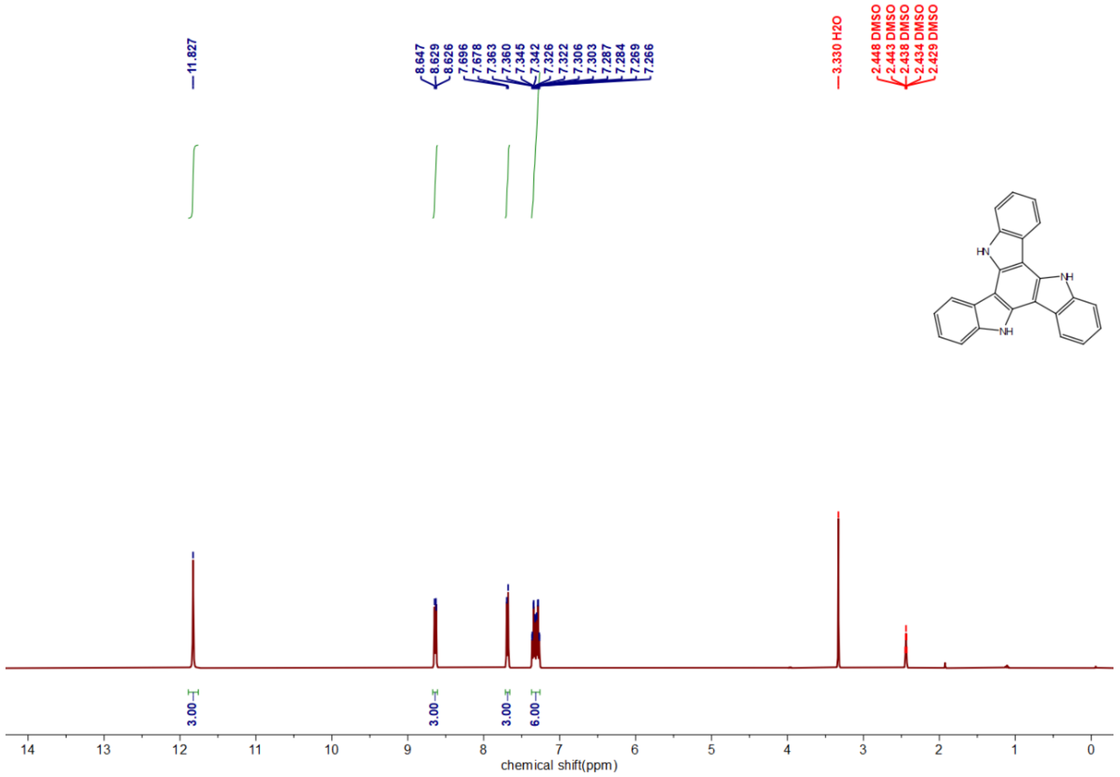

Figure S63. ^{1}H NMR of **HDBCZ** (DMSO-*d6*, 400 MHz).

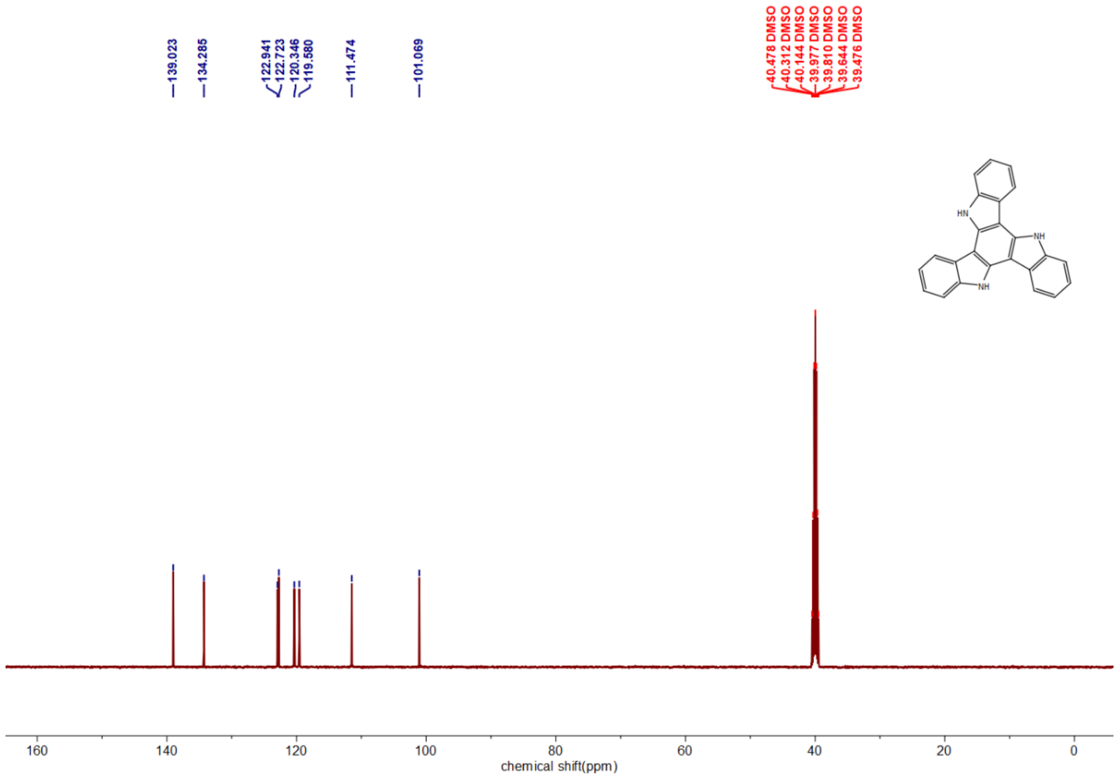

Figure S64. ^{13}C NMR of **HDBCZ** (DMSO-*d6*, 126 MHz).

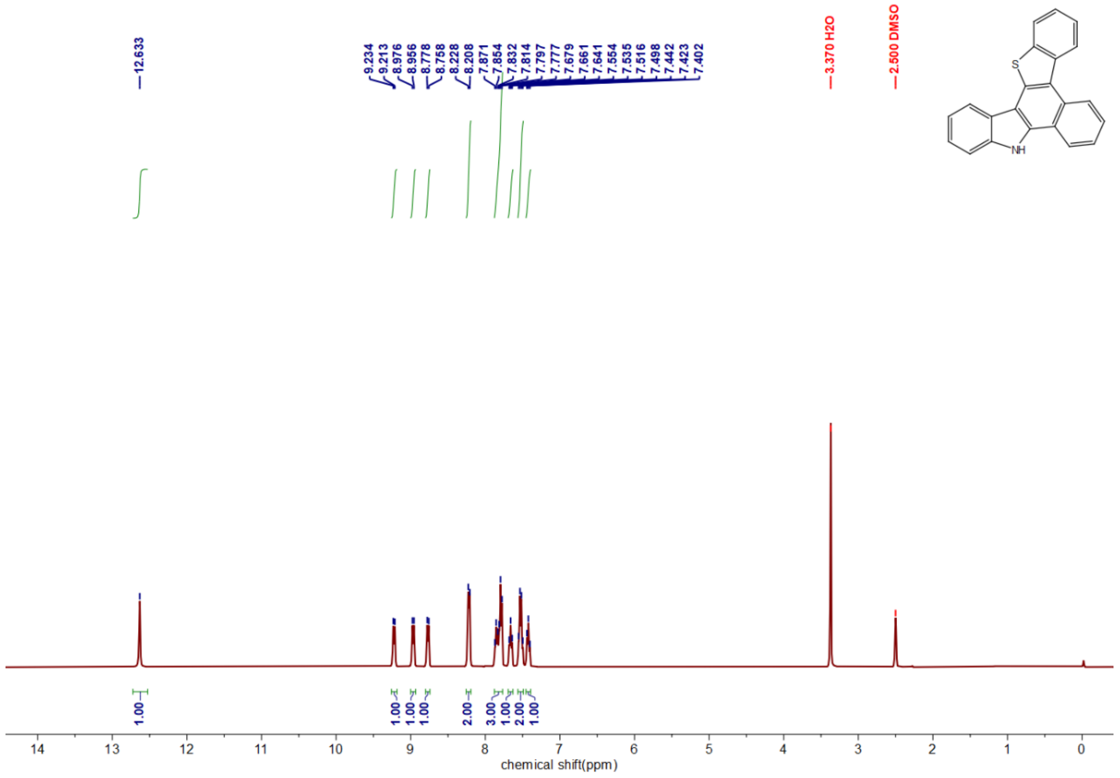

Figure S65. ^{1}H NMR of **BTBCZ** (DMSO-*d6*, 400 MHz).

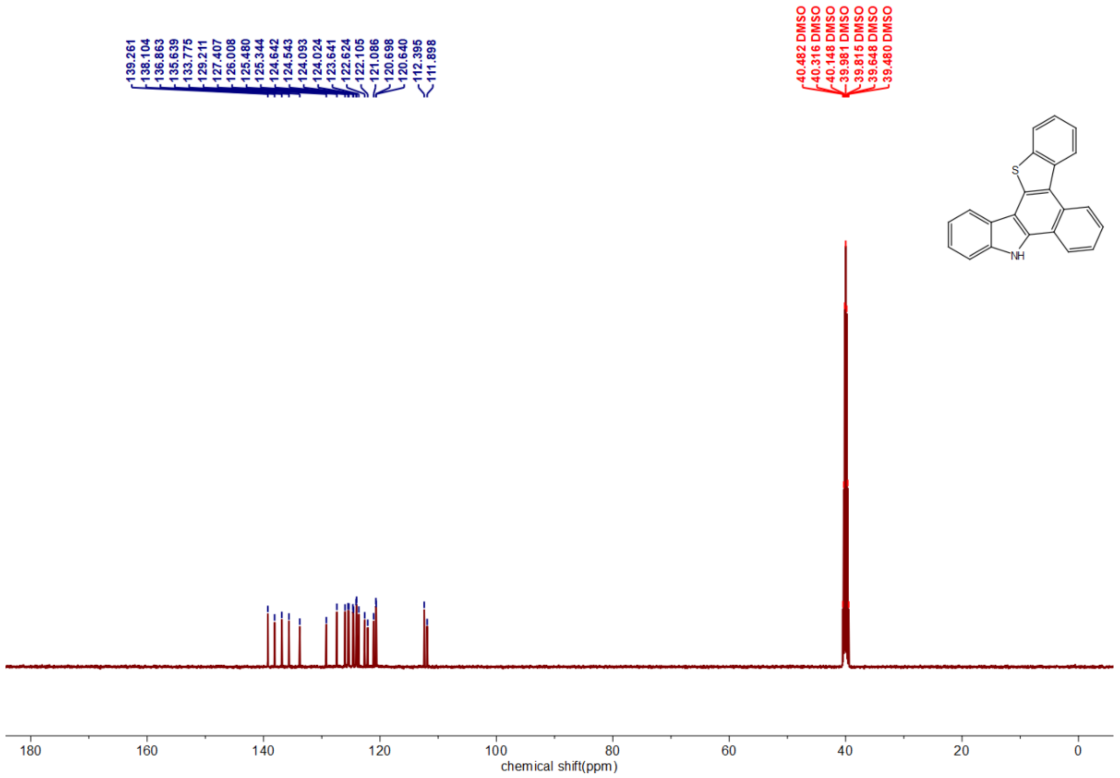

Figure S66. ^{13}C NMR of **BTBCZ** (DMSO-*d6*, 126 MHz).

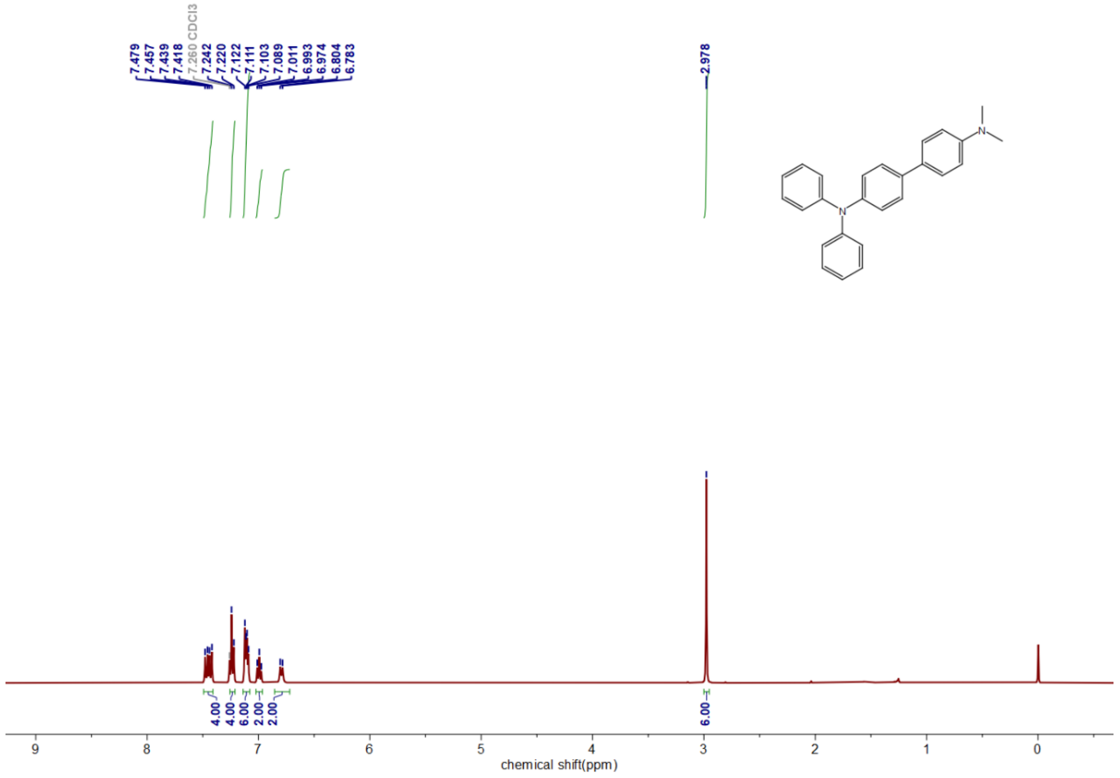

Figure S67. ^{1}H NMR of **MADBA** ($CDCl_3$, 400 MHz).

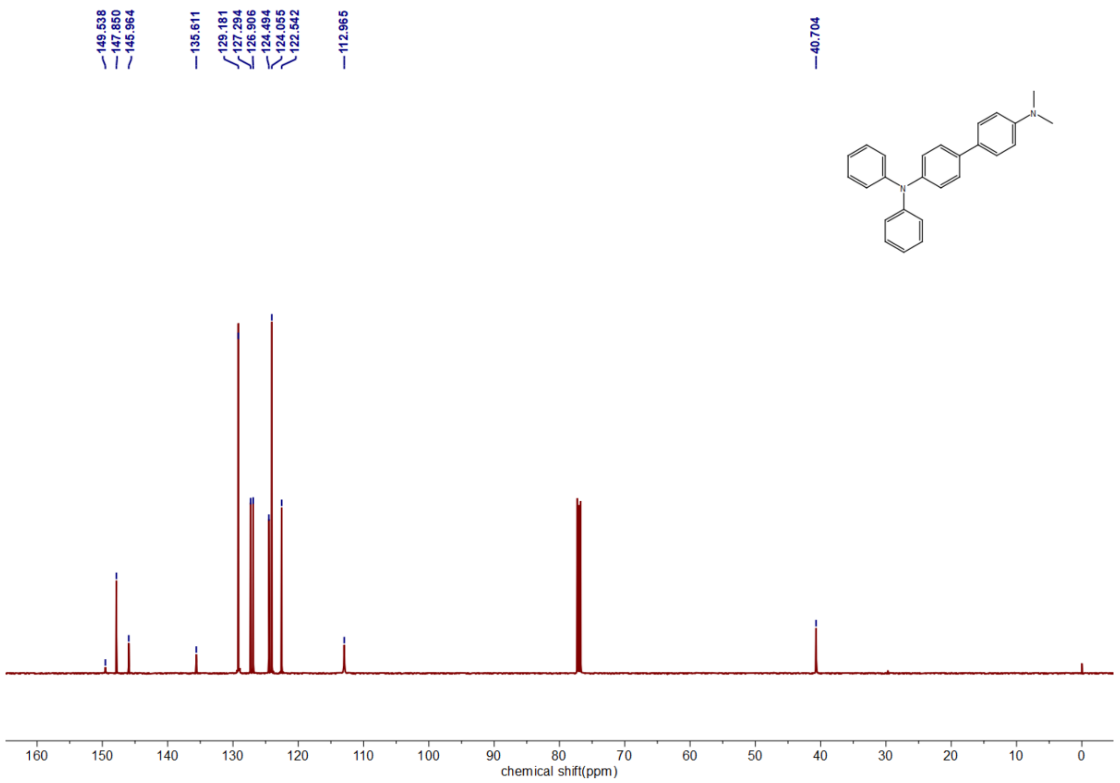

Figure S68. ^{13}C NMR of **MADBA** ($CDCl_3$, 126 MHz).

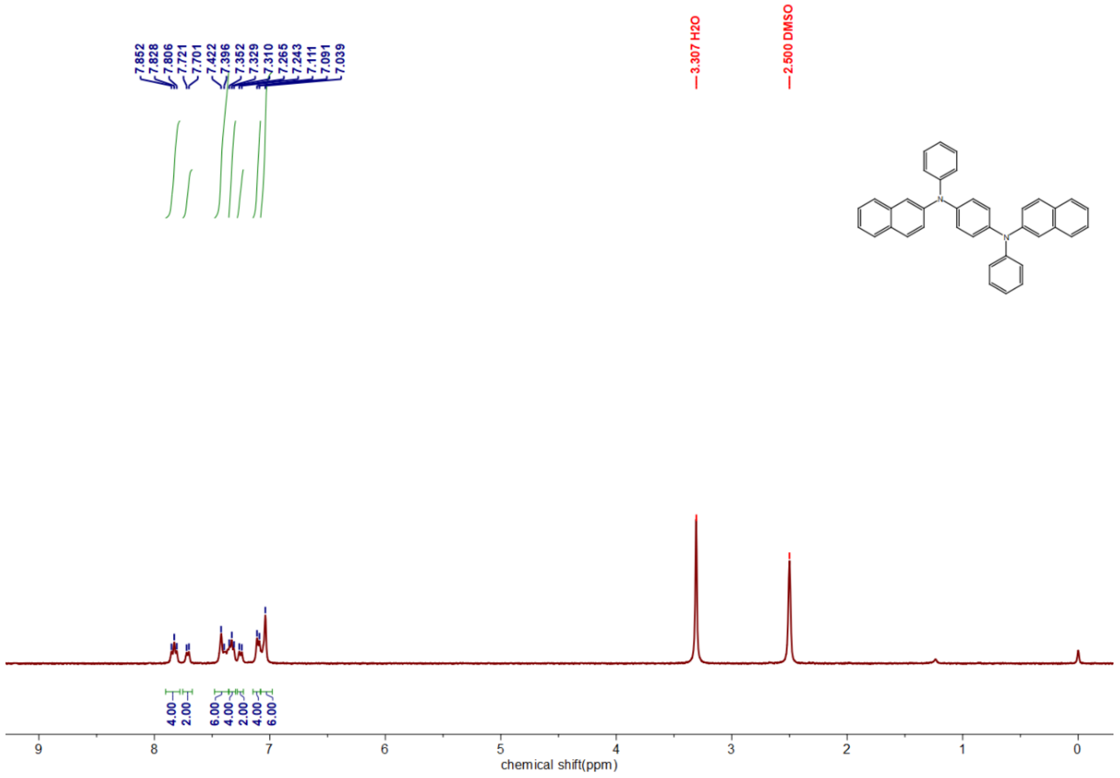

Figure S69. ^{1}H NMR of **BTPA** (DMSO-*d6*, 400 MHz).

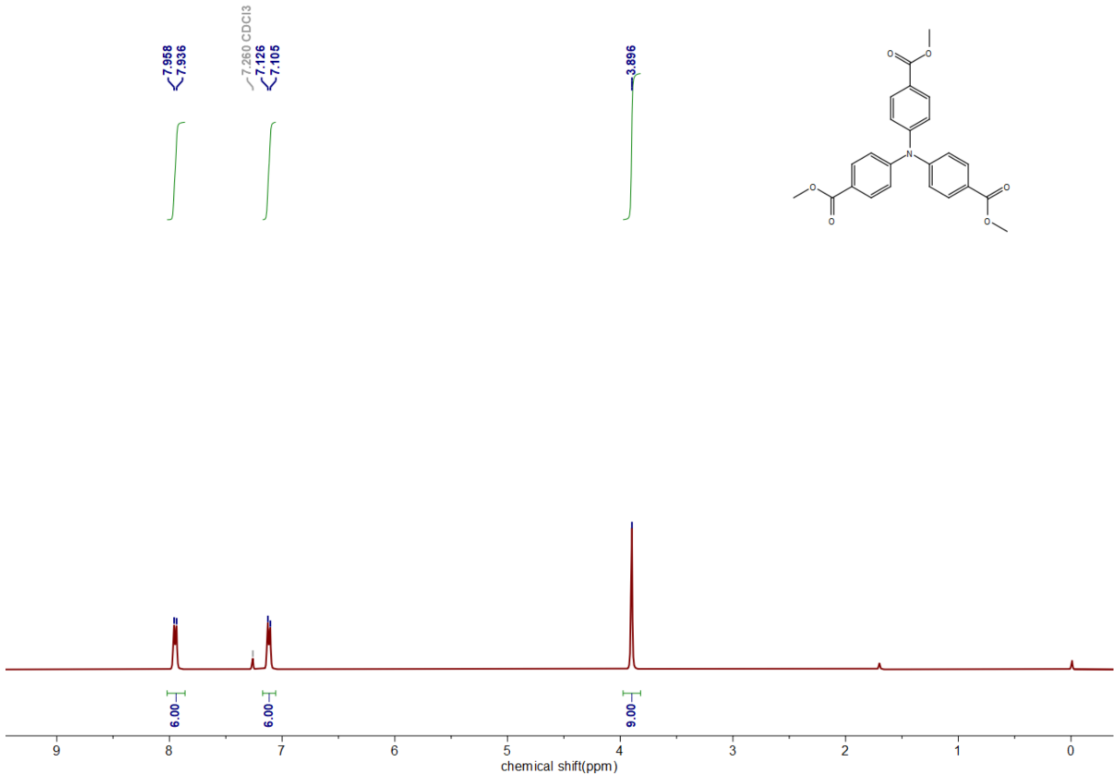

Figure S70. ^{1}H NMR of **TPAMA** ($CDCl_3$, 400 MHz).

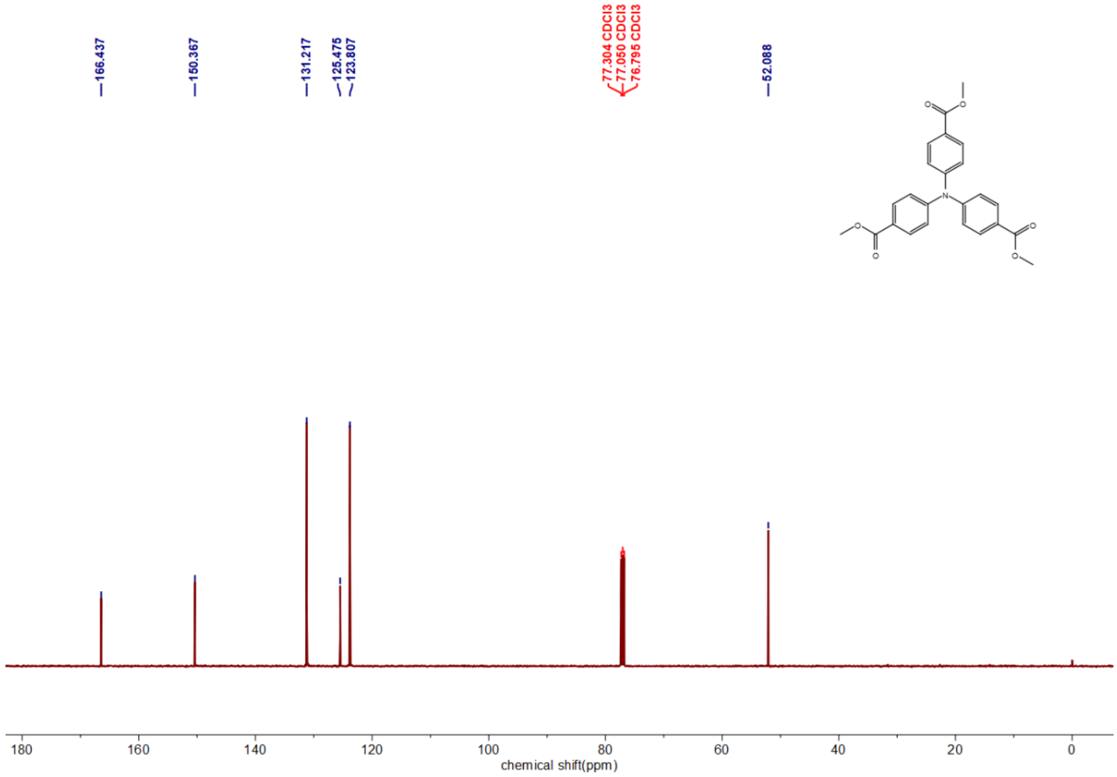

Figure S71. ^{13}C NMR of **TPAMA** ($CDCl_3$, 126 MHz).

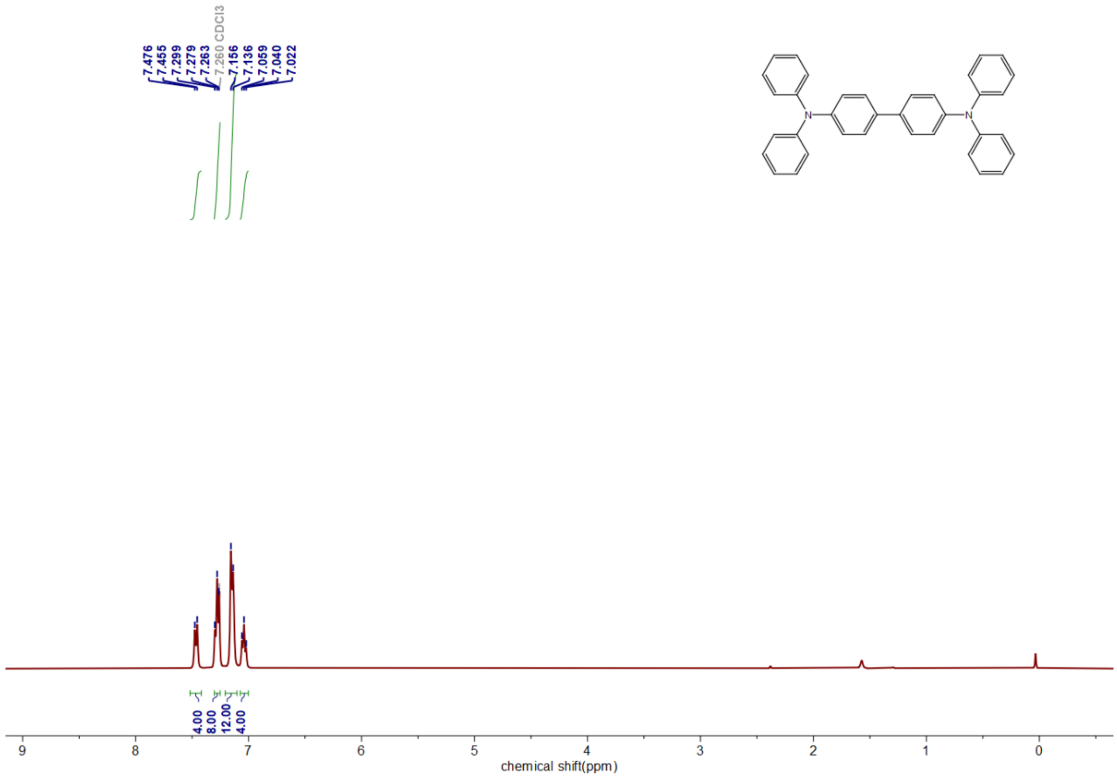

Figure S72. ^{1}H NMR of **2TPA** ($CDCl_3$, 400 MHz).

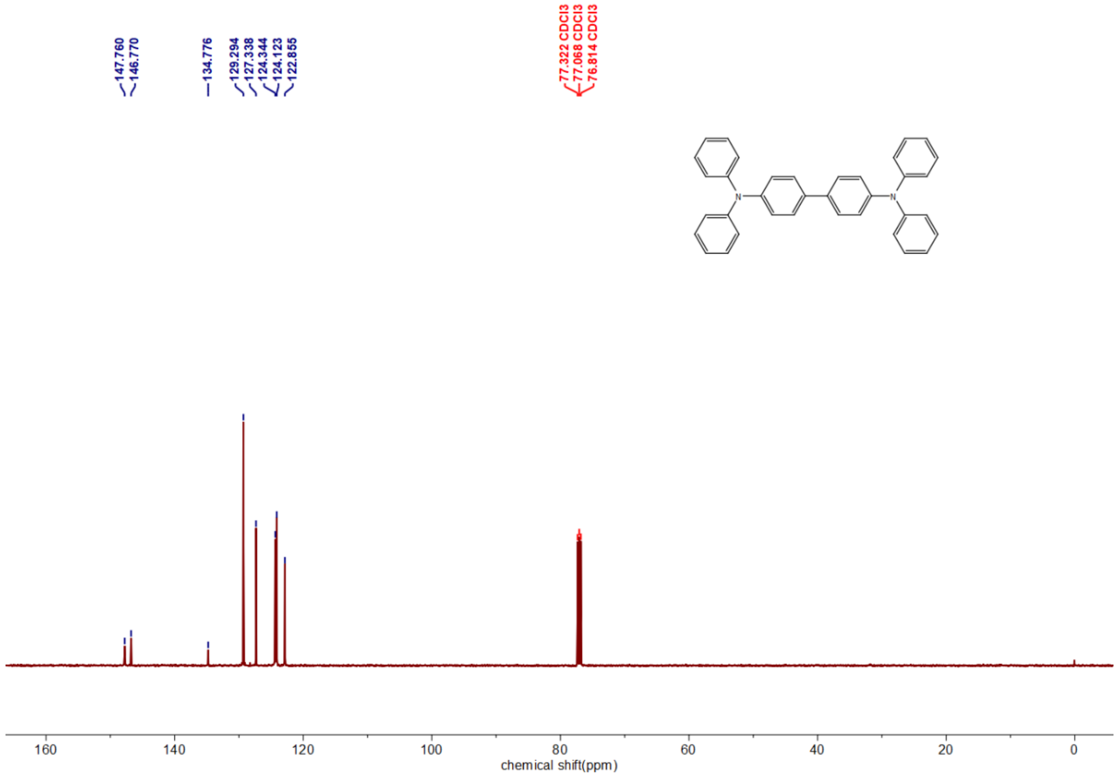

Figure S73. ^{13}C NMR of **2TPA** ($CDCl_3$, 126 MHz).

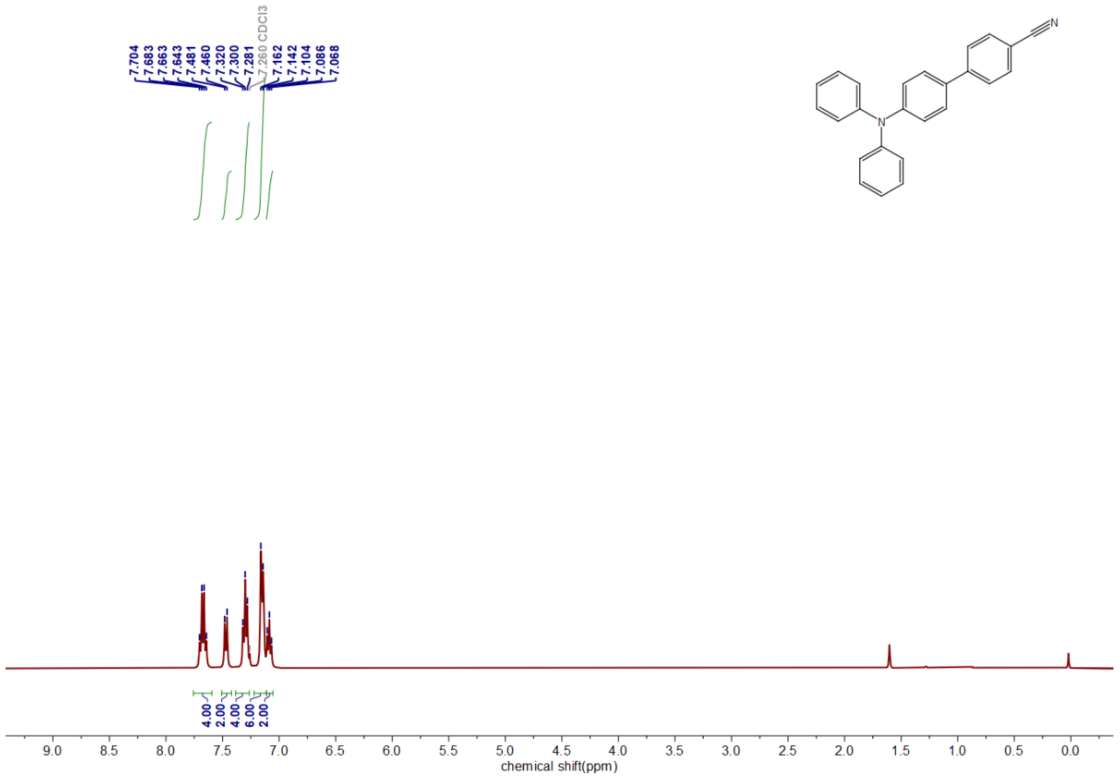

Figure S74. ^{1}H NMR of **DBC** ($CDCl_3$, 400 MHz).

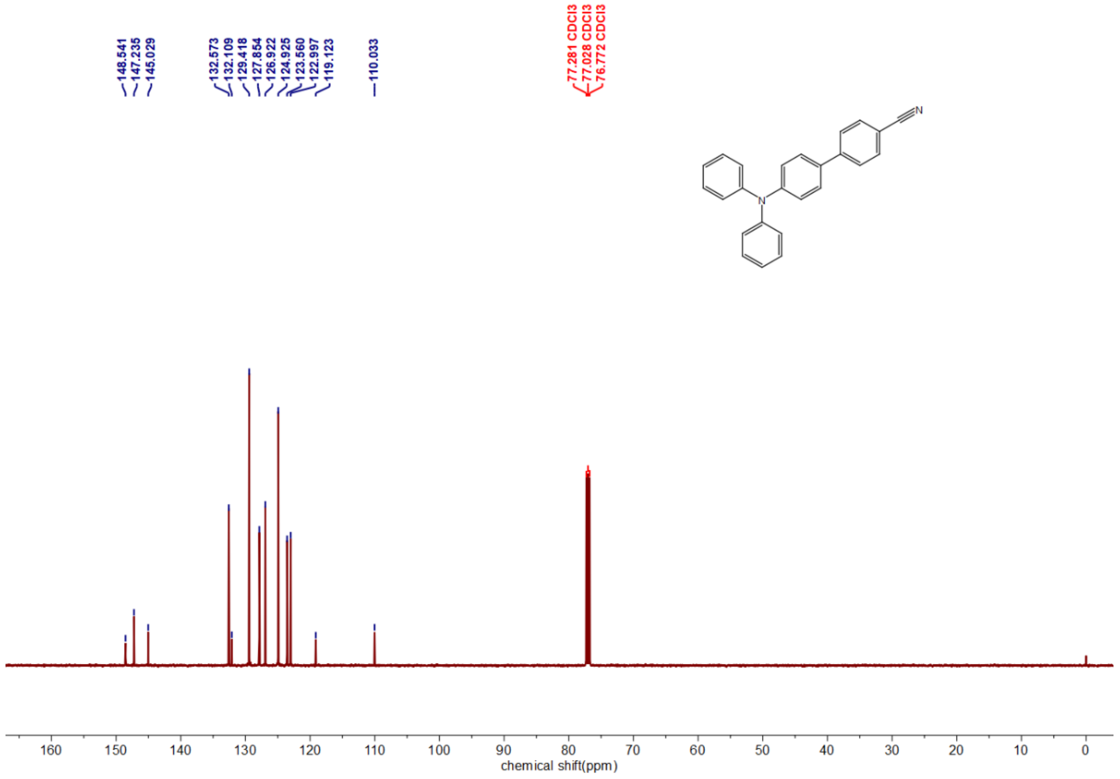

Figure S75. ^{13}C NMR of **DBC** ($CDCl_3$, 126 MHz).

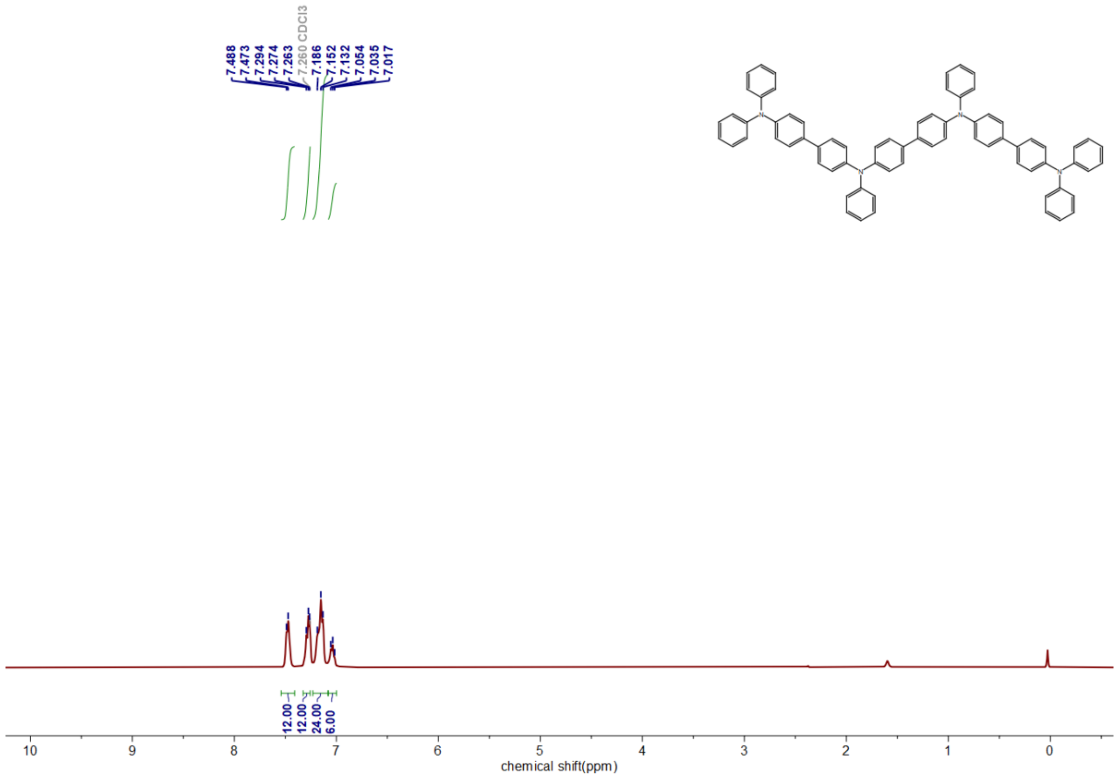

Figure S76. ^{1}H NMR of **4TPA** ($CDCl_3$, 400 MHz).

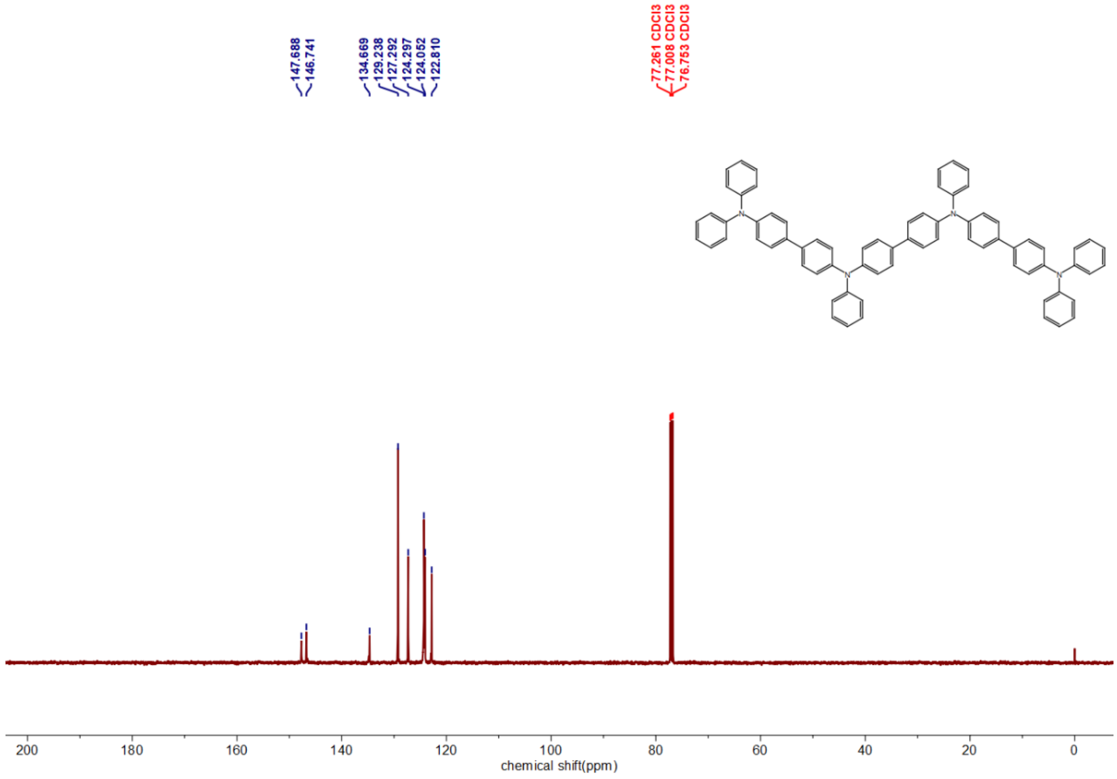

Figure S77. ^{13}C NMR of **4TPA** ($CDCl_3$, 126 MHz).

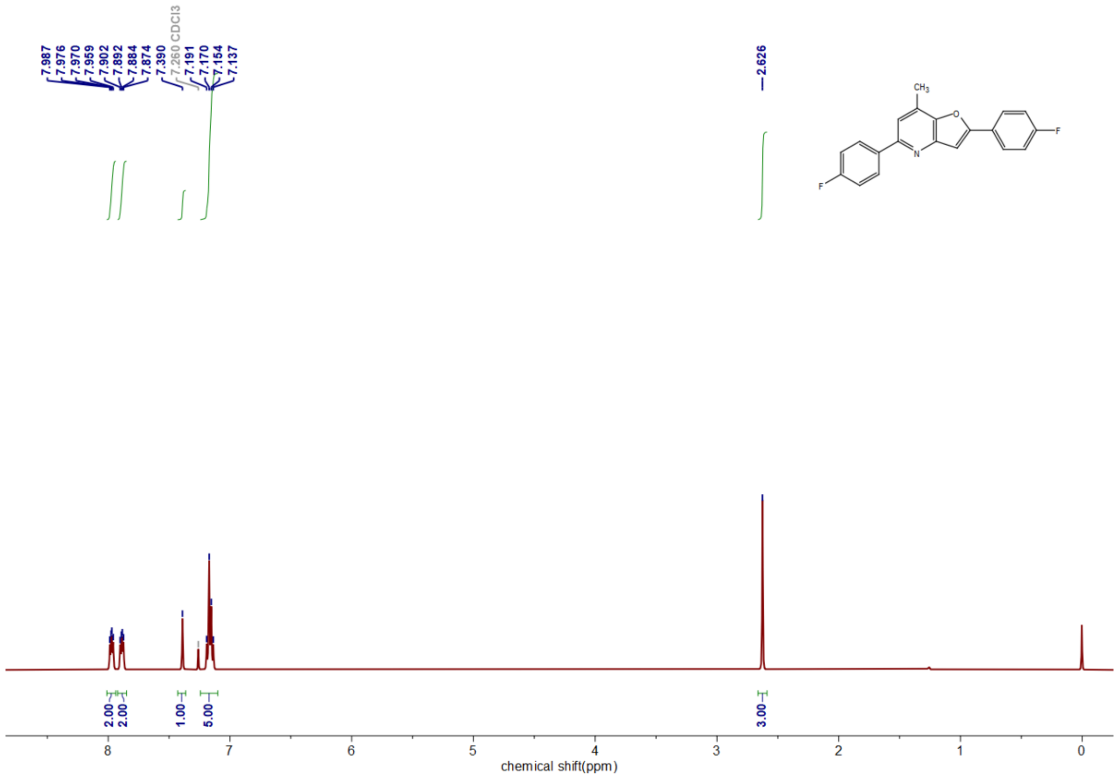

Figure S78. ^{1}H NMR of **PF-F** ($CDCl_3$, 500 MHz).

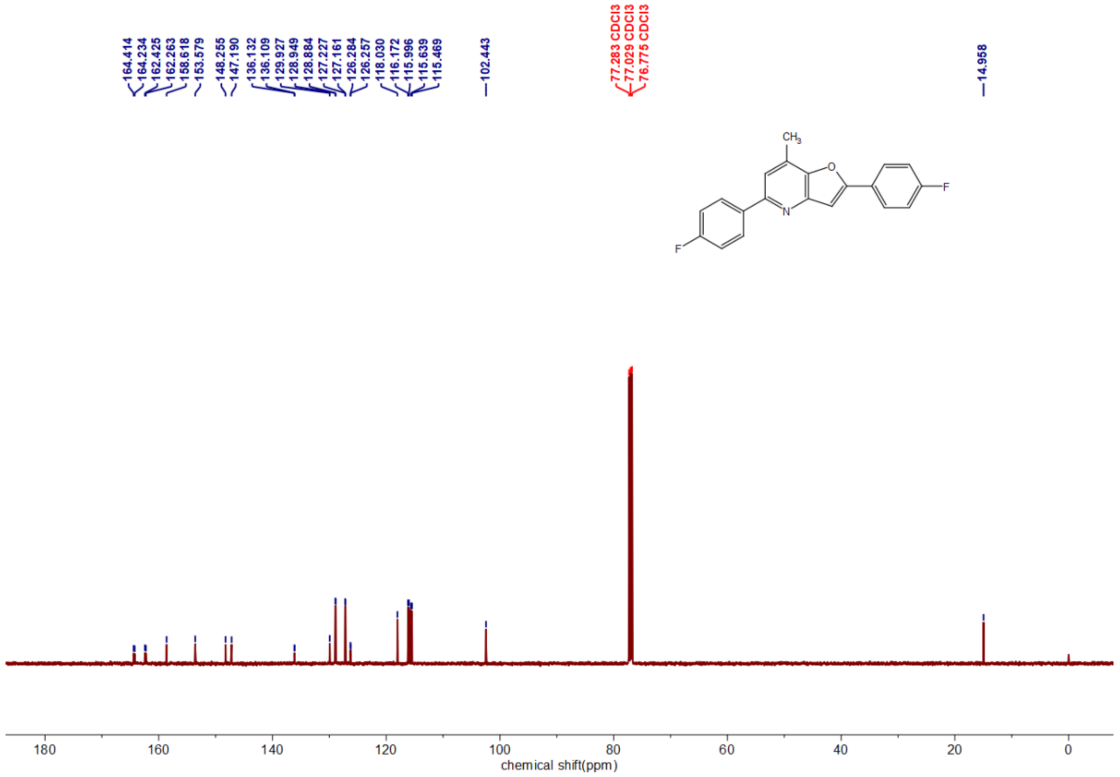

Figure S79. ^{13}C NMR of **PF-F** ($CDCl_3$, 126 MHz).

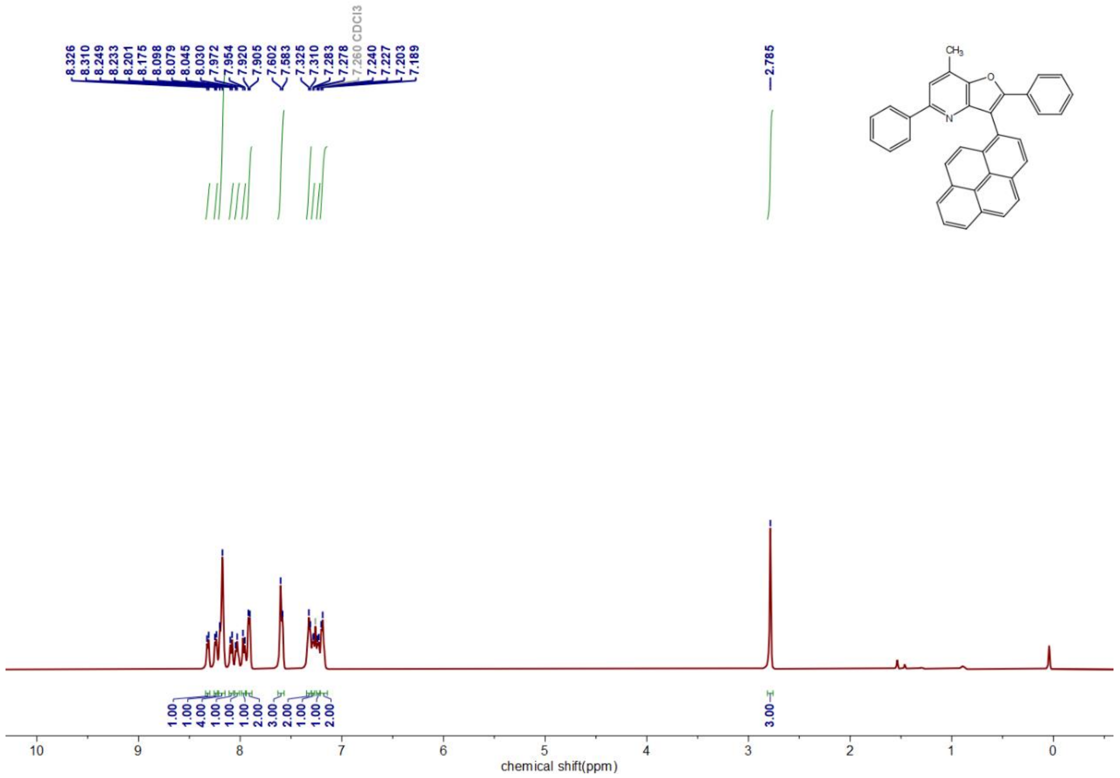

Figure S80. ^{1}H NMR of **PF-Py** ($CDCl_3$, 500 MHz).

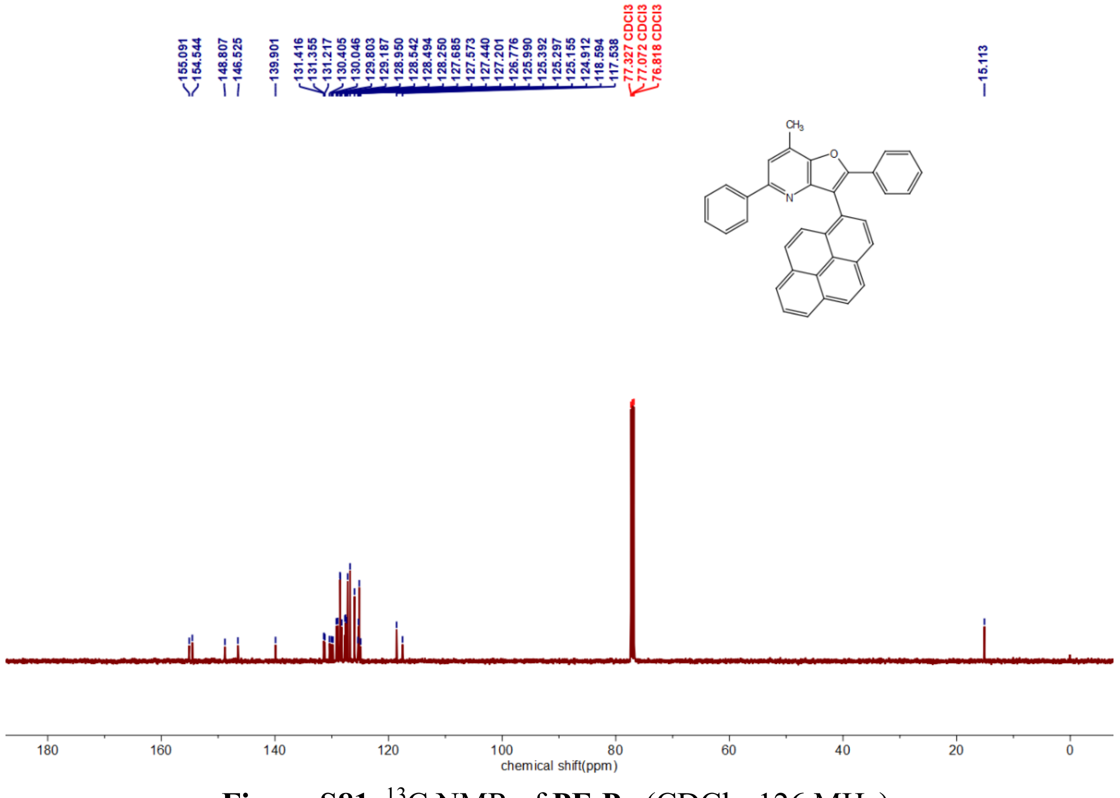

Figure S81. ^{13}C NMR of **PF-Py** ($CDCl_3$, 126 MHz).

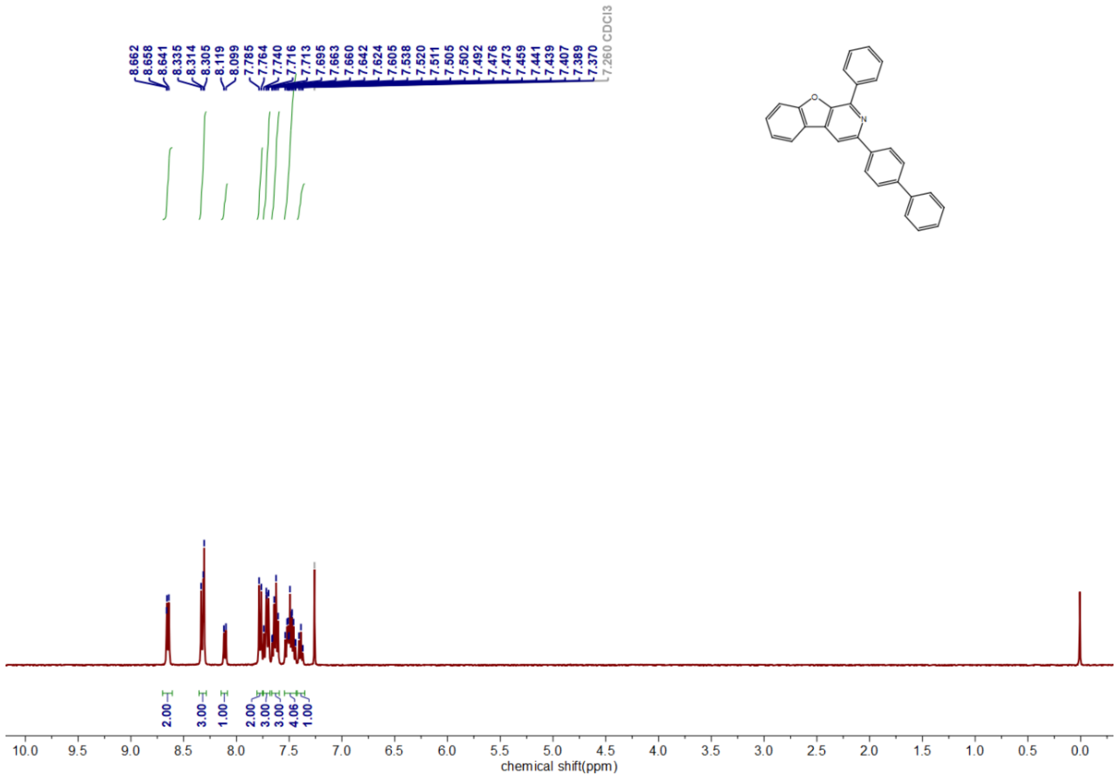

Figure S82. ^{1}H NMR of **3PhP** ($CDCl_3$, 400 MHz).

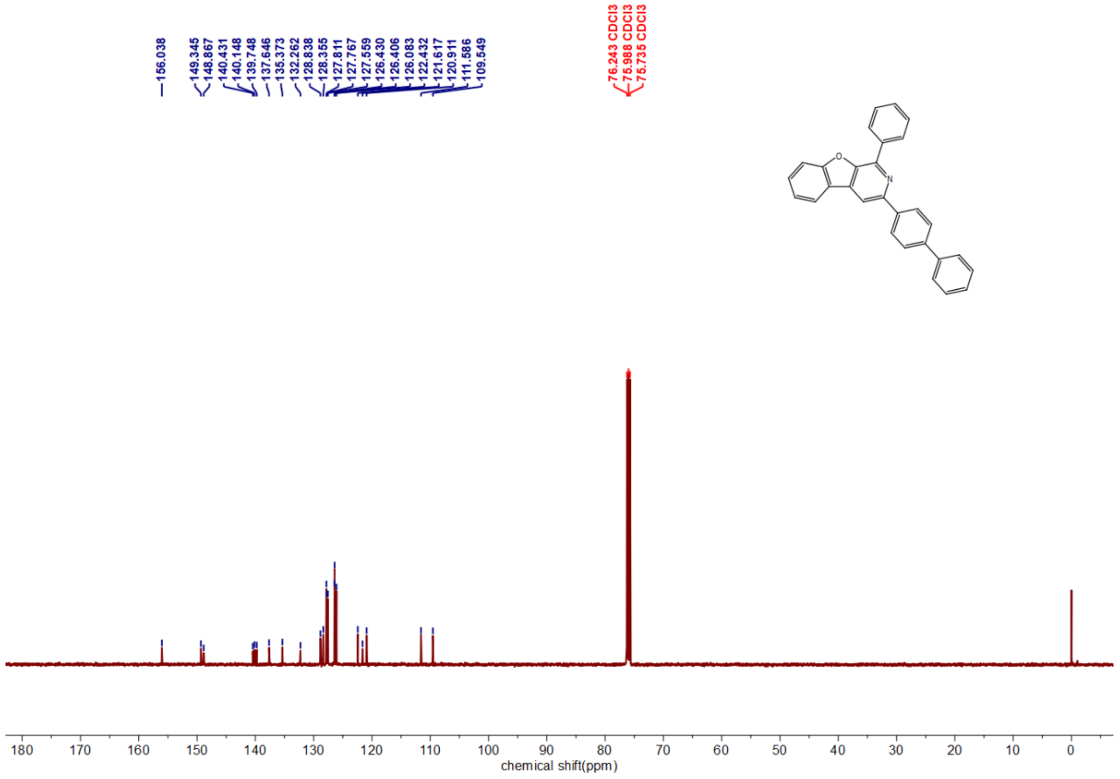

Figure S83. ^{13}C NMR of **3PhP** ($CDCl_3$, 126 MHz).

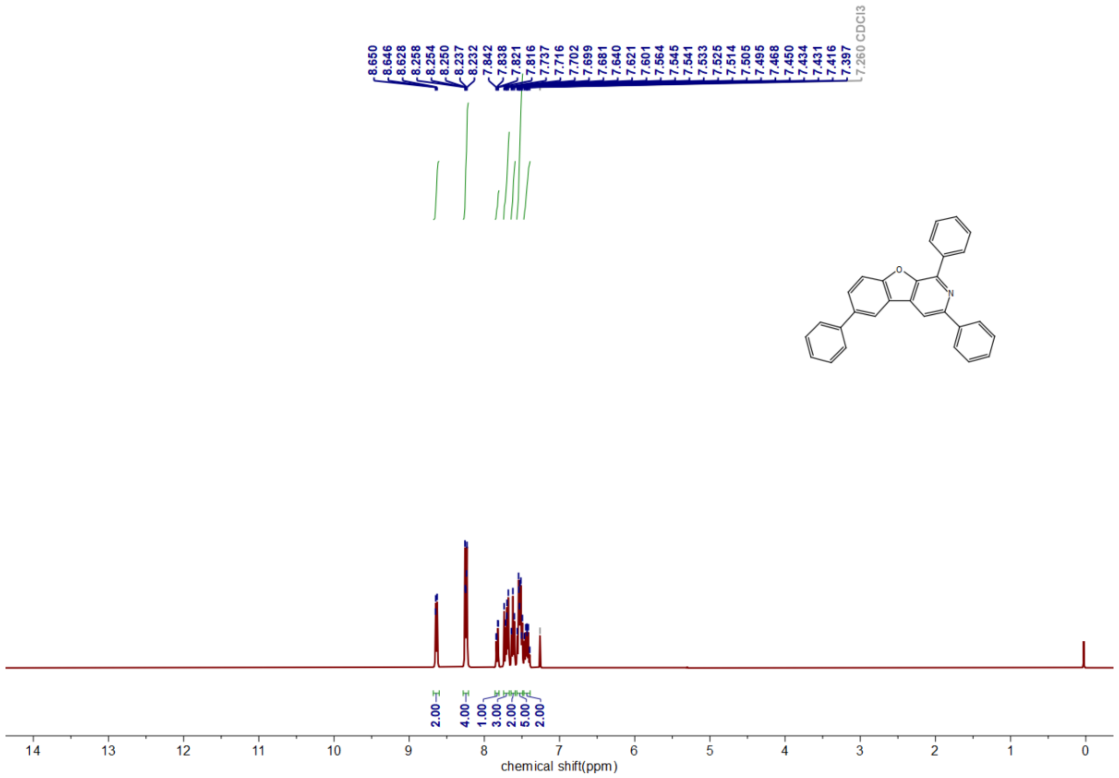

Figure S84. ^{1}H NMR of **6PhP** ($CDCl_3$, 400 MHz).

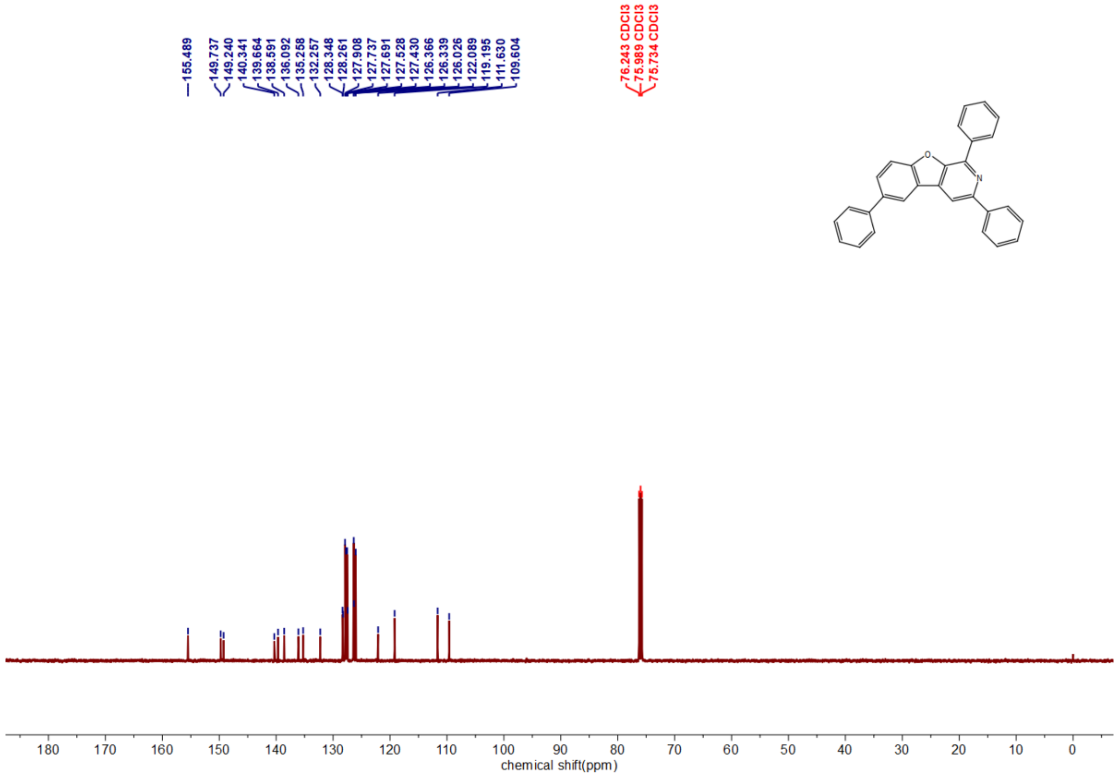

Figure S85. ^{13}C NMR of **6PhP** ($CDCl_3$, 126 MHz).

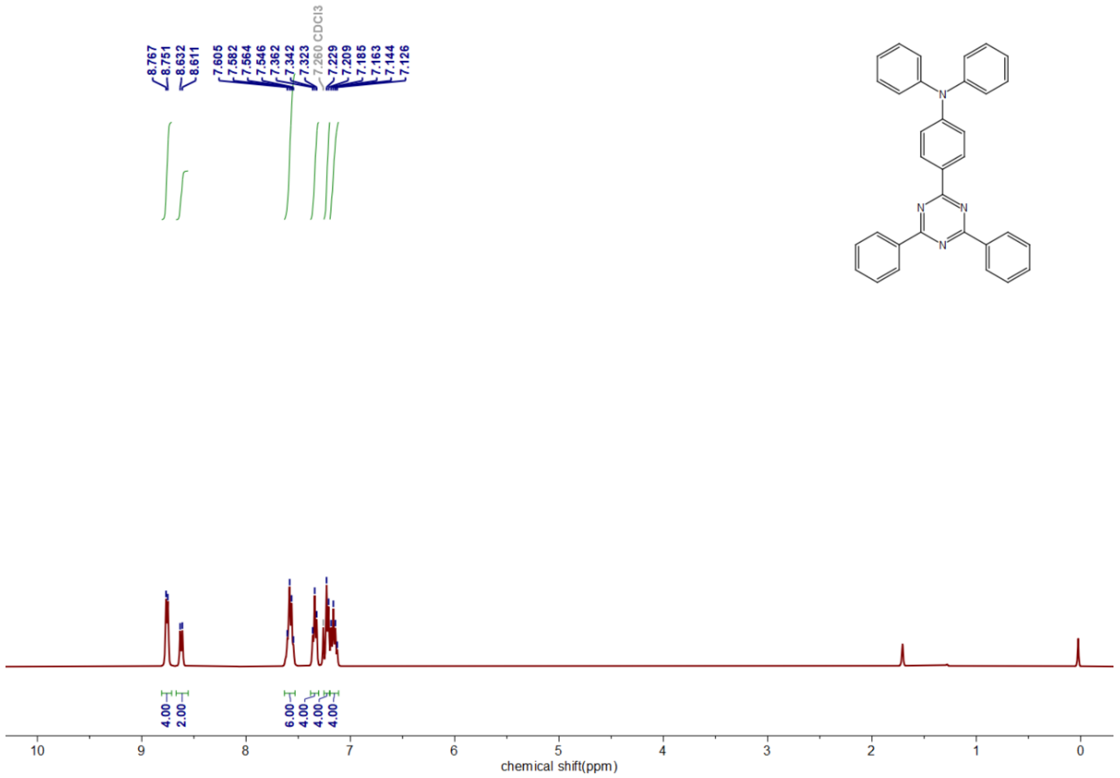

Figure S86. ^{1}H NMR of **TRZ-1** ($CDCl_3$, 400 MHz).

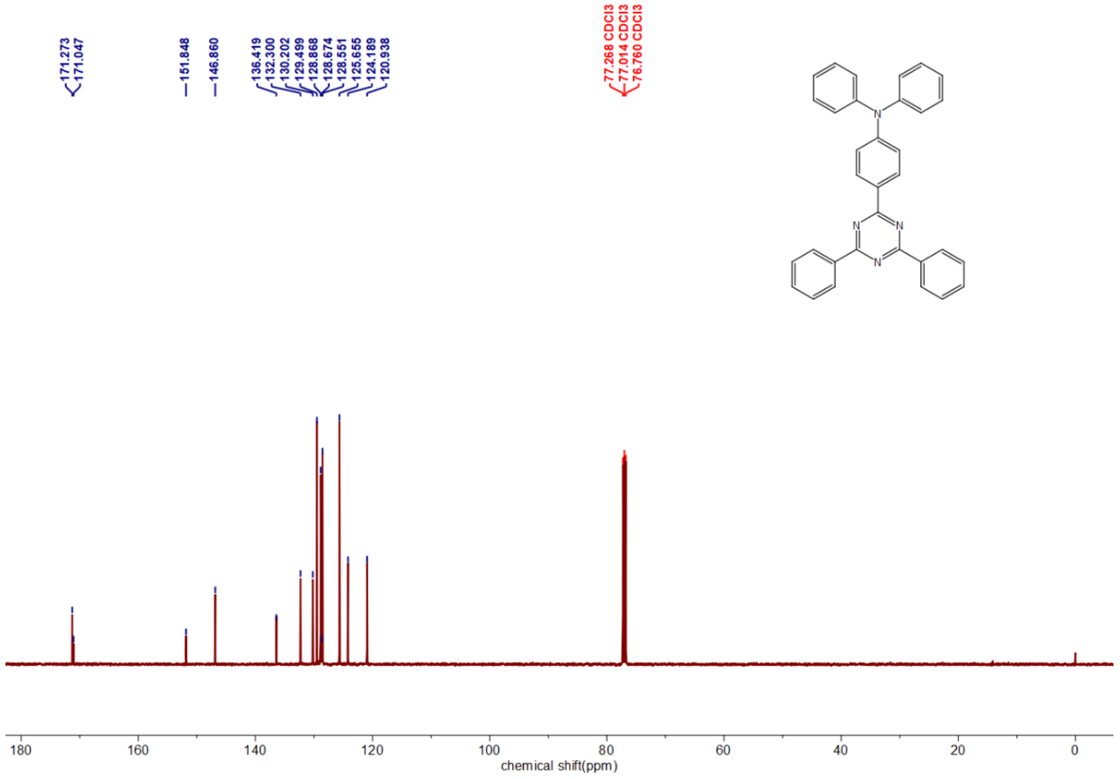

Figure S87. ^{13}C NMR of **TRZ-1** ($CDCl_3$, 126 MHz).

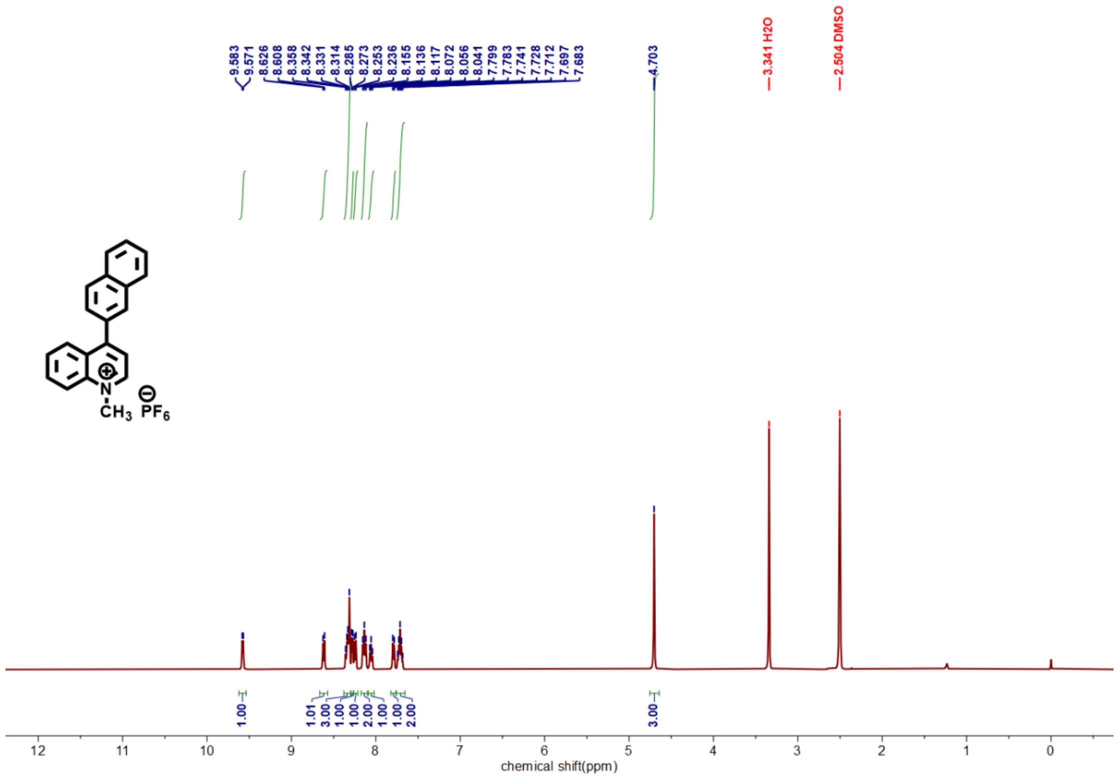

Figure S88. ^{1}H NMR of **QL-A** (DMSO-*d6*, 500 MHz).

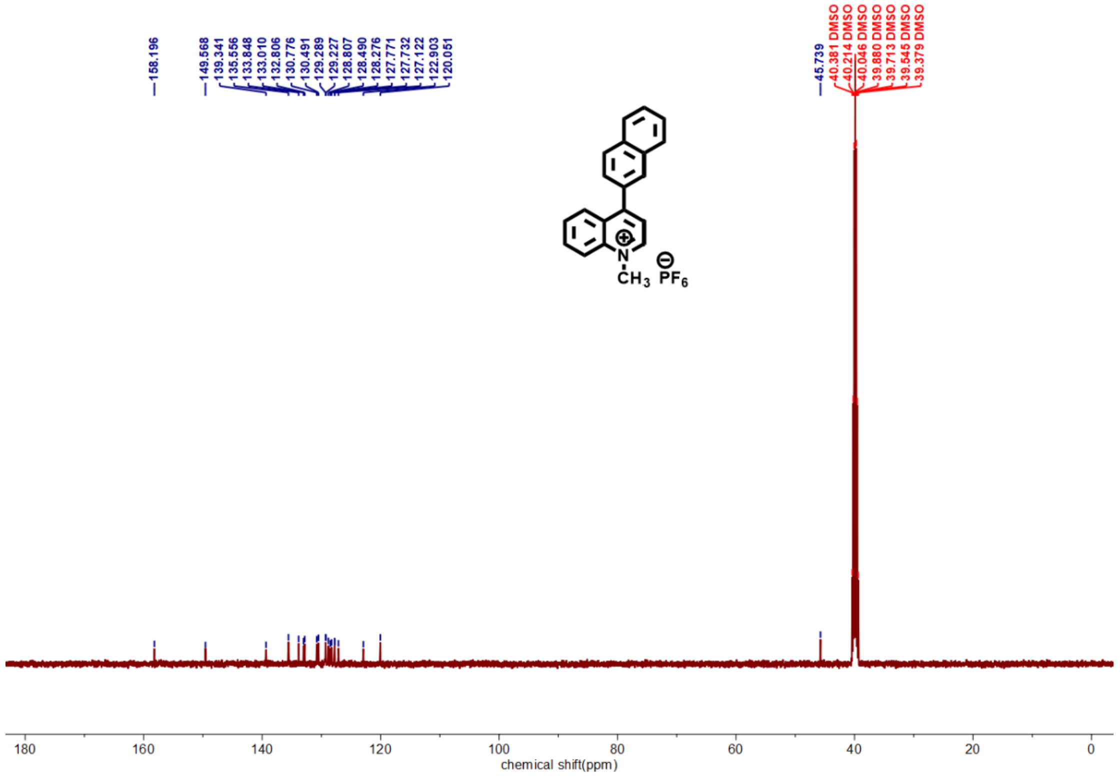

Figure S89. ^{13}C NMR of **QL-A** (DMSO-*d6*, 126 MHz).

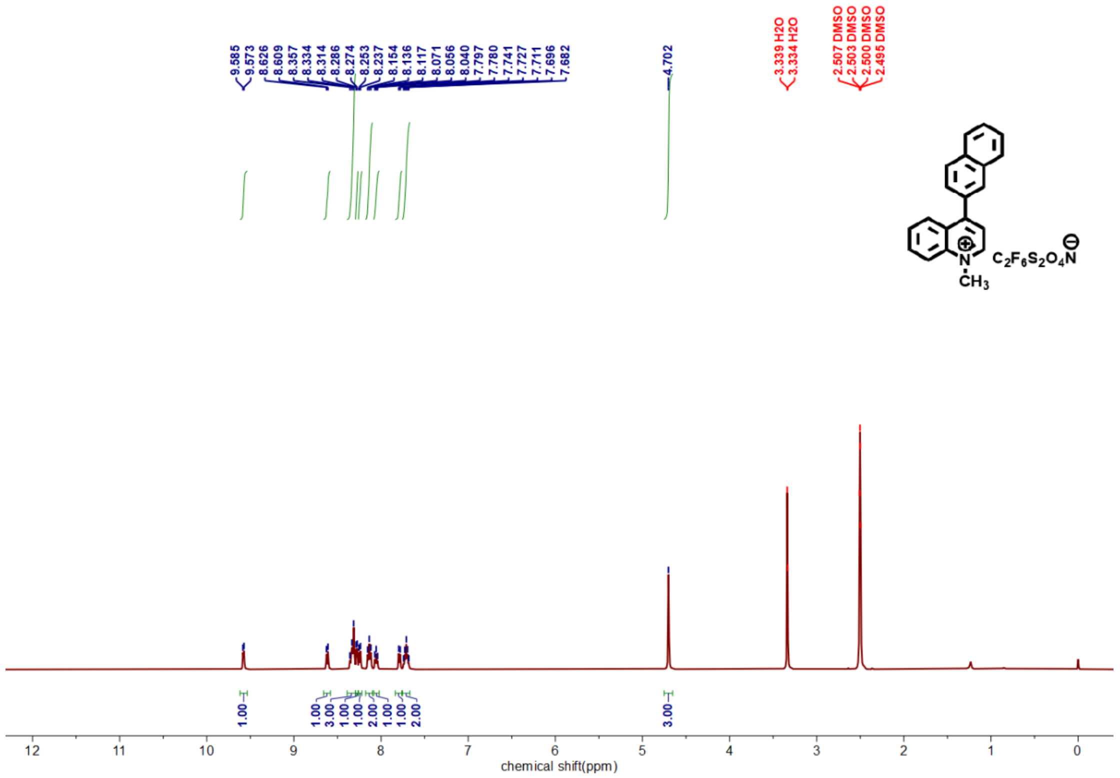

Figure S90. ^{1}H NMR of **QL-B** (DMSO-*d6*, 500 MHz).

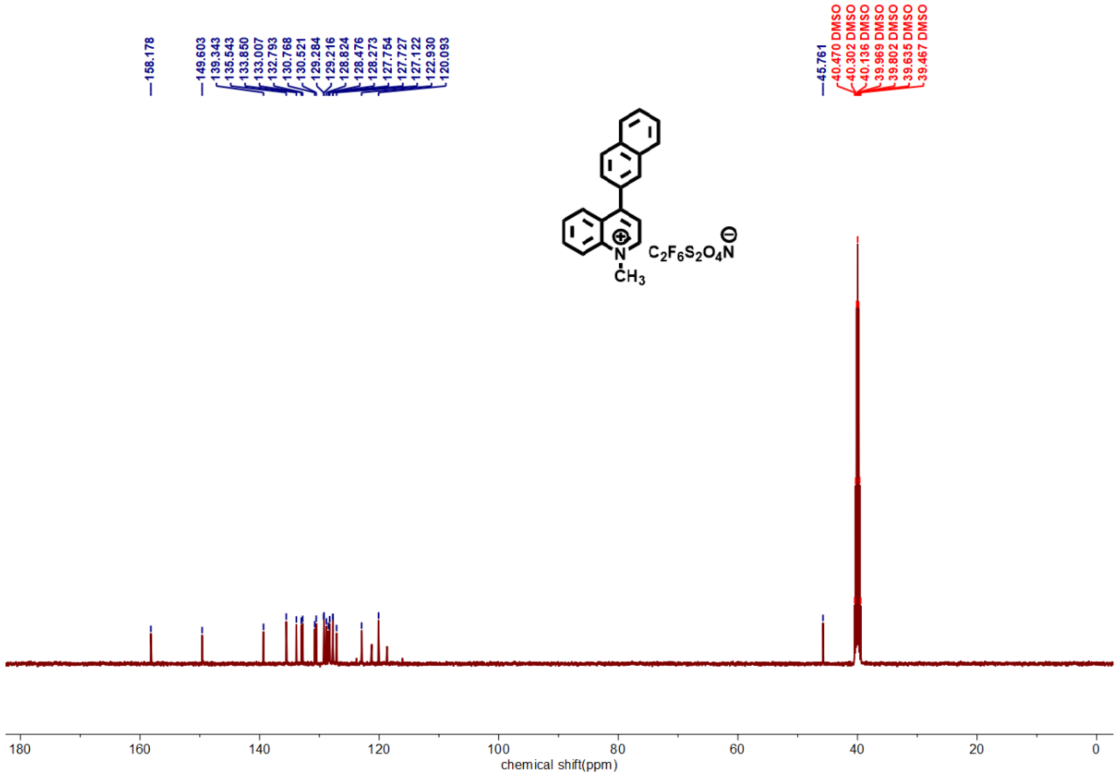

Figure S91. ^{13}C NMR of **QL-B** (DMSO-*d6*, 126 MHz).

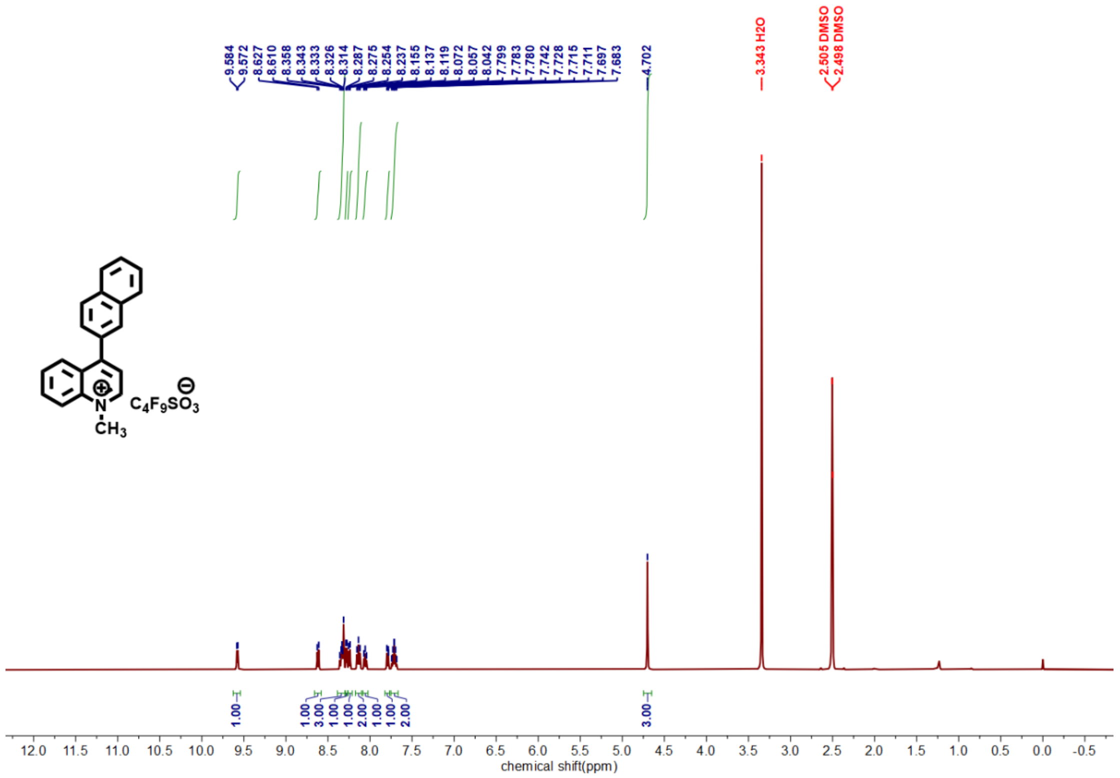

Figure S92. ^{1}H NMR of **QL-C** (DMSO-*d6*, 500 MHz).

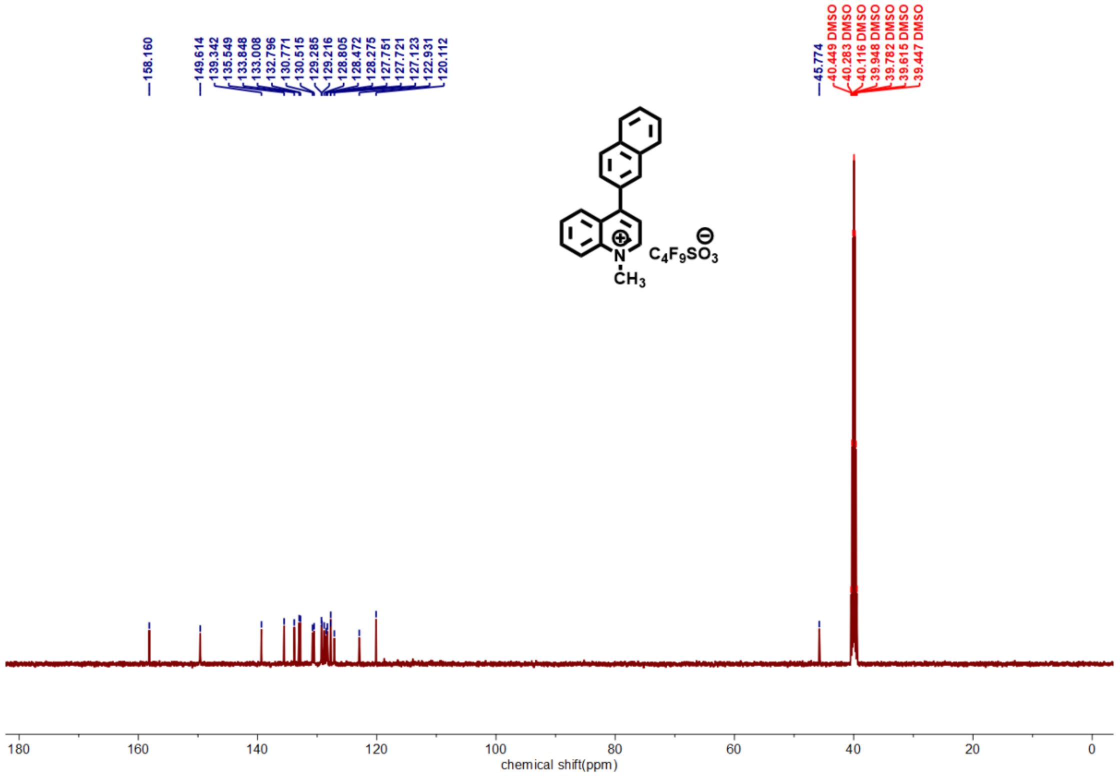

Figure S93. ^{13}C NMR of **QL-C** (DMSO-*d6*, 126 MHz).

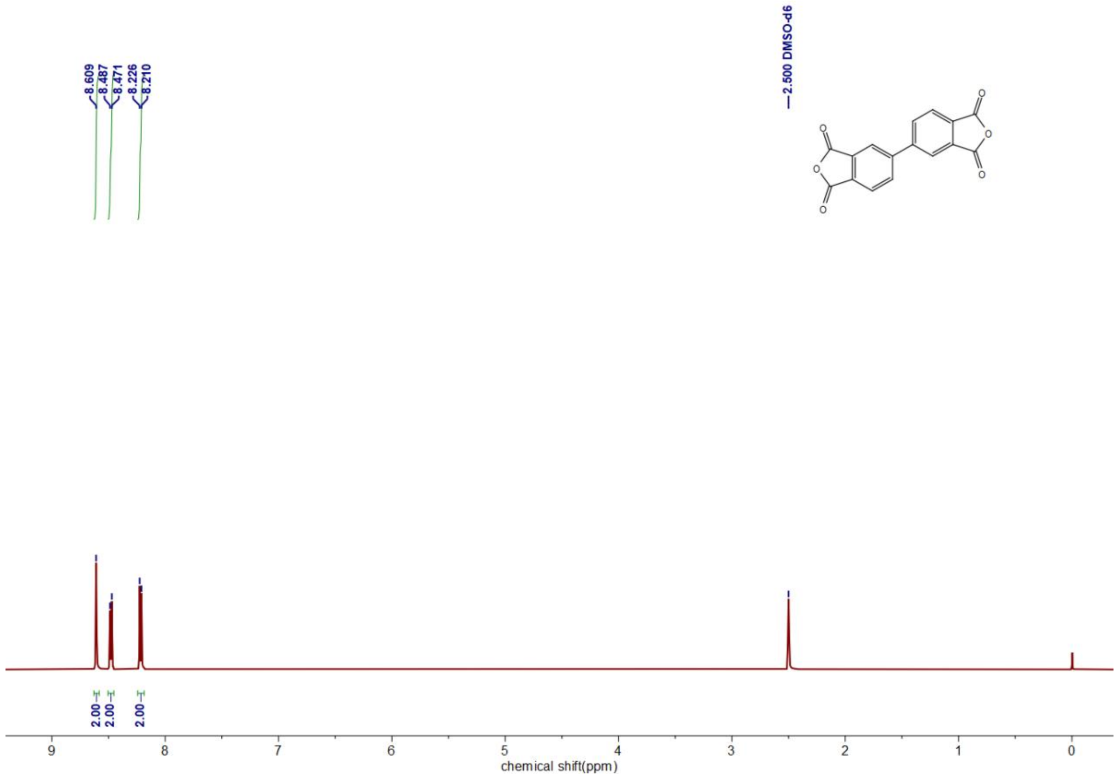

Figure S94. ^{1}H NMR of **BFT** (DMSO-*d6*, 500 MHz).

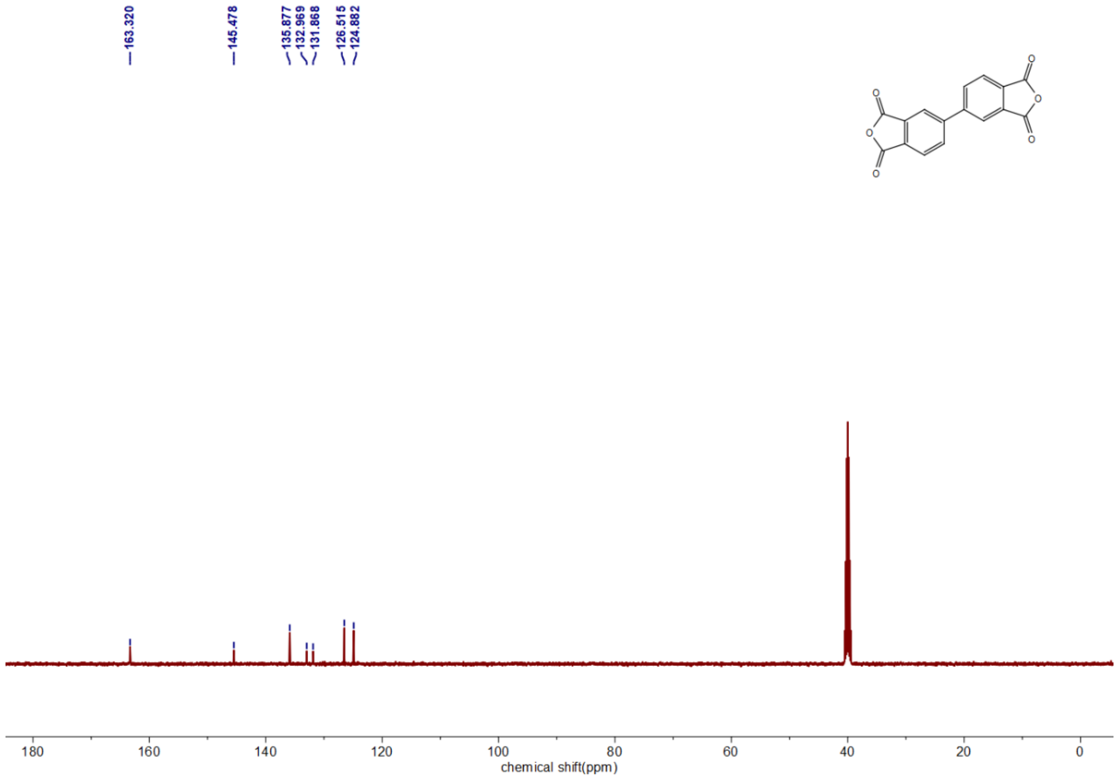

Figure S95. ^{13}C NMR of **BFT** (DMSO-*d6*, 126 MHz).

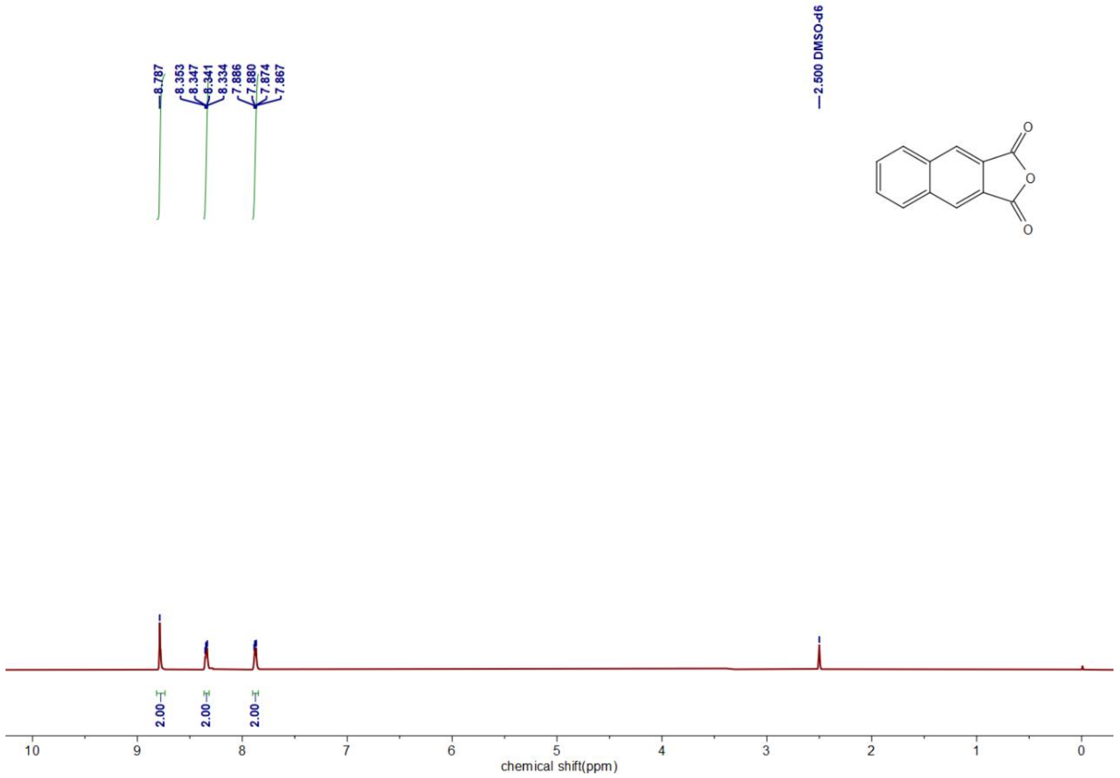

Figure S96. ^{1}H NMR of **NAFD** (DMSO-*d6*, 500 MHz).

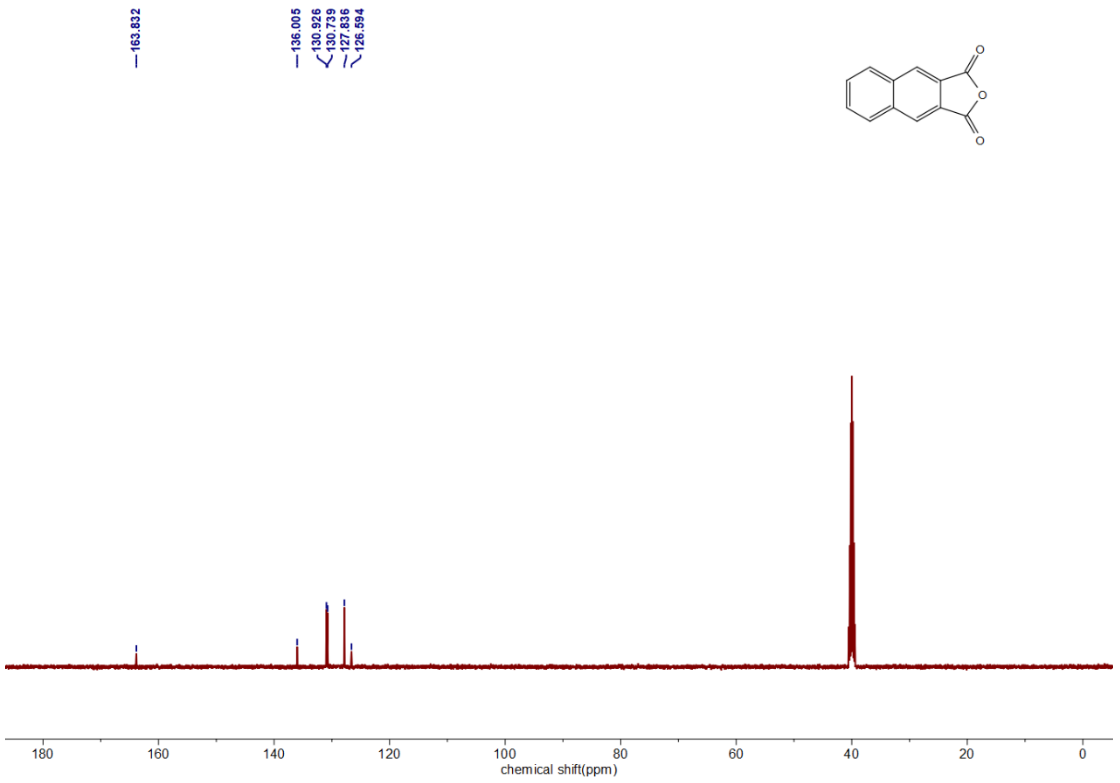

Figure S97. ^{13}C NMR of **NAFD** (DMSO-*d6*, 126 MHz).

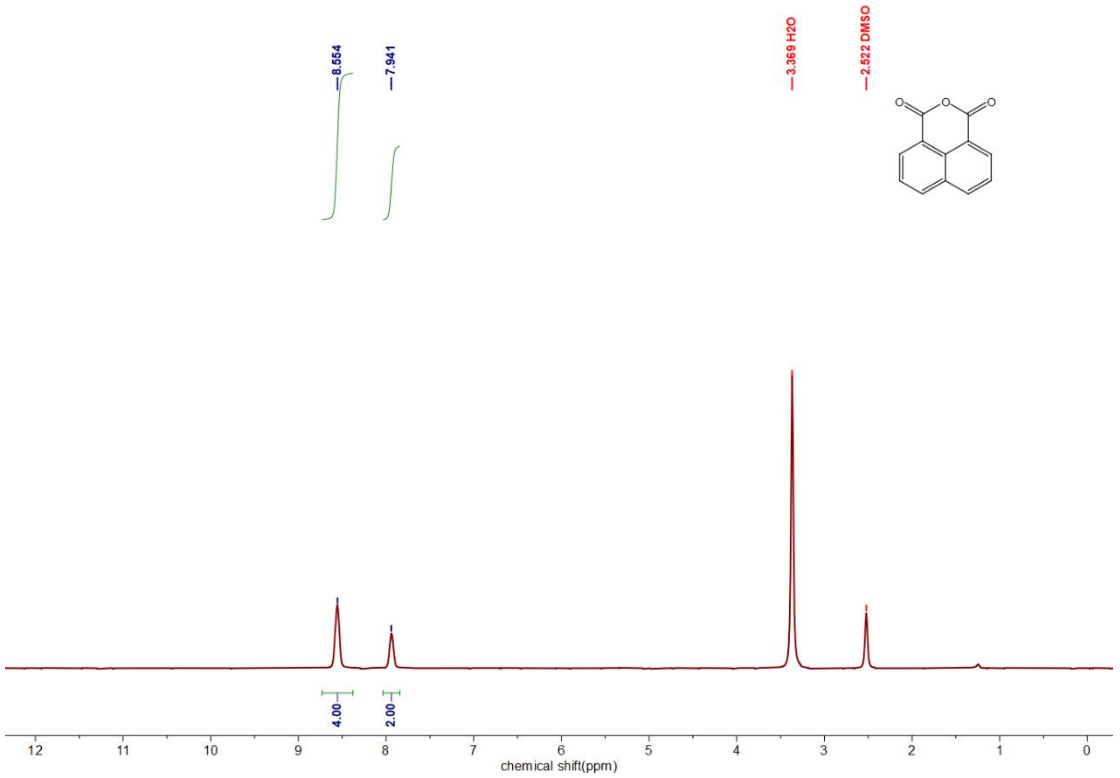

Figure S98. ^{1}H NMR of **BICD** (DMSO-*d6*, 500 MHz).

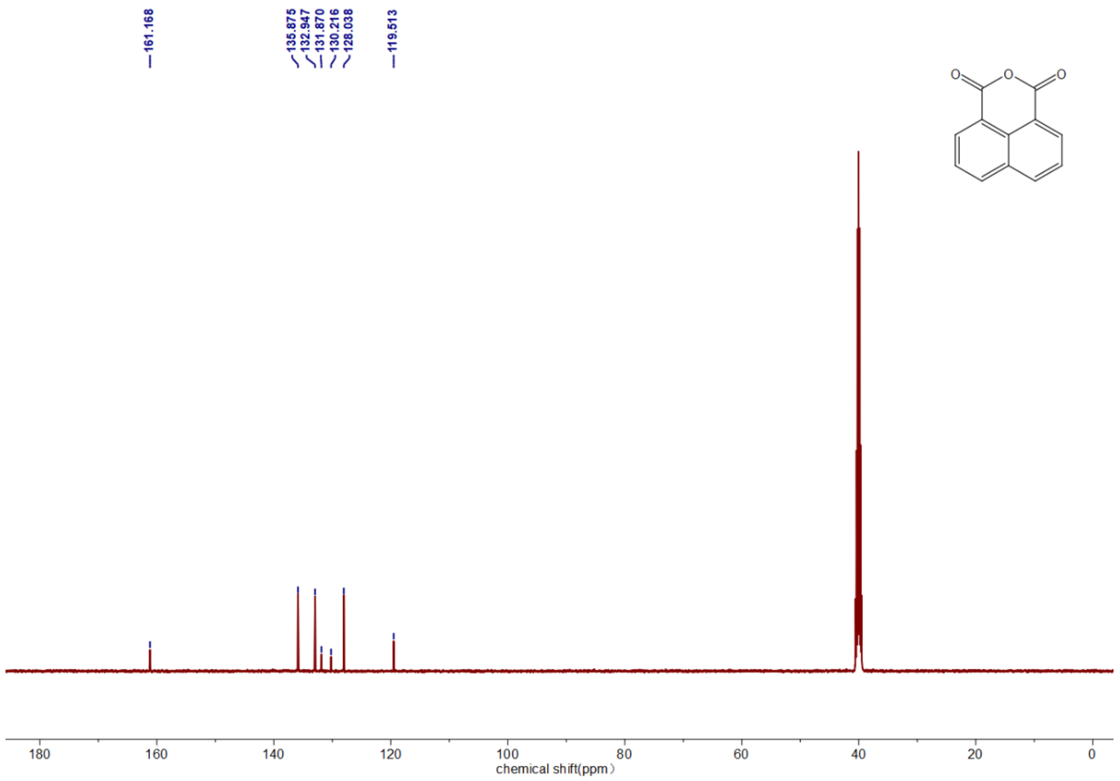

Figure S99. ^{13}C NMR of **BICD** (DMSO-*d6*, 126 MHz).

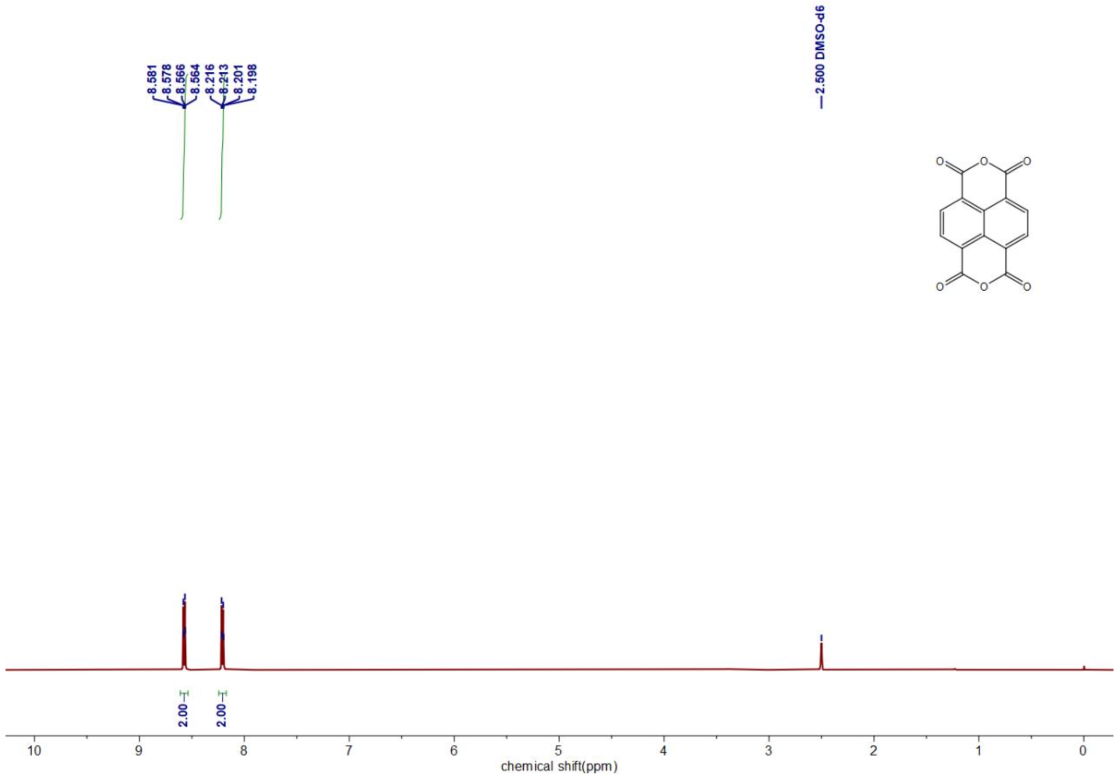

Figure S100. ^{1}H NMR of **CCT** (DMSO-*d6*, 500 MHz).

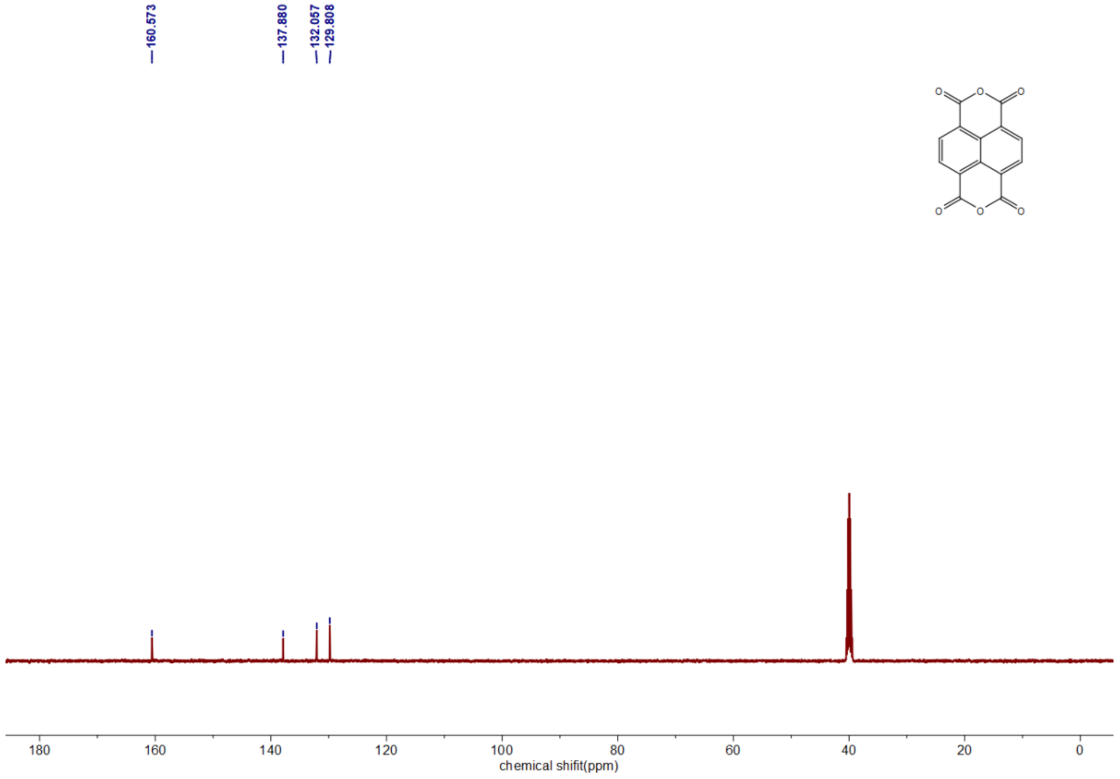

Figure S101. ^{13}C NMR of **CCT** (DMSO-*d6*, 126 MHz).

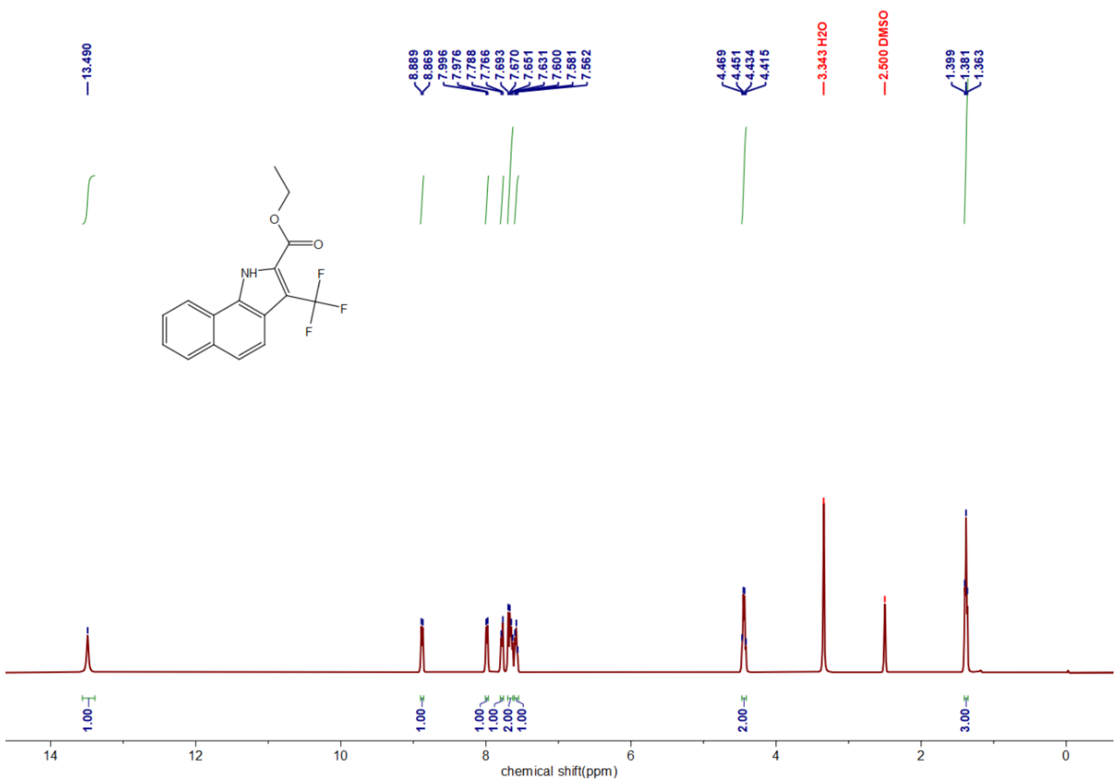

Figure S102. ^{1}H NMR of **BDC-CF$_3$** (DMSO-*d6*, 400 MHz).

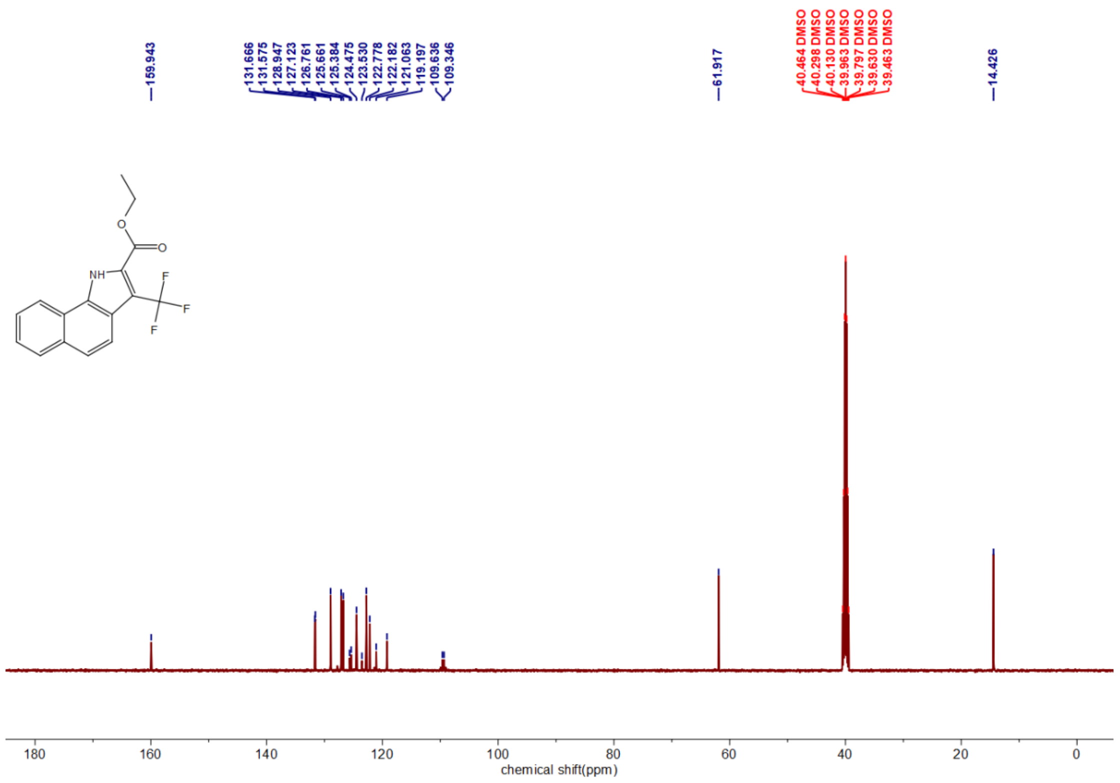

Figure S103. ^{13}C NMR of **BDC-CF$_3$** (DMSO-*d6*, 126 MHz).

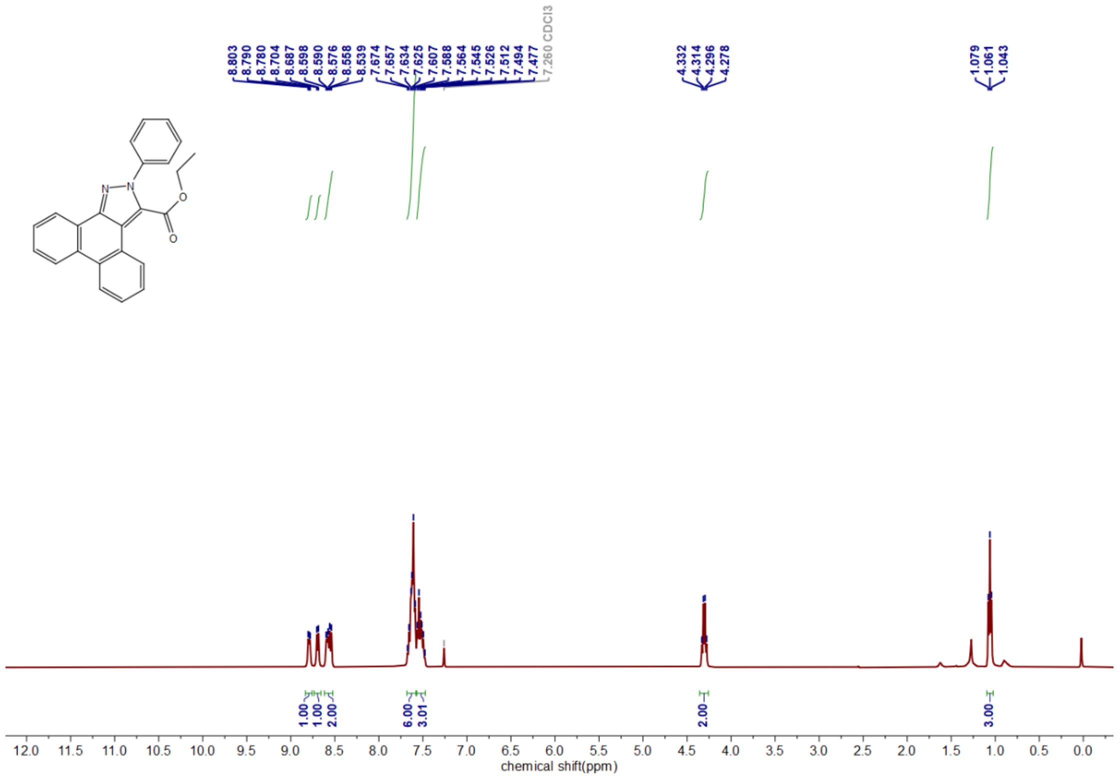

Figure S104. ^{1}H NMR of **IZ-EG** ($CDCl_3$, 400 MHz).

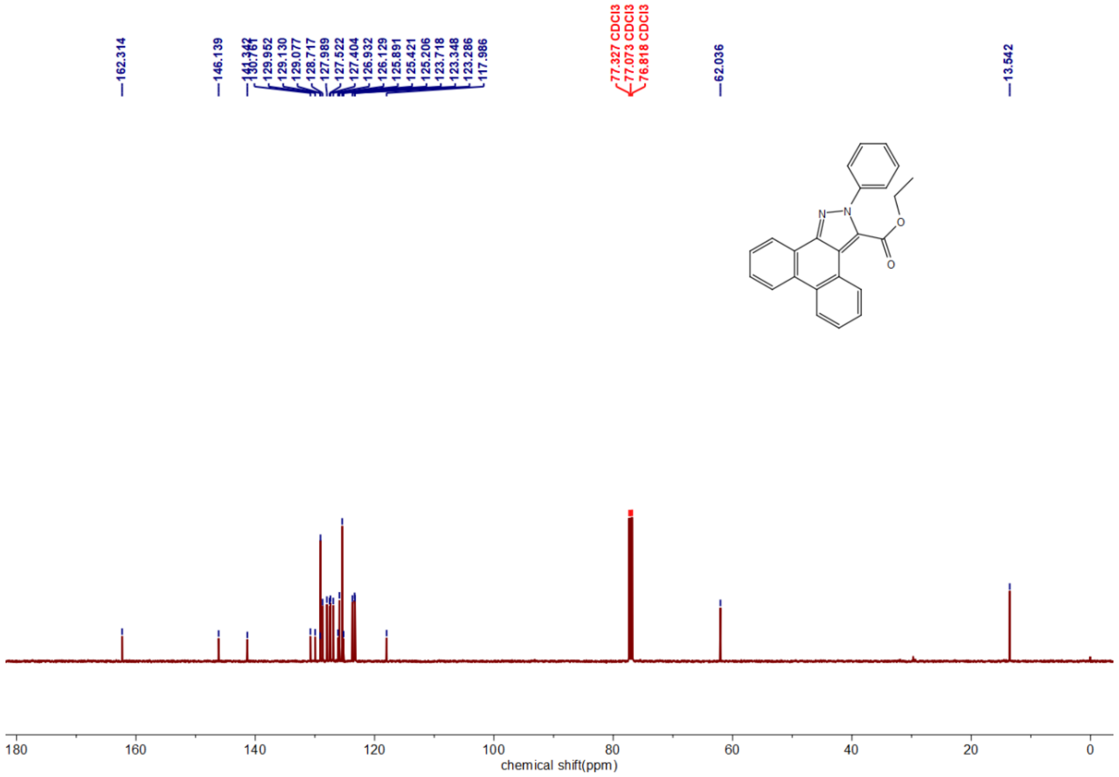

Figure S105. ^{13}C NMR of **IZ-EG** ($CDCl_3$, 126 MHz).

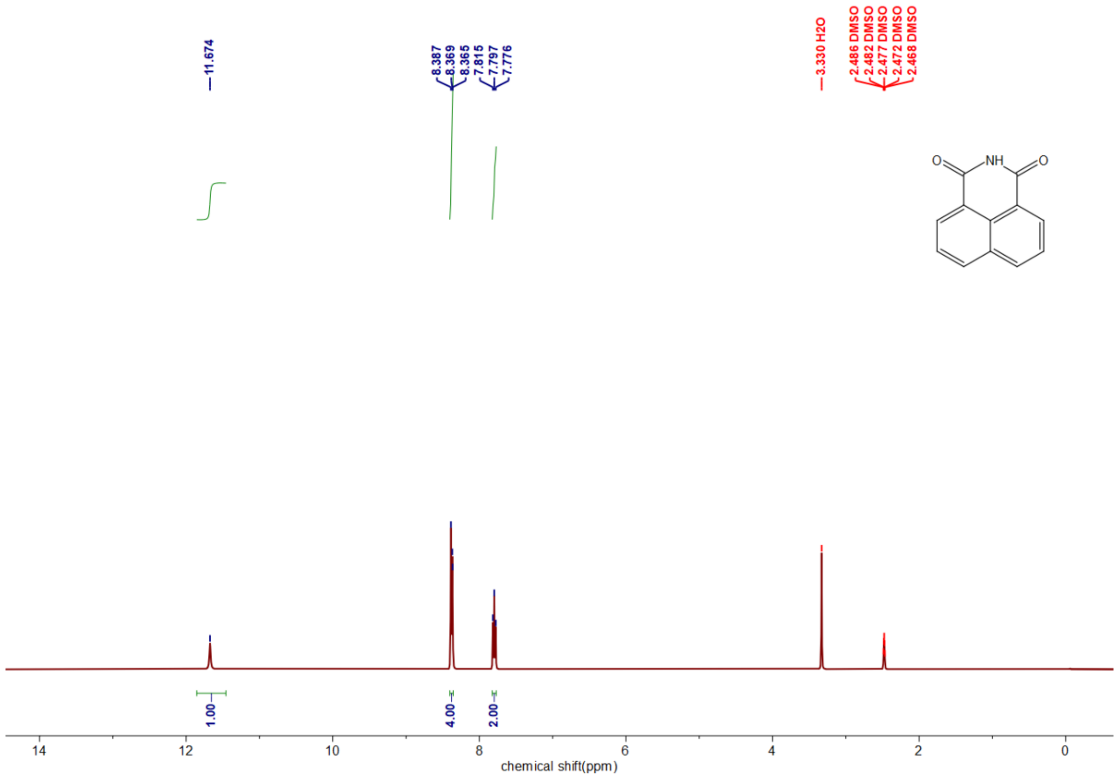

Figure S106. ^{1}H NMR of **NPA** (DMSO-*d6*, 400 MHz).

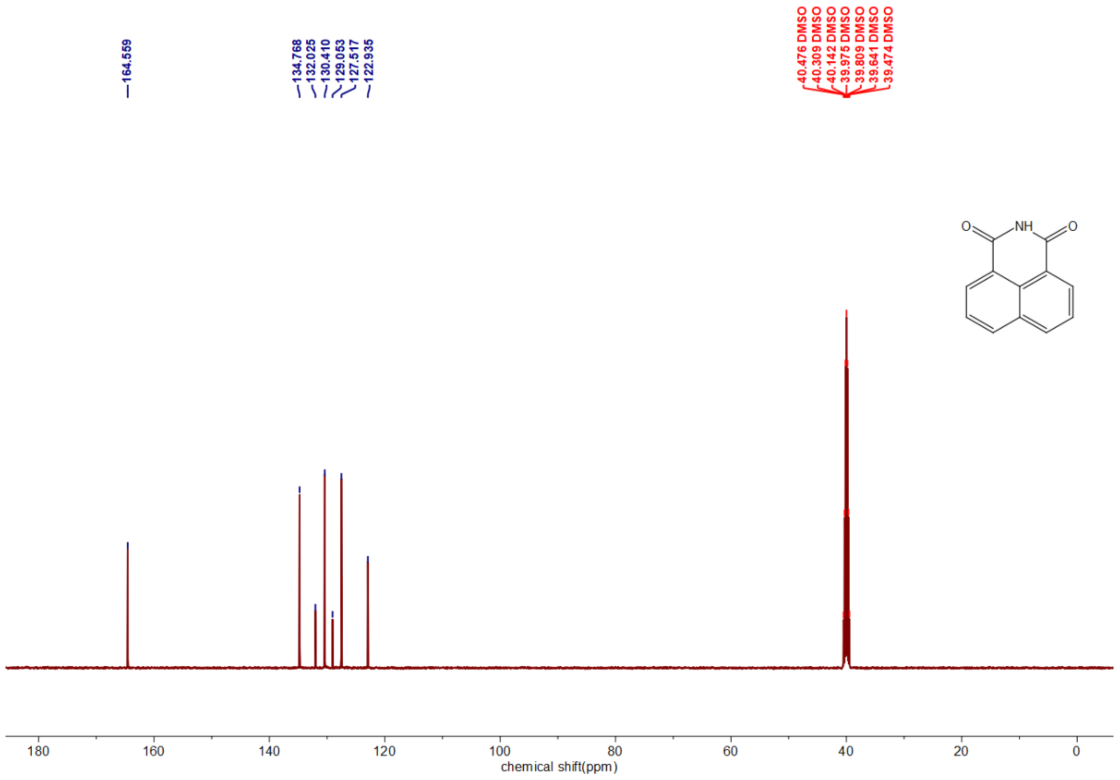

Figure S107. ^{13}C NMR of **NPA** (DMSO-*d6*, 126 MHz).

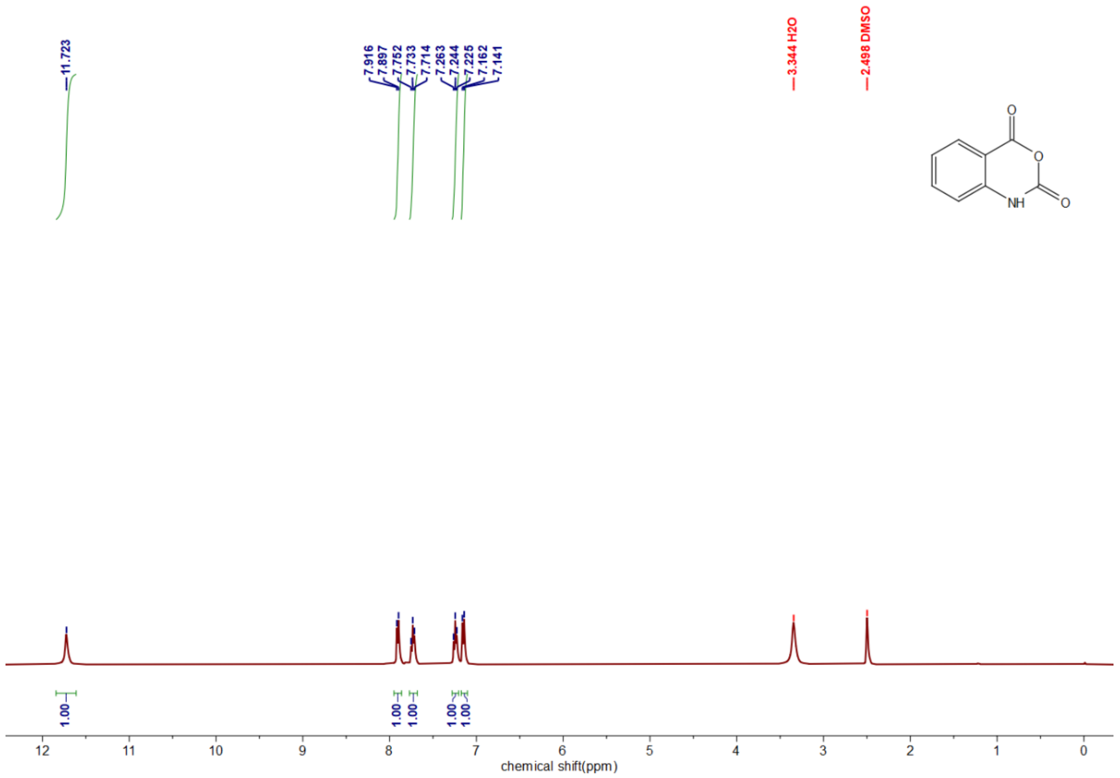

Figure S108. ^{1}H NMR of **1H-BOD** (DMSO-*d6*, 400 MHz).

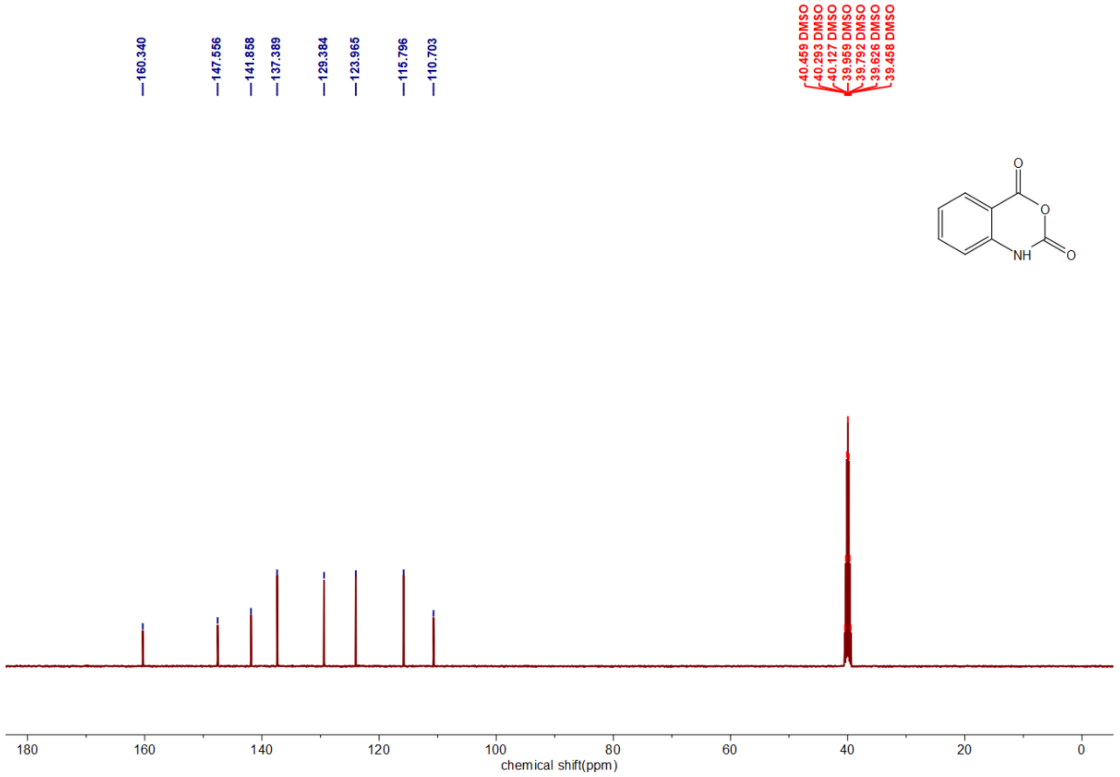

Figure S109. ^{13}C NMR of **1H-BOD** (DMSO-*d6*, 126 MHz).
